# Novel betulin derivatives as multidrug reversal agents targeting P-glycoprotein

**DOI:** 10.1038/s41598-023-49939-9

**Published:** 2024-01-02

**Authors:** Jerónimo Laiolo, Dafni G. Graikioti, Cecilia L. Barbieri, Mariana B. Joray, Antonia I. Antoniou, D. Mariano A. Vera, Constantinos M. Athanassopoulos, María C. Carpinella

**Affiliations:** 1 Fine Chemical and Natural Products Laboratory, IRNASUS CONICET-UCC, Universidad Católica de Córdoba, Córdoba, Argentina; 2 Synthetic Organic Chemistry Laboratory, Department of Chemistry, University of Patras, 26504 Patras, Greece; 3 Department of Chemistry and Biochemistry, College of Exact and Natural Sciences, Universidad Nacional de Mar del Plata - QUIAMM – INBIOTEC CONICET, Mar del Plata, Argentina; 4 Fine Chemical and Natural Products Laboratory, IRNASUS CONICET-UCC and CIDIE CONICET-UCC, Universidad Católica de Córdoba, Córdoba, Argentina

**Keywords:** Medicinal chemistry, Chemical synthesis, Drug screening, Biochemistry

## Abstract

Chemotherapy is a powerful means of cancer treatment but its efficacy is compromised by the emergence of multidrug resistance (MDR), mainly linked to the efflux transporter ABCB1/P-glycoprotein (P-gp). Based on the chemical structure of betulin, identified in our previous work as an effective modulator of the P-gp function, a series of analogs were designed, synthesized and evaluated as a source of novel inhibitors. Compounds **6g** and **6i** inhibited rhodamine 123 efflux in the P-gp overexpressed leukemia cells, K562/Dox, at concentrations of 0.19 µM and 0.39 µM, respectively, and increased the intracellular accumulation of doxorubicin at the submicromolar concentration of 0.098 µM. Compounds **6g** and **6i** were able to restore the sensitivity of K562/Dox to Dox at 0.024 µM and 0.19 µM, respectively. Structure–activity relationship analysis and molecular modeling revealed important information about the structural features conferring activity. All the active compounds fitted in a specific region involving mainly transmembrane helices (TMH) 4–6 from one homologous half and TMH 7 and 12 from the other, also showing close contacts with TMH 6 and 12. Compounds that bound preferentially to another region were inactive, regardless of their free energy of binding. It should be noted that compounds **6g** and **6i** were devoid of toxic effects against peripheral blood mononuclear normal cells and erythrocytes. The data obtained indicates that both compounds might be proposed as scaffolds for obtaining promising P-gp inhibitors for overcoming MDR.

## Introduction

In recent decades, chemotherapy has emerged as the hope for improving the outcome of cancer^1,2^. While many of the chemotherapeutic and targeted therapy drugs show early clinical benefits with improved overall survival, a large number of patients develop resistance^3,4^. As a result, cells have become less sensitive to the therapeutic effects of a broad array of agents, which is known as multidrug resistance (MDR), and poses a formidable challenge in clinical anticancer practices^5,6^, including first-line treatments of leukemia^7,8^.

Chronic myelogenous leukemia (CML) is a malignant myeloproliferative disorder^9^ which is successfully treated with tyrosine kinase inhibitors^10,11^. However, the effectiveness of these drugs and of doxorubicin (Dox) is altered by the development of MDR, conferred through different molecular mechanisms and pathways^11–13^. Among the factors involved in the genesis of this phenomenon, the outward transport of anticancer drugs mediated by transmembrane transporters plays a key role^14,15^. Efflux proteins belonging to the ATP binding-cassette (ABC) family which have been recognized in humans are classified in 7 subfamilies namely ABCA to ABCG^14,16^. Among these, ABCB subfamily is composed by more than 10 transporters involved in the efflux of drugs, peptides and ions^18,19^. One of the most significant efflux protein is ABCB1/P-glycoprotein (P-gp)^15,17^, an ATP-active transporter able to extrude structurally different drugs, which limits their intracellular availability and hampers their therapeutic effect^20,21^. ABCB1/P-gp, thereafter P-gp, is composed of two transmembrane domains (TMD), each with six transmembrane α-helices (TMH) and a nucleotide binding domain (NBD)^22,23^. ATP-hydrolysis represents the driving force for the TMD to switch between the inward- and the outward-facing conformations, which leads to the removal of the chemotherapeutic agents^23^.

Although diverse chemical entities grouped in three generations of inhibitors have been submitted to clinical trials, none of them has been successful. The first-generation of inhibitors such as verapamil or cyclosporine A with phenylethylamine and cyclic undecapeptide scaffolds, respectively, were ineffective or showed side effects associated with their pharmacological uses^24,25^. The second-generation of P-gp inhibitors such as the cyclosporine D analogue, PSC833, showed improved effectiveness and selectivity, however these entities exhibited undesired drug-drug interactions^23,25,26^ while the third-generation of modulators such as tariquidar, revealed more affinity to P-gp and low pharmacokinetic interactions, but their cytotoxicity led to therapeutic failures^23,25,26^. These major drawbacks, make the development of suitable novel agents capable of circumventing MDR/P-gp an urgent necessity.

There is ample evidence that plant-derived substances exhibit privileged chemical structures plausible for obtaining novel derivatives^27,28^. Many of these, including some obtained from Argentinian plants, have improved the efficacy of anticancer drugs by inhibiting the P-gp transporter function^29–32^.

In a previous work, we found that betulin (**1**) was a potent compound capable of modulate P-gp^32^, which established its chemical structure as a promising scaffold to obtain derivatives with improved activity. In other publication, we observed that the increasing number of methoxy substituents as well as the presence of a flexible linker, such as the –NHCH_2_– group, favored the P-gp inhibitory effect^33^. In addition, known first and third generation of P-gp inhibitors including verapamil, tariquidar and elacridar, bear OCH_3_ groups on their aromatic moieties^34,35^. Based on this data and considering pentacyclic triterpenes as an encouraging chemical skeleton, a series of novel compound **1** derivatives were designed and synthesized. For comparison purposes, the work includes also the synthesis of a derivative from the inactive compound betulinic acid (**2**)^32^. The chemical entities obtained were then evaluated in order to find the best candidates for P-gp inhibition.

## Results and discussion

### Synthetic chemistry

As previously described, compound **1**, bearing a primary alcohol at position C-28, was shown to be a promising MDR reversal agent due to the inhibition on P-gp, while betulinic acid (**2**), containing a C-28 carboxyl group, completely lacked this property^32^. Based on these findings, taking into consideration the information regarding the pharmacophores and the contacts favoring anti-P-gp activity^33^ and the fact that the *O-*methylation of polyphenolic rings, which leads to increased lipophilicity, seems to be a key modification to obtain effective P-gp modulators^36–39^, a library of betulin derivatives was prepared. Modifications of the position C-28 to yield esters, an amide, and carbamates suitable for structure–activity relationship studies (SARs), were the key changes performed in this series of compounds. The fact that carbamates exhibit proteolytic stability in combination with their ability to penetrate cell membranes^40^ makes this functionality a valuable option to include in novel compounds.

The derivatives were designed to be suitable to (a) compare the effect of the C-28 bonding functional group (ester or carbamate), (b) confirm the influence of the number of methoxy substituents in the aromatic ring as well as the presence of other rings, and (c) study the length of the saturated or unsaturated carbon chain connecting the C-28 of compound **1** and the aromatic ring counterpart. Finally, the replacement of the methoxy-substituted aromatic ring by other heterocycles, such as substituted piperazines or tetrahydroquinoline, enriched the library and the expected SAR study.

**Figure 1:**
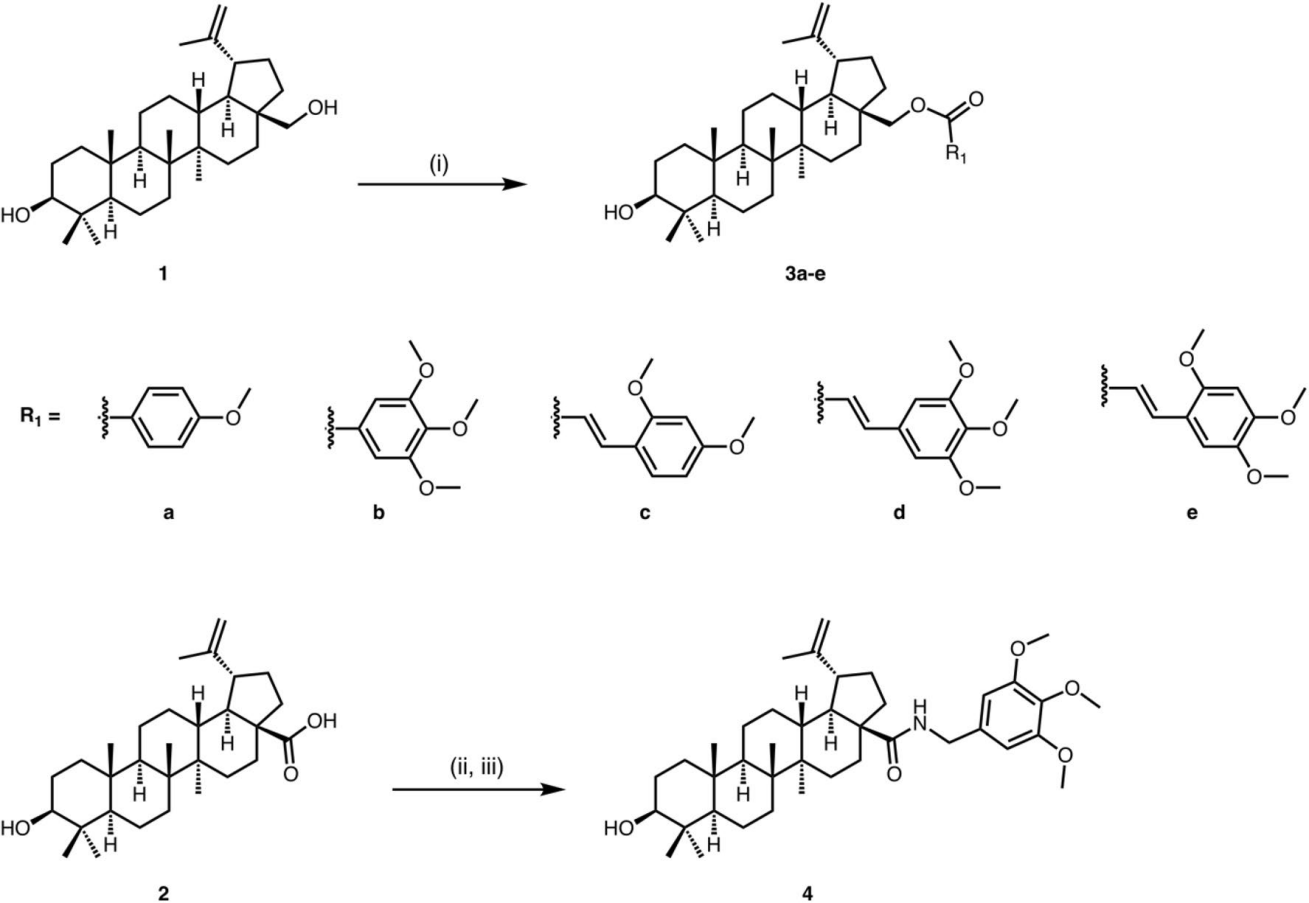
Synthesis of betulin esters **3a-e** and of the betulinic acid amide **4**; Reagents and conditions: (i) corresponding acid, DCC, 4-dimethylaminopyridine (DMAP), dichloromethane (DCM)/tetrahydrofuran (THF) (3:2), 0 °C to rt, overnight, 47–98%; (ii) HOBt, DCC, THF, 0 °C to rt, 15 h, 88%; (iii) 3,4,5-trimethoxybenzylamine, THF, 0 °C to rt, 16 h, 50%.

The synthesis of esters **3a-e** starts with a Steglich esterification of compound **1** with mono-, di- and tri-methoxy substituted aromatic acids, providing moderate to high yields of the corresponding esters (Fig. 1). In addition, treatment of compound **2** using the *N,N΄*-dicyclohexylcarbodiimide/hydroxybenzotriazole (DCC/HOBt) system led to the intermediate reactive ester, which provided the amide **4** upon reaction with 3,4,5-trimethoxybenzylamine (Fig. 1).

**Figure 2:**
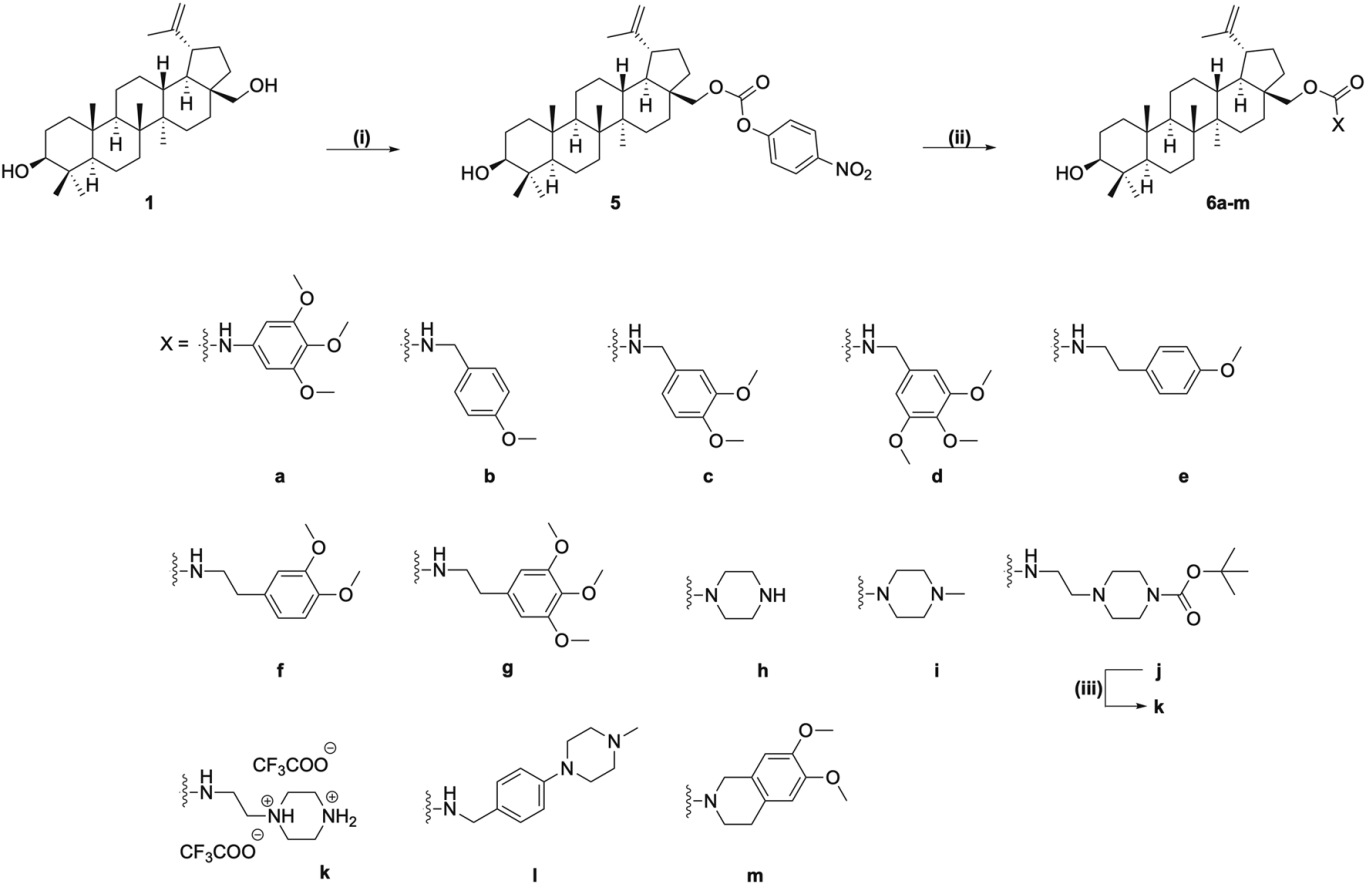
Synthesis of the betulin carbamates **6a**-**m**; Reagents and conditions: (i) *p*-nitrophenylchloroformate, pyridine, THF, 0 °C to rt, overnight, 83%; (ii) corresponding amine, DIPEA, THF, rt to 50–60 0 °C, 42–75%; (iii) TFA, 2,2,2-trifluoroethanol (TFE), DCM, 0 °C to rt, 3 h, 90%.

To prepare the proposed derivatives **6a-j**, **6l** and **6m**, compound **1** was activated to the *p*-nitrophenyl carbonate **5**, which upon treatment with the corresponding amines in presence of *N,N΄*-diisopropylehtylamine (DIPEA) gave the expected carbamates in 42–75% yields (Fig. 2). Trifluoroacetic acid (TFA)-mediated Boc-group removal from compound **6j** afforded the corresponding carbamate **6k**.

### Cytotoxic activity of the compounds in sensitive and resistant leukemia cells

To determine the effect of the proposed library on proliferation and to establish the appropriate concentrations to be used in the P-gp modulatory assays, the colorimetric MTT experiment was performed on resistant (K562/Dox) and sensitive (K562) CML cell lines.

As observed in Table 1, compounds **3a**-**e**, **6a**-**g**, **6j** and **6m** displayed no inhibition on proliferation of K562/Dox and K562 at the highest concentration assayed of 50 μM. Since compounds **4**, **6h**, **6i**, **6k,** and **6l** produced more than 55% toxicity at this concentration in both cell lines, their half-maximal inhibitory concentration (IC_50_) values were obtained, and were 19.7, 4.6, 19.1, 9.4, and 8.0 μM, respectively, in K562 cells. These results indicated that compounds **6h**, **6k,** and **6l** were toxic to this cell line, according to the upper limit of toxicity of 10 μM proposed by US National Cancer Institute (NCI)^41^. By contrast, compounds **4**, **6h**, **6i**, **6k,** and **6l** showed no cytotoxicity toward K562/Dox, with IC_50_ values ranging from 12.5 to 34.4 μM. Regarding verapamil and the third-generation reference P-gp blocker, tariquidar^42^, the first was not toxic at 50 μM, while the latter exhibited cytotoxicity on K562/Dox and K562 cells with IC_50_ values of 2.2 and 2.4 μM, respectively. These results suggest that the derivatives **3b**, **4** and **6a**-**6g**, **6i-6m** are safer candidates for P-gp/MDR reversal agents than tariquidar.

**Table 1:** Antiproliferative effect of the derivatives (**3a**-**3e**, **4** and **6a**-**6l**) on multidrug-resistant, K562/Dox cells and its sensitive counterpart, K562.

Compounds	IC_50_ (μM)	
	K562/Dox	K562
**3a**	> 50	> 50
**3b**	> 50	> 50
**3c**	> 50	> 50
**3d**	> 50	> 50
**3e**	> 50	> 50
**4**	34.41 ± 2.59	19.70 ± 0.59
**6a**	> 50	> 50
**6b**	> 50	> 50
**6c**	> 50	> 50
**6d**	> 50	> 50
**6e**	> 50	> 50
**6f**	> 50	> 50
**6g**	> 50	> 50
**6h**	12.50 ± 0.50	4.60 ± 0.24
**6i**	33.91 ± 0.09	19.13 ± 0.87
**6j**	> 50	> 50
**6k**	27.00 ± 1.34	9.44 ± 0.08
**6l**	17.41 ± 0.59	8.00 ± 1.98
**6m**	> 50	> 50
Verapamil	> 50	> 50
Tariquidar	2.25 ± 0.28	2.38 ± 0.32

Values are expressed as mean ± SE of at least three independent experiments.

### Modulating effects of the candidates on P-gp by the rhodamine 123 and doxorubicin intracellular accumulation assays

The need for new entities with novel scaffolds to overcome MDR drives efforts to test compounds prepared in accordance with encouraging data previously obtained^32,33^. Compounds derived from the isolated plant-derived molecules **1** and **2**, at sub-cytotoxic doses, were subjected to the rhodamine 123 (Rho123) intracellular accumulation assay to determine their capacity as P-gp inhibitors. The absence of significant changes in cell shape, which is proportional to cell size, as measured by forward-scattered light (FSC) and cell granularity (side-scattered light, SSC)^43^, indicated that the target compounds did not induce signs of cytotoxicity at the assayed concentrations in either cell line (data not shown).

**Figure 3:**
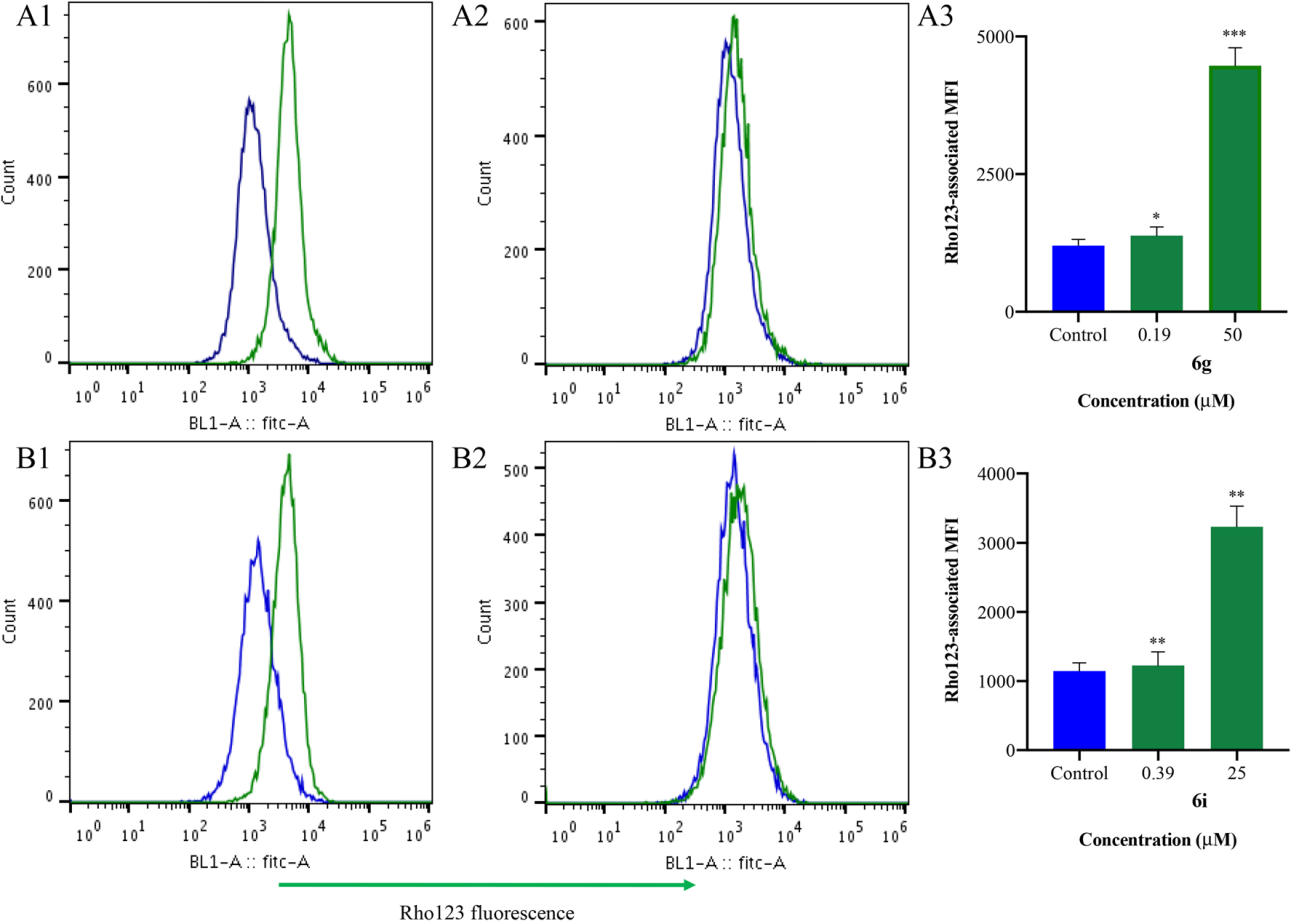
Representative histograms of the fluorescence intensity of cells treated with rhodamine 123 (Rho123, blue) or with Rho123 and compounds **6 g** (**A**) or **6i** (**B**) (green) at (A.1) 50 µM, (A.2) 0.19 µM, (B.1) 25 µM and (B.2) 0.39 µM in K562/Dox cell line. The increase in Rho123-associated mean fluorescence intensity (MFI) could be observed in K562/Dox cells treated with compounds **6g** (A3) and **6i** (B3), respectively.

**Figure 4:**
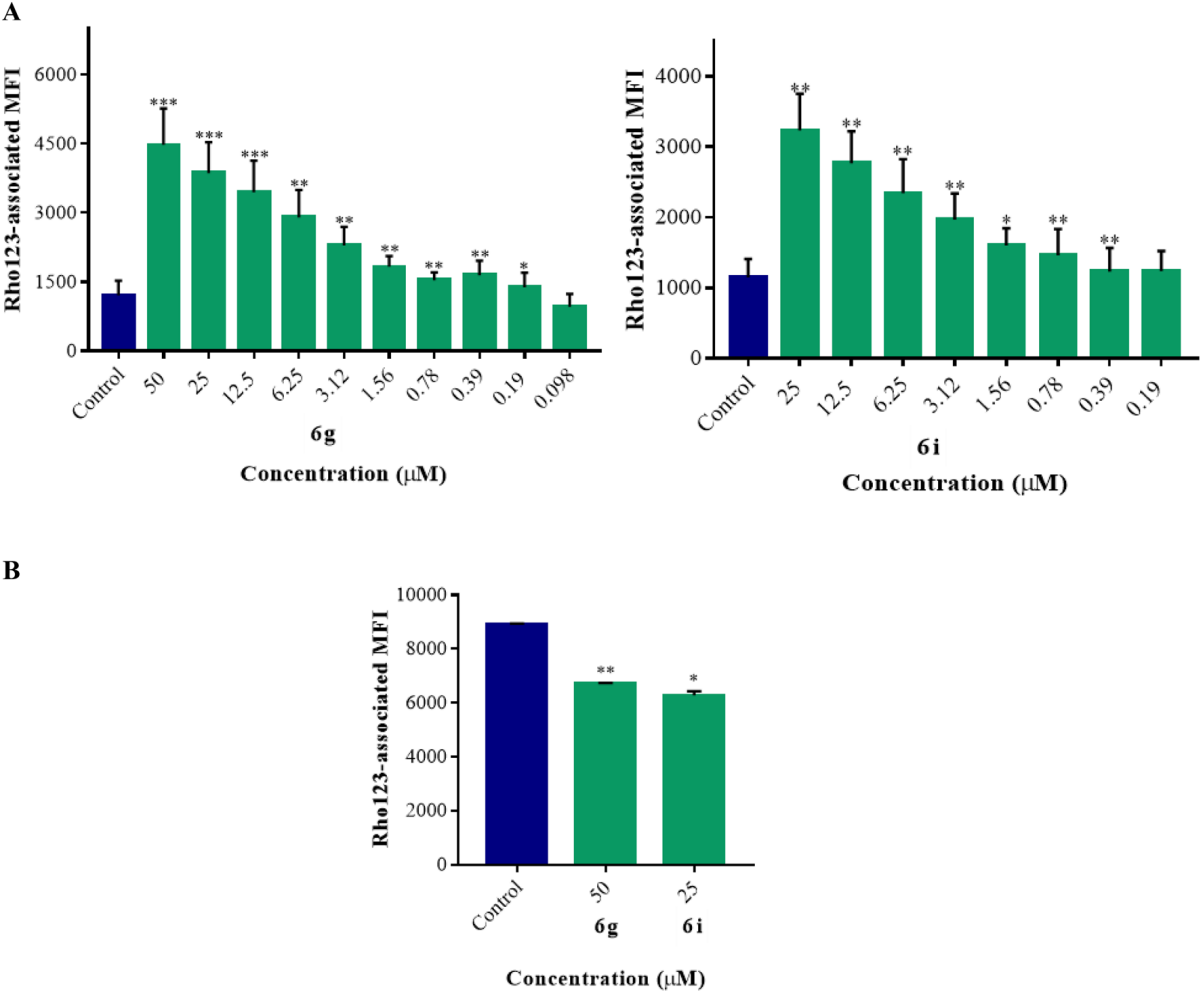
In the rhodamine 123 (Rho123) accumulation assay, the intracellular accumulation of the dye was significantly augmented by treatment with different concentrations of compounds **6g** and **6i** in K562/Dox cells (**A**). In K562 cells (**B**), the respective compounds did not increase Rho123 accumulation. Significant differences from the negative control were determined using the one-tailed paired Student's *t*-test (***p < 0.001, **p < 0.01, *p < 0.05).

As observed in Table 2, derivatives **3a**, **3c**, **3d**, **3e,** and **6h** did not induce statistically significant Rho123 accumulation (*p* > 0.05) at the highest concentrations tested (50 or 6.25 µM). However, compounds **3b**, **6a**-**6g**,** 6j,** and **6m** at 50 μM significantly increased Rho123 retention in K562/Dox (*p* < 0.001–0.05), with fluorescence activity ratio (FAR) values ranging from 1.1 to 4.0 (Table 2). Compounds **4**, **6i,** and **6k** efficiently enhanced the intracellular accumulation of the dye (*p* < 0.01–0.05) at 25 µM with FAR values of 1.3–3.0, while compound **6l** did the same (*p* < 0.01) at 12.50 µM. The most effective compounds **6k**, **6d**, **6i** and **6g** were able to modulate P-gp with FAR values ranging from 2.1 to 3.1 even at 12.50 µM. In view of these results, the active candidates were subsequently tested at successive two-fold dilutions to establish their minimum effective concentrations (MECs). Derivatives **3b**, **6b,** and **6e** were weakly active at 50 μM, while compounds **4, 6j,** and **6l** showed weak inhibition of the Rho123 efflux (*p* < 0.01–0.05) at 25, 12.50 and 6.25 µM, respectively (Table 2). Although compound **4** showed itself weakly active, its reversal activity was greater than that observed for its lead compound **2**, which was inactive at 50 µM^32^. The latter result matched that described by Delou et al. 2009^44^ who found compound **2** not to alter Rho123 accumulation in MA-104 cells when applied at 22 µM. The rest of the derivatives exhibited very low MEC values (*p* < 0.01–0.05, Table 2). Compounds **6c** and **6m** presented MECs of 3.12 µM, which is higher than that observed for compound **1** (MEC = 1.56 µM)^32^. Compounds **6a, 6f** and **6k** inhibited the outward transport of Rho123 (Table 2) at the same level as compound **1**^32^. Surprisingly, derivatives **6d** and particularly compounds **6g** and **6i,** with MECs of 0.78, 0.19 and 0.39 μM, respectively, exceeded the activity of compound **1**, verapamil and tariquidar, the latter two with MEC values of 0.39 and 0.78 μM, respectively. No statistical differences were observed (*p* > 0.05) when compounds **6g** and **6i** were compared to verapamil at the same concentration of 0.39 μM, or when compounds **6g** and **6i** at 0.78 μM were compared with tariquidar at the same concentration (*p* > 0.05). Derivatives **6g** and **6i** showed dose-dependent behavior (b = 64.49 p < 0.01; 95% CI 36.12 to 92.87, and b = 40.14* p* < 0.01; CI 25.02 to 55.26, respectively, Figs. 3 and 4A). The complete panel of active compounds caused no significant accumulation of Rho123 in K562 at the maximum concentration assayed (Table 2 and Fig. 4B). These concentrations were equal to or higher than the effective concentrations observed in K562/Dox (Table 2), confirming that the main mechanism by which the compounds increased the concentration of the dye was by inhibition of the P-gp pump.

**Table 2:** MDR-modulatory activity of the synthesized derivatives in K562/Dox and K562 leukemia cells by the Rho123 intracellular accumulation assay.

Compounds	FAR														
	K562/Dox										K562				
	Concentration (µM)										Concentration (µM)				
	50	25	12.50	6.25	3.12	1.56	0.78	0.39	0.19	0.098	50	25	12.50	6.25	3.12
**2**	1.05 ± 0.04														
**3a**	0.88 ± 0.02										1.03 ± 0.02				
**3c**	0.74 ± 0.05										0.88 ± 0.02				
**3d**	1.00 ± 0.06										1.02 ± 0.02				
**3e**	0.78 ± 0.01										0.90 ± 0**				
**6h**	–	–	–	1.06 ± 0.08							–	–	–	1.02 ± 0.03	
**3b**	1.06 ± 0.003*	0.94 ± 0.03									1.00 ± 0.03				
**6b**	1.60 ± 0.07*	0.90 ± 0.11									1.02 ± 0.02				
**6e**	1.53 ± 0.09**	1.04 ± 0.11									1.04 ± 0.05				
**4**	–	1.30 ± 0.09*	1.04 ± 0.01								–	0.88 ± 0.01*			
**6j**	2.57 ± 0.11***	1.68 ± 0.03**	1.25 ± 0.02**	1.08 ± 0.04							0.97 ± 0.05				
**6l**	–	–	1.55 ± 0.03**	1.30 ± 0.04**	1.08 ± 0.05								0.82 ± 0.07		
**6c**	1.97 ± 0.15**	1.65 ± 0.11*	1.40 ± 0.06*	1.23 ± 0.01*	1.13 ± 0.03*	1.00 ± 0.02					0.77 ± 0.02*				
**6m**	2.06 ± 0.15***	1.52 ± 0.09**	1.32 ± 0.14*	1.27 ± 0.09*	1.17 ± 0.03*	1.03 ± 0.12					1.01 ± 0.07				
**1**	2.77 ± 0.36**	2.34 ± 0.19****	1.80 ± 0.08****	1.32 ± 0.06**	1.23 ± 0.04**	1.12 ± 0.03*	1.01 ± 0.07			0.99 ± 0.09					
**6a**	1.62 ± 0.10**	1.33 ± 0.11*	1.20 ± 0.08*	1.12 ± 0.02**	1.12 ± 0.005*	1.17 ± 0.02*	0.98 ± 0.02				1.02 ± 0.02				
**6f**	2.27 ± 0.31**	1.95 ± 0.25**	1.73 ± 0.18**	1.45 ± 0.16*	1.25 ± 0.02**	1.13 ± 0.04*	1.02 ± 0.02				0.97 ± 0.01				
**6k**	–	3.02 ± 0.15**	2.96 ± 0.07**	2.24 ± 0.24**	1.48 ± 0.07*	1.35 ± 0.08*	1.03 ± 0.17				‘	-	-	0.64 ± 0.02	
**6d**	2.80 ± 0.32*	2.48 ± 0.29*	2.10 ± 0.16*	1.88 ± 0.17*	1.64 ± 0.16*	1.27 ± 0.08*	1.16 ± 0.03*	1.06 ± 0.08			0.94 ± 0.05				
**6i**	–	2.84 ± 0.19**	2.42 ± 0.13**	2.04 ± 0.05**	1.71 ± 0.06**	1.41 ± 0.14*	1.30 ± 0.06**	1.17 ± 0.01**	1.08 ± 0.05		0.75 ± 0**				
**6g**	4.02 ± 0.70***	3.47 ± 0.61***	3.11 ± 0.58***	2.62 ± 0.49**	2.05 ± 0.36**	1.61 ± 0.23**	1.33 ± 0.14**	1.42 ± 0.20**	1.09 ± 0.03*	1.00 ± 0.04	-	0.70 ± 0.01*			
Verapamil	6.19 ± 0.43*	5.36 ± 0.35*	4.91 ± 0.34*	4.13 ± 0.36*	3.02 ± 0.22**	2.33 ± 0.09*	1.78 ± 0.03***	1.82 ± 0.24*	1.06 ± 0.09		0.98 ± 0.05				
Tariquidar	–	–	–	–	1.14 ± 0.03**	1.22 ± 0.05***	1.12 ± 0.03*	1.06 ± 0.06							0.82 ± 0.11

*FAR*: fluorescence activity ratio.

Significant differences between treated and untreated cells were determined by using the one-tailed paired Student´s t-test (***p < 0.001, **p < 0.01, *p < 0.05). The MEC values of the different compounds are shown in bold. – not tested due to cytotoxicity.

**Figure 5:**
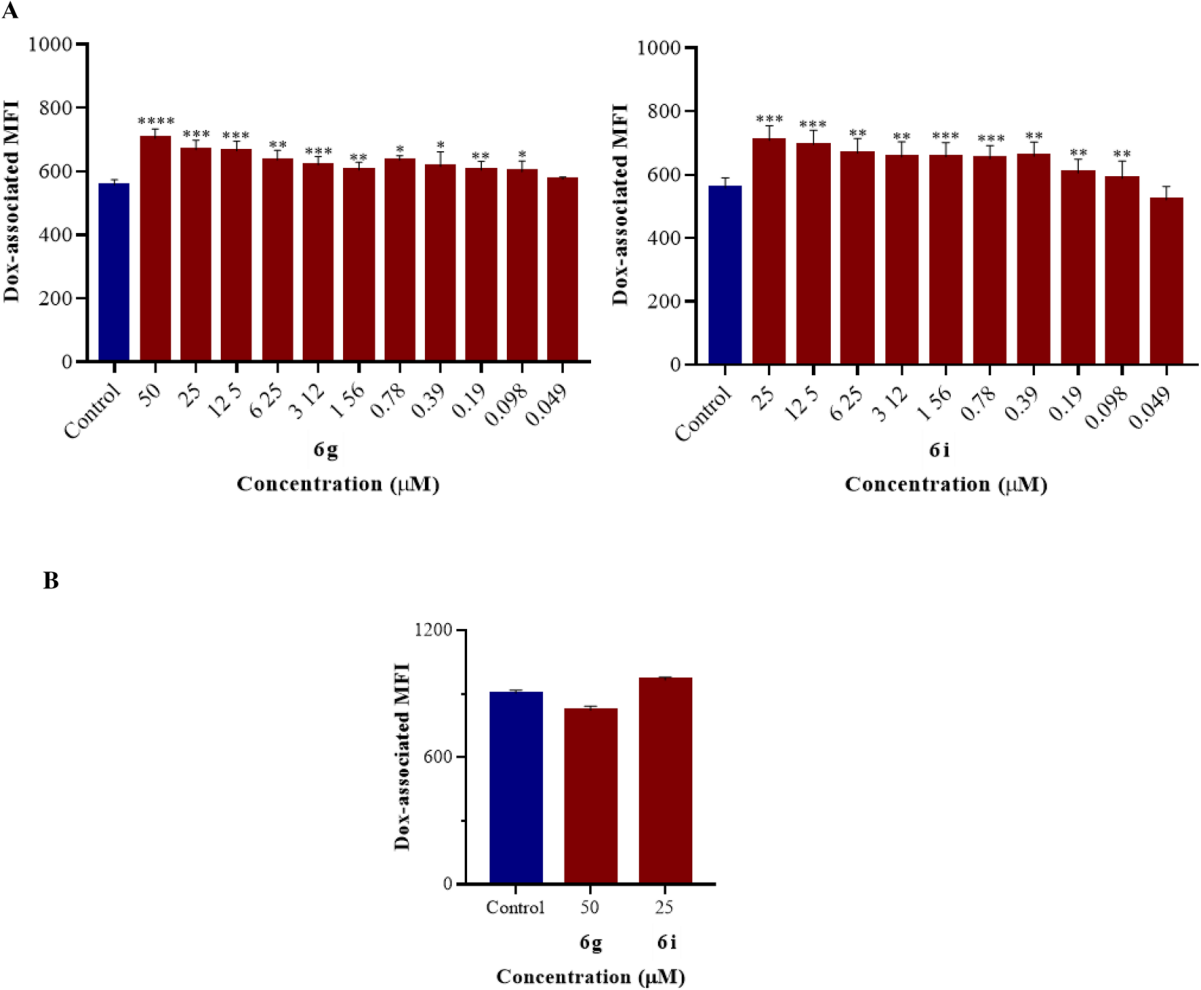
In the doxorubicin (Dox) accumulation assay, the intracellular accumulation of the dye was significantly augmented by treatment with different concentrations of compounds **6g** and **6i** in K562/Dox cells (**A**). In K562 cells (**B**), the respective compounds did not increase Dox accumulation. Significant differences from negative controls were determined using the one-tailed paired Student's *t*-test (****p < 0.0001, ***p < 0.001, **p < 0.01, *p < 0.05).

Since compounds **6g** and **6i** appeared as the most promising candidates, their anti-P-gp activity and mechanisms of inhibition were further investigated. To verify whether the blocking effect of both compounds on P-gp also applies to other substrates of the transporter, flow cytometry was conducted using the fluorescent drug, Dox^45^. Dox accumulation was substantially increased (*p* < 0.0001–0.05) in K562/Dox cells treated with 50 and 25 µM of compounds **6g** and **6i**, respectively (Fig. 5A), with FAR values of 1.26 ± 0.02 and 1.27 ± 0.03, respectively. Both compounds still caused a statistically significant retention (*p* < 0.01–0.05) of Dox when applied at the submicromolar concentration of 0.098 µM (Fig. 5A). This result shows compounds **6g** and **6i** to have higher activity than tariquidar, which displayed a MEC value of 0.39 µM. Comparing this reference compound at its MEC value with the same concentration of compounds **6g** and **6i,** no statistical differences were observed (*p* > 0.05). As seen in Fig. 5B, a similar Dox-accumulation (*p* > 0.05) was observed between K562 cells treated with compounds **6g** and **6i** and the negative control group, suggesting that a selective modulatory effect on P-gp led to the increase in Dox accumulation.

The inhibition of the efflux function of P-gp is consistent with previous studies reporting this property in other pentacyclic triterpenes, including glycyrrhizin, momilin, hederagenin and others, in most cases with active concentrations ranging from 5 to 500 µM^30,46,47^. This inhibitory effect was also observed in treatments with the structurally closely related lupane representative, lupeol, which proved effective at 100 µM^47^. The level of activity observed with compounds **6a**, **6f,** and **6k** is similar to that observed with the oleanane triterpene, oleanolic acid, showing effectiveness at 2 µM^48^.

### Doxorubicin resistance reversal assay

**Figure 6:**
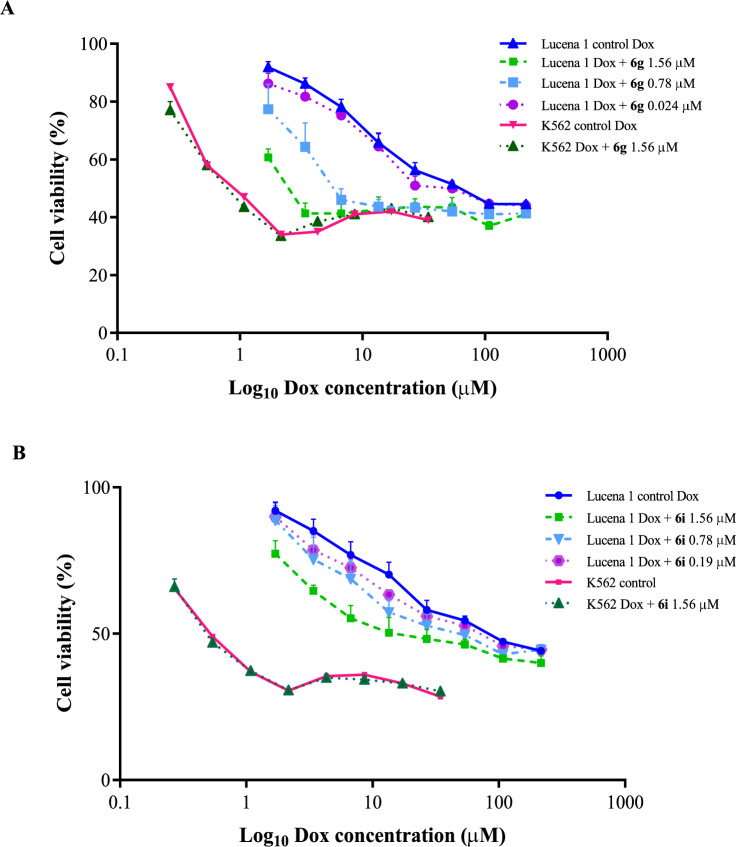
Dose–response curves for the ability of compounds **6g** (**A**) and **6i** (**B**) to sensitize K562/Dox cells to doxorubicin (Dox). This effect was significantly increased in the presence of **6g** and **6i** starting at 0.024 µM (*p* < 0.01) and 0.19 µM (*p* < 0.05), respectively. Assays performed on K562 discarded changes in Dox cytotoxicity by a mechanism other than the influence on P-gp. Values are expressed as mean ± SE of at least three independent experiments.

As determined, the inhibition of P-gp function by the most active novel derivatives, **6g** and **6i**, led to a significant intracellular increase of Dox in the resistant cell derivative. This intracellular availability allows this drug to reach the nucleus, where it induces its cytotoxic effect^49,50^. To demonstrate this, the effect of compounds **6g** and **6i** on the reversal of Dox resistance in K562/Dox was evaluated by co-administration of the drug with each derivative. Consistently, **6g** and **6i** at 1.56 µM managed to decrease the IC_50_ of Dox (Fig. 6), with FR values of 5.37 ± 1.36 (*p* < 0.05) and 2.69 ± 0.39 (*p* < 0.01), respectively. These results revealed that enhancing the intracellular drug concentration led to increased sensitivity to Dox. The Dox-reversal activity of compounds **6g** and **6i** was similar to (*p* > 0.05) and lower (*p* < 0.05) than that of verapamil, respectively. The latter was also applied at 1.56 µM (FR = 6.81 ± 1.20). Compound **6g** at 0**.**78 and at 0.39 µM (FR values of 3.98 ± 0.71 and 2.09 ± 0.34, respectively) achieved the same potency as that of verapamil (*p* > 0.05). The derivative **6g** was still active at 0.024 µM, with an FR value of 1.17 ± 0.05, showing a stronger activity (*p* < 0.01) than that of verapamil with a MEC of 0.098 µM and a FR value of 1.20 ± 0.05. Similarly, compound **6g** was 16 times more potent for restoring the cytotoxicity of Dox than the lead molecule **1**, which still chemosensitized K562/Dox at 0.39 µM. A dose-dependent activity was observed (b = − 36.87 *p* < 0.01; 95% CI − 55.16 to − 18.58) (Fig. 6). Compound **6i** at 0.78 and 0.39 µM displayed FR values of 1.63 ± 0.06 and 1.22 ± 0.10, respectively and, at the MEC value of 0.19 µM, the FR value corresponded to 1.39 ± 0.07 (Fig. 6), achieving the same reversal effect to verapamil (*p* > 0.05) at the same concentration. Like compound **6g**, this compound enhanced Dox cytotoxicity in a dose-dependent manner (b = − 31.88 p < 0.01; 95% CI − 46, 9 to − 16.87) and displayed nearly twice the chemosensitizer potency as that of compound **1**.

While compound **6g** at 1.56 µM produced a slight increase in Dox toxicity in the parental K562 cell line by some non-P-gp mediated mechanism, this was not observed when it was tested at 0.39 µM (FR of 1.10 ± 0.07; *p* > 0.05). Compound **6i** did not show a reversal effect on this cell line at 1.56 µM (Fig. 6), suggesting a selective effect on P-gp at all assayed concentrations.

### Structure–activity relationships

In this study, we aimed to compare two different types of bonding, ester and carbamate, between the betulin core and the pharmacophore counterpart. The ester derivatives **3a-3e** were found to be inactive opposite to compound **1**, but most of the carbamates, **6a**, **6d**, **6f**, **6g**, **6i** and **6k,** exhibited equal or better anti-P-gp activity than the lead compound **1**. On the other hand, the betulinic acid amide **4** was found to be 32-fold less active against P-gp than the corresponding betulin carbamate **6d** (MEC values of 25 μΜ and 0.78 μΜ, respectively, see Table 2).

**Figure 7:**
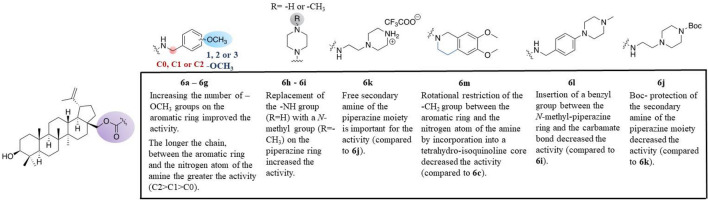
SAR at a glance for betulin esters and carbamates.

Another SAR objective was to study the influence on activity of the substituents on the aromatic ring of the pharmacophore counterpart. Previous studies demonstrated that an increased number of methoxy substituents favored anti-P-gp activity^23,33,36^. Our results confirmed these findings, since the P-gp inhibitory activity of betulin carbamates was favored with the increasing number of methoxy groups on the phenyl moiety of the conjugates, as evidenced by the MEC values of compounds **6b**, **6c** and **6d** (MEC = 50, 3.12 and 0.78 µM, respectively). The same was observed by comparing the MEC values of compounds **6e** (MEC = 50 µM), **6f** (MEC = 1.56 µM) and **6g** (MEC = 0.19 µM) with one, two and three –OCH_3_ groups, respectively. Further studies regarding the carbamates and, more specifically, the replacement of the methoxy-substituted aromatic ring by substituted piperazines and a tetrahydroquinoline (derivatives **6h-6m**), showed that less potent inhibitors were obtained, except for the carbamate **6i**, which was found to be the second most active derivative after compound **6g** (MEC = 0.39 and 0.19 µM, respectively). Likewise, when the length of the carbon chain connecting the C-28 of compound **1** and the aromatic ring increased, more potent derivatives were obtained. As observed in Table 2, compound **6a** (MEC = 1.56 µM) was less effective than compound **6d** and both showed weaker reversal activity than compound **6g**, all of them bearing three *O*-methyl groups. Similarly, compound **6c** was less effective than compound **6f**, both bearing two OCH_3_ substituents (MEC = 3.12 and 1.56 µM, respectively). The length of the chain did not alter the moderate effectiveness (MEC = 50 µM) of compounds **6b** and **6e** with only one *O*-methyl group. A summary of these findings is shown in Fig. 7.

Taking into consideration the SAR studies so far, the best candidates should contain the betulin core connected to a pharmacophore through a carbamate bonding. Regarding the structural elements in the pharmacophore counterpart, the increased number of -OMe groups on the phenyl ring and the length of the carbon chain folding both moieties of the molecules led to increased activity. These findings could be used to rationally guide the design and synthesis of new lead compounds with improved tumor P-gp inhibitory activity.

### Inhibitor binding and structural determinants by molecular modeling

The protocol used for molecular dynamics (MD) analysis has been recently tested thoroughly with other families of P-gp inhibitors^32,33^. In these studies, Dox was taken as the reference substrate and verapamil and tariquidar as known modulators. The antitumoral agent paclitaxel (Taxol) has been taken as the crystallographic reference, as it was co-crystallized in the experimental structure 6QEX, and re-docked to validate the docking protocol^33,51^. In the current work, the chemotherapeutic drug vincristine, co-crystallized into the structure 7A69^52^, was also included as a reference substrate.

**Figure 8:**
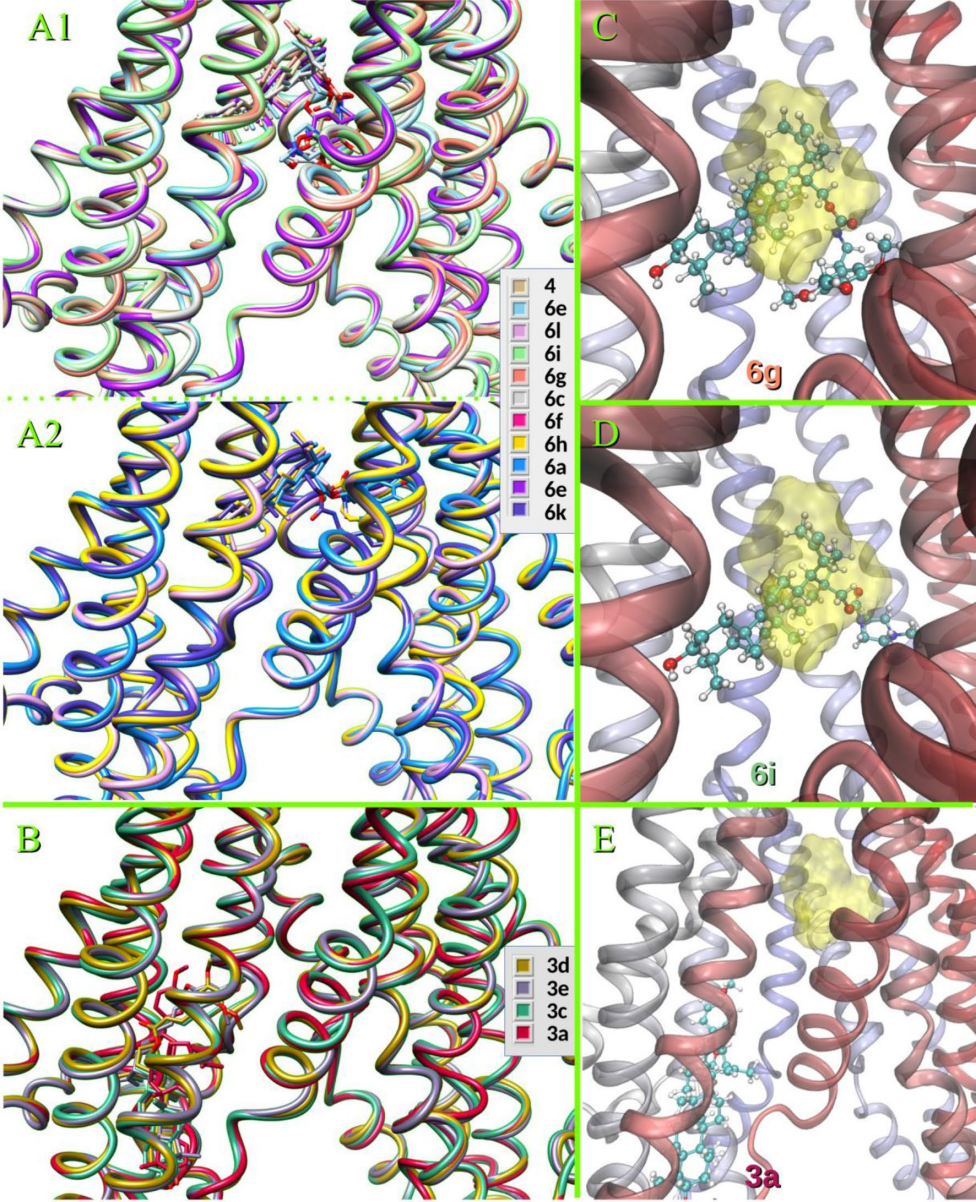
Superimposition of the aligned snapshots of the most populated clusters after a cluster analysis over the last 50 ns of each simulation for compounds binding to site I (**A**) and to site II (**B**). The poses of compounds representative of each pattern are superimposed on the one obtained for the simulation of Rho123 shown as a translucent yellow surface: (**C**) **6g**; (**D**) **6i** and (**E**) **3a**. The cartoon representation of the protein is colored in (**C**–**E**) according to the amino acid sequence, from red to blue.

To obtain the initial structures for the MD simulations, the docking protocol was used to scan inside the whole transmembrane region, making no assumptions about the localization of a particular binding site. The main binding sites were determined for the working substrate Rho123, for vincristine and compared to that previously obtained for Dox, verapamil and tariquidar^33^. Next up, target compounds were included, thus covering the whole range of experimental activities, from those completely inactive to those most powerful. A set of molecular MD simulations on these compounds was intended to reveal in detail the binding dynamics and the structural factors that determine their activity. As a first, gross observation, it should be noted that the compounds clearly distribute between two different sites as shown in Figs. 8A,B, which shows a superimposed and aligned representative snapshot of the most populated cluster of one trajectory for each compound. Amide **4** and carbamates of series **6** fell in the apex of the inverted “V” formed by TMH 4–6 from one homologous half and TMH 7 and 12 from the other; this pocket was labeled as site I. The compounds mentioned accommodate in two general modes with minor differences: (i) a distorted U, with the carbamate or the carbamate linked to the alkyl chain as the camber connecting the triterpene, and the aromatic moieties as the branches of the “U” (Fig. 8A1 and C with compound **6g** as example) or (ii) a V-shape with the piperazine or tetrahydroquinoline burying a bit deeper into the right side as observed in Fig. 8A2 (Fig. 8D showing compound **6i**, as representative). Both modes of binding shown in Fig. 8C and D fully overlap with that of vincristine and this, in turn, with that of Rho123 and what had been previously observed for Dox^33^ (see also Supplementary Material Figs. S1 and S2). In both cases, the triterpene moiety arranged practically in the same way into the hydrophobic cavity shaped by specific residues of TMH 4–7 and 12 (details below). On the other hand, compounds from series **3** went to a different site, hereinafter referred as site II, involving different regions of TMH 4, 5 and 12 and TMH 8 and 11 (Fig. 8B and E featuring **3a** as example).

**Figure 9:**
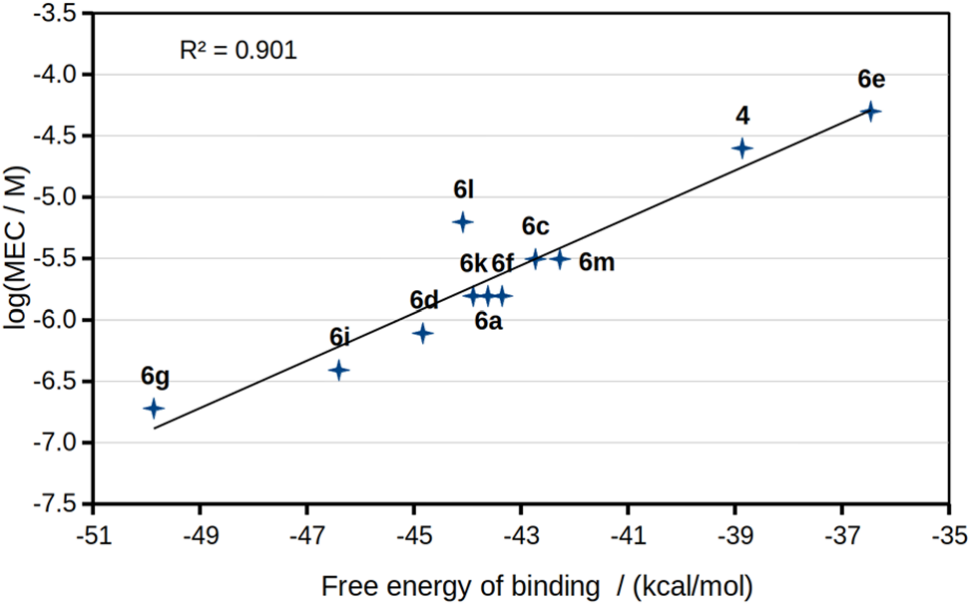
Correlation between the logarithms of the minimum effective concentrations (MEC) (Table 2) and the Δ*G°*_*b*_, calculated from the MD simulations*.*

A summary of the calculated free energies of binding (Δ*G°*_*b*_) from these simulations is shown in Table 3. The compounds interacting with the residues of site I showed a good correlation with the experimental MEC values for the Rho123 intracellular accumulation assay, as shown in Fig. 9. In sharp contrast, compounds of series **3**, which interact with site II, did not modulate the activity of P-gp, regardless of their binding energies. This preference for what we describe as site I is remarkable, besides the evident overlap with Rho123 and even with the bulkier chemotherapeutics substrates, Dox and vincristine (Supplementary Material Fig. S3). Indeed, these findings reinforce the relevance of the residues involved in binding site I, as was previously discussed in studies from our group^32,33^ and in the literature cited therein. The results obtained highlight not only the identity of these residues, but also the mandatory requisite for tightly contacting both halves of the transporter at once, especially TMH 6 and 12, which connect the TMHs and the NBDs.

**Table 3:** Free binding energies from the MMPBSA analyses of the MD simulations.

Substrates	Δ*G°*_*b*_/(kcal/mol)	MEC (µM)
Rhodamine123	− 23.5	
Vincristine	− 41.3	
Doxorubicin	− 24.2	
Verapamil	− 35.7^a^	0.39
Tariquidar	− 38.3^a^	0.78
Subject compounds in site I		
** 6g**	− 49.9	0.19
** 6i**	− 46.4	0.39
** 6d**	− 44.8	0.78
** 6a**	− 43.6	1.56
** 6f**	− 43.4	1.56
** 6k**	− 43.9	1.56
** 6c**	− 42.7	3.12
** 6m**	− 42.3	3.12
** 6l**	− 44.1	6.25
** 4**	− 38.9	25.0
** 6e**	− 36.5	50.0
Subject compounds in site II		
** 3c**	− 50.2	> 50
** 3e**	− 52.3	> 50
** 3a**	− 40.5	> 50
** 3d**	− 36.0	> 50

^a^Δ*G°*_*b*_ calculated in Laiolo et al.^33^.

**Figure 10:**
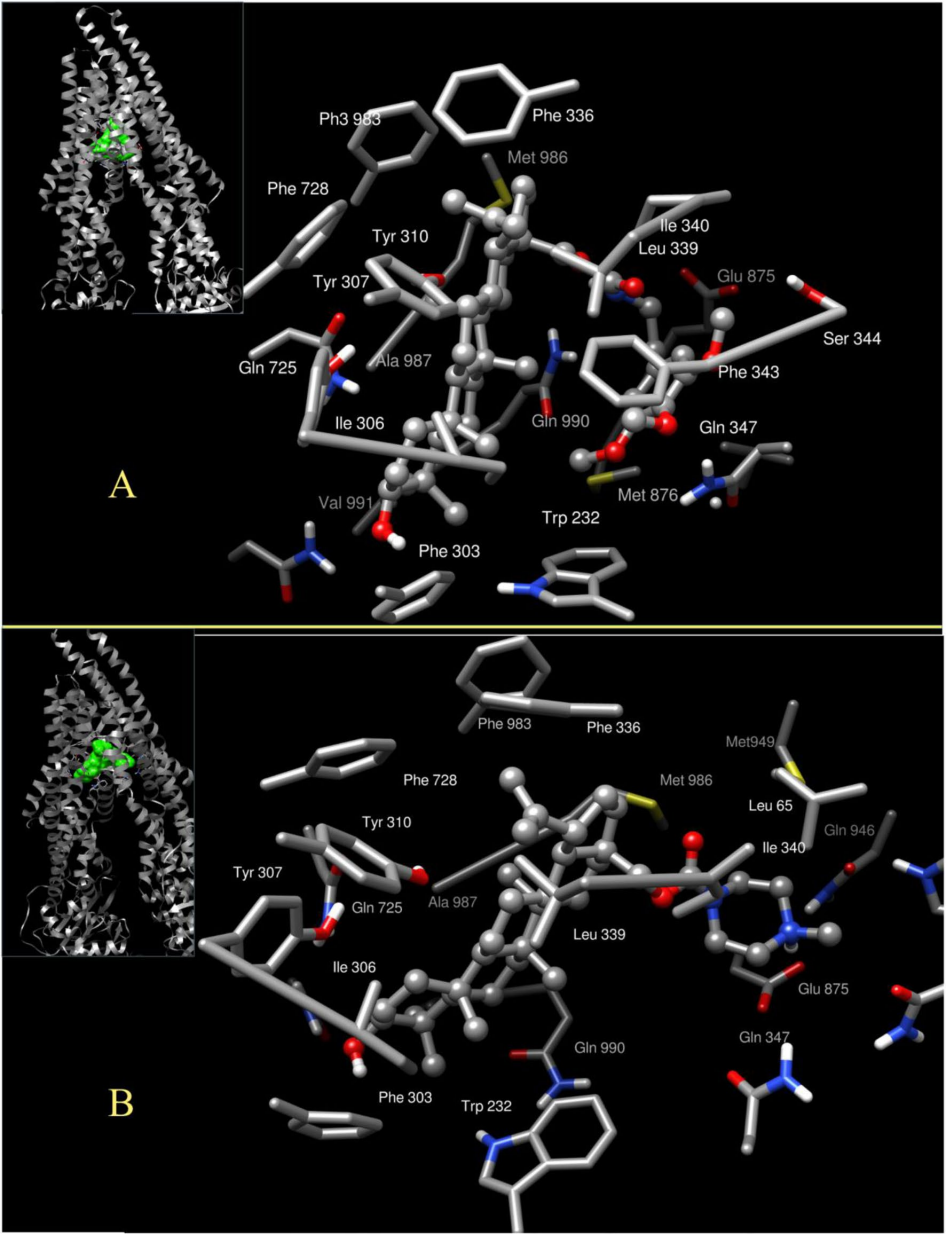
Snapshot of the most populated cluster of one trajectory showing the contacts for compound **6g** (**A**) and compound **6i** (**B**). Nonpolar hydrogens and backbone atoms are not shown.

A closer look at the binding modes of the most powerful inhibitors **6g** and **6i** is shown in two representative snapshots of the most populated cluster for each trajectory in Fig. 10, which can be taken as illustrative for the two variants of binding in this site for the amide- or carbamate-alkyl-bridged series. The triterpene moiety fits in the cavity shaped by the hydrophobic residues Trp232 from TMH 4, Phe303, Ile306, Tyr307 and Tyr310 from TMH 5, Phe336, Leu339, Ile340 and Phe343 from TMH 6, Phe728 from TMH 7, Phe983, Met986, Ala987, the alkyl chain of Gln990 and Val991 from TMH 12 (images of **6g** and **6i** on the hydrophobicity-colored surface available in Supplementary Material, Fig. S4). The interaction with the 3-OH of the steroid moiety and the π system of Phe303 is present in all cases, as an anchor in the limit of the hydrophobic pocket; to the left (Fig. 10), some polar residues appear, Gln725 and Gln828. The methoxy-substituted ring of **6g** (and similarly, though with weaker interactions for **6a-f**) interacts with Trp232, Gln347 (hydrophobic or H-bound, depending upon the substitution of their phenyl rings), Phe343 and Ser 344. The aliphatic or carbamate-alkyl “bridges” of compound **4** and of series **6** contact mainly Ile340, Met876, Met986 and Glu990 and, more importantly, contribute to the flexibility that enables the twisting shape (Fig. 10A,B). The main difference between **6g** and **6i** is the strong electrostatic contact of the latter with Glu875 and the contacts with Leu65 and Gln946, as observed in the right side of Fig. 10.

These features are absent in compounds **3a**, **3c**, **3d** and **3a**, which were found to be not flexible enough for arranging in this mode and therefore fell to site II. The ester function itself does not appear as a drawback, as the NH of the amide present in the carbamates **6** or in the amine of **4** is not generally involved in strong H-bond interactions. The absence of sp^3^-hybridized atoms between the carbonyl and the aromatic moiety seems to favor more extended conformations, as previously observed for quinolinone-pyrimidine hybrids, for which the conjugated carbonyl-vinyl bridge conferred rigidity and decreased or prevented the activity of these species^33^.

**Figure 11:**
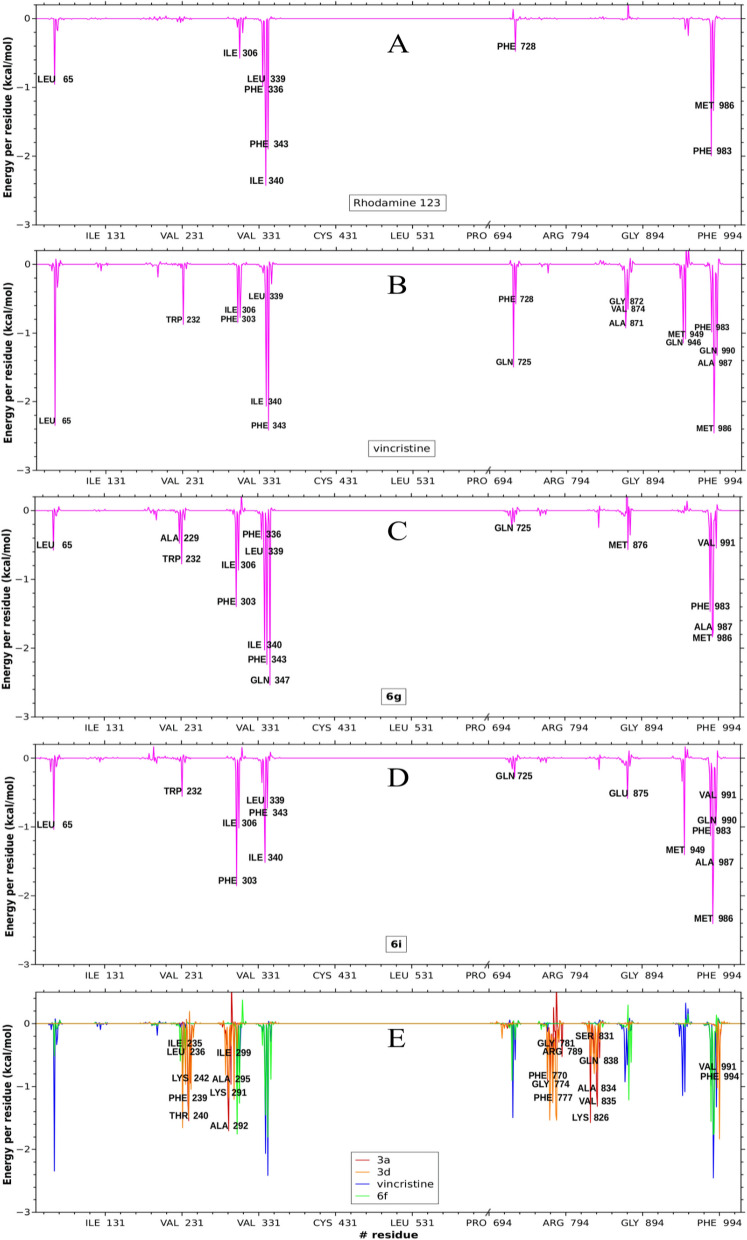
Free energy decomposition in terms of per residue contribution obtained for rhodamine 123 (**A**); vincristine (**B**); compound **6g** (**C**); and compound **6i** (**D**). (**E**) The profiles obtained for vincristine (blue) and compound **6f** (green) are superimposed on those calculated for **3a** (red) and **3d** (orange). The labels correspond to the residues of the latter.

Besides the above features described in Fig. 10, deeper analyses were obtained by energy decomposition to quantify the contribution of each residue averaged over the time of the simulation. Figure 11A–D compares the patterns obtained for Rho123, vincristine, **6g**, and **6i**. Both target compounds had prominent peaks (denoting persistent interactions) for the same residues as Rho123 and vincristine. These peaks also match those obtained for Dox and tariquidar, for which the free energy contribution per residue is reproduced from previous calculations^33^ (Supplementary Material Fig. S5).

The patterns obtained for compounds **3a** and **3c** are superimposed in Fig. 11E on those of vincristine and compound **6f** (less powerful than compound **6g** but still overlapping the peaks of the substrates). As can be observed, almost all peaks of both derivatives, **3a** and **3c**, corresponded to different residues than that observed for the substrates or the series **6** partners (for example, note the lack of any interaction with TMH 6).

### Selective toxicity of compounds 6g and 6i over mammalian cells

Derivative **6g** did not reveal cytotoxicity on PBMC by means of the MTT assay at 50 µM, while the same concentration of compound **6i** slightly affected cell viability (64%). The IC_50_ value of 26.37 ± 2.88 µM suggests that the latter molecule could not be considered as cytotoxic, based on the 10 µM value established by the US National Cancer Institute^41^. Neither compound induced erythrocyte hemolysis at the maximum tested concentration of 50 µM.

## Conclusions

In our ongoing search for novel P-gp inhibitors, a panel of new betulin derivatives was designed on the basis of the structural requirements previously determined as important for P-gp interaction. Among the series of derivatives obtained, compounds **6g** and **6i** proved to be the most potent and efficacious for blocking the P-gp-mediated efflux of Rho123. Further study of the pharmacological profile of both entities revealed that they increased the intracellular accumulation of Dox, rendering K562/Dox cells sensitive to its cytotoxic effect. The large amount of data obtained herein and the negligible cytotoxicity of compounds **6g** and **6i** lay the groundwork for a second step involving further in vitro studies and in vivo studies.

The activity was positively potentiated by the number of aromatic ring methoxy substituents and the length and flexibility of the chain linking both parts of the molecule, which provided an appropriate fit into a specific region of the large TMD. Such a preference is not trivial for a multispecific and substrate-promiscuous transporter like P-gp and was already observed for other families of potential inhibitors^32,33^. This mechanism of inhibition certainly deserves further investigation. However, at this point it reveals very useful clues for the rational design of new inhibitors by finding: (I) molecular shapes designed to arrange within the hydrophobic cavity described as part of site I (compound **1** scaffold appeared to do it very well as previously observed^32^; (II) appropriate linkers and methoxylated aromatics or piperazine derivatives allowing tight contact with TMH 6 and 12 simultaneously.

The data obtained establish the basis for developing compounds which, administered in combination with chemotherapeutic agents transported by P-gp, could restore the efficacy of these in P-gp/MDR cancer cells.

## Materials and methods

### Chemistry

All solvents were dried and purified prior to use according to standard procedures. When required, reactions were performed under inert atmosphere (Ar) in preflamed glassware. Anhydrous Na_2_SO_4_ was used for drying solutions, and the solvents were then routinely removed at ca. 40 °C under reduced pressure using a rotary vacuum evaporator. All reagents employed in the present work were commercially available and used without further purification. Among all the amines employed in the synthesis of the betulin carbamates, **6a-m** and tert-butyl-4-(2-aminoethyl) piperazine-1-carboxylate, used for the synthesis of **6j,** were prepared according to a previously published protocol^53^. 2-(3,4-dimethoxyphenyl)ethan-1-amine and 2-(3,4,5-trimethoxyphenyl)ethan-1-amine employed in the synthesis of **6f** and **6g**, respectively, were synthesized and characterized according to the procedures described in Supplementary Material. Flash column chromatography (FCC) was performed on silica gel (70–230 and 230–400 mesh, Merck, Germany) and analytical thin layer chromatography (TLC) on silica 60gel-F254 precoated aluminum foils (0.2 mm film, Merck, Germany). Spots on the TLC plates were visualized with UV light at 254 nm and using anisaldehyde solution. ^1^H NMR spectra were recorded in CDCl_3_ at 600.13 MHz and ^13^C spectra at 150.9 MHz on a Bruker AVANCEIII HD spectrometer. Chemical shifts (*δ*) are indicated in parts per million downfield from TMS. Coupling constants (*J*) are reported in Hertz. HR mass spectra were performed using an ESI-LTQ-ORBITRAP XL unit (Thermo Scientific, Bremen, Germany). The Orbitrap Unit was operated in positive mode, with a spray voltage of 3.2 kV, while the sheath gas flow rate and auxiliary gas flow rate were adjusted to 12 and 2 arbitrary units, respectively. The capillary voltage and the tube lens voltage were set to 10 and 110 V, respectively. The scan ranged from *m*/*z* 150 up to 2000. The purity of the tested compounds was determined by reversed phase high performance liquid chromatography (HPLC) (Jasco LC-NetII/ADC series) equipped with a Phenomenex Gemini-NX RP-C18 150 × 4.6 mm, 5 μm particle size column and a MD-4010 PDA Detector and monitored at 254 and 280 nm. Linear gradients of A and B were used where A = 0.1% TFA in H_2_O and B = 0.1% TFA in CH_3_CN, at a flow rate of 0.8 mL/min. The purity of the tested compounds, which corresponded to > 95% (see the Supplementary Material), was evaluated as a percentage ratio between the areas of the main peak and of possible impurities.

### General procedure for the synthesis

#### General procedure for the synthesis of betulin esters 3a-e

To a stirring solution of compound **1** in dry DCM/THF (3:2 v/v, 0.05 M), under Ar atmosphere, the aromatic acid (1.5 eq) and 4-dimethylaminopyridine (1.75 eq) were added, and the suspension was cooled to 0 °C. Subsequently DCC (1.75 eq) was added, and the reaction mixture was stirred for 5 min at 0 °C and then at ambient temperature overnight. Upon consumption of the starting material, dicyclohexylurea (DCU) was filtered under vacuum and washed in the filtration funnel with ethyl acetate (AcOEt). After evaporation of the solvent to dryness under reduced pressure, the residue was subjected to FCC, affording the pure betulin esters **3a-e** as colorless oils.

**3a**: compound **1** was reacted with 4-methoxybenzoic acid according to the general procedure. Yield = 98%; Colorless oil; R_f_ (PhMe/EtOAc 95:5) = 0.27; ^1^H NMR (600 MHz, CDCl_3_) *δ* 8.01 (br s, 1 H), 7.99 (br s, 1 H), 6.93 (br s, 1 H), 6.91 (br s, 1 H), 4.72 (d,* J* = 2.5 Hz, 1 H), 4.60 (t, *J* = 1.4 Hz, 1 H), 4.48 (dd, *J* = 11.1, 2.1 Hz, 1 H), 4.06 (dd, *J* = 11.0, 1.4 Hz, 1 H), 3.86 (s, 3 H), 3.18 (dd, *J* = 11.5, 4.8 Hz, 1 H), 2.52 (td, *J* = 11.3, 5.9 Hz, 1 H), 2.06–1.95 (m, 2 H), 1.91 (ddd, *J* = 12.4, 8.4, 1.2 Hz, 1 H), 1.80–1.73 (m, 2 H), 1.70 (s, 3 H), 1.68–1.21 (m, 16 H), 1.18–1.08 (m, 2 H), 1.06 (s, 3 H), 1.0 (s, 3 H), 0.97 (s, 3 H), 0.91 (td, *J* = *1*4.1, 4.4 Hz, 1 H), 0.83 (s, 3 H), 0.76 (s, 3 H), 0.68 (d, *J* = 9.5 Hz,1 H); ^13^C NMR (151 MHz, CDCl_3_) *δ* 166.7, 163.3, 150.2, 131.5, 122.9, 113.6, 109.8, 79.0, 62.9, 55.4, 55.3, 50.4, 48.9, 47.8, 46.7, 42.8, 40.9, 38.9, 38.7, 37.6, 37.2, 34.7, 34.2, 30.0, 29.7, 28.0, 27.4, 27.2, 25.2, 20.8, 19.2, 18.3, 16.1, 16.1, 15.3, 14.8; HR-ESI (*m*/*z*) [2 M + H^+^] calculated for C_38_H_57_O_4_ 1153.8438. Found: 1153.8428; HPLC analysis: peak area 99.5%, t_R_ = 14.30 min, gradient: 90% (3 min to 90%) to 95% B over 5 min and from 95 to 100% B over 10 min.

**3b**: compound **1** was reacted with 3,4,5-trimethoxybenzoic acid according to the general procedure. Yield = 71%; Colorless oil; R_f_ (PhMe/EtOAc 9:1) = 0.24; ^1^H NMR (600 MHz, CDCl_3_) *δ* 7.31 (s, 2 H), 4.72 (d, *J* = 2.6 Hz, 1 H), 4.61 (t,* J* = 1.5 Hz, 1 H), 4.53 (dd, *J* = 11.1, 1.9 Hz, 1 H), 4.08 (d, *J* = 11.1 Hz, 1 H), 3.91 (s, 9 H), 3.19 (dd, *J* = 11.6, 4.8 Hz, 1 H), 2.54 (td, *J* = 11.1, 5.8 Hz, 1 H), 2.08–2.00 (m, 1 H), 1.95–1.86 (m, 2 H), 1.81–1.72 (m, 2 H), 1.71 (s, 3 H), 1.69–1.65 (m, 2 H), 1.64–1.14 (m, 16 H), 1.07 (s, 3 H), 1.00 (s, 3 H), 0.97 (s, 3 H), 0.91 (td, *J* = 13.4, 4.4 Hz, 1 H), 0.83 (s, 3 H), 0.76 (s, 3 H), 0.69 (d, *J* = 9.4 Hz, 1 H); ^13^C NMR (151 MHz, CDCl_3_) *δ* 166.5, 152.9, 150.1, 125.5, 110.0, 106.8, 79.0, 63.5, 60.9, 56.3, 55.3, 50.4, 48.8, 47.8, 46.8, 42.8, 40.9, 38.9, 38.7, 37.7, 37.2, 34.8, 34.2, 30.1, 29.7, 28.0, 27.4, 27.2, 25.2, 20.8, 19.1, 18.3, 16.1, 16.0, 15.3, 14.8; HR-ESI (*m*/*z*) [2 M + H^+^] calculated for C_40_H_61_O_6_ 1273.8860. Found: 1273.8845; HPLC analysis: peak area 99.3%, t_R_ = 12.90 min, gradient: 90% (3 min to 90%) to 95% B over 5 min and from 95 to 100% B over 10 min.

**3c**: compound **1** was reacted with *trans*-2,4-dimethoxycinnamic acid according to the general procedure. Yield = 95%; Colorless oil; R_f_ (PhMe/EtOAc 9:1) = 0.4; ^1^H NMR (600 MHz, CDCl_3_) *δ* 7.92 (d, *J* = 16.2 Hz, 1 H), 7.45 (d, *J* = 8.6 Hz, 1 H), 6.50 (dd, *J* = 8.5, 2.4 Hz, 1 H), 6.45–6.42 (m, 2 H), 4.71 (d, *J* = 2.5 Hz, 1 H), 4.60 (t,* J* = 1.5 Hz, 1 H), 4.39 (dd, *J* = 11.1, 2.0 Hz, 1 H), 3.97 (d, *J* = 11.1 Hz, 1 H), 3.87 (s, 3 H), 3.84 (s, 3 H), 3.18 (dd, *J* = 11.4, 4.8 Hz, 1 H), 2.50 (td, *J* = 11.2, 5.9 Hz, 1 H), 2.05–1.98 (m, 1 H), 1.93 (m, 1 H), 1.89–1.84 (m, 1 H), 1.79–1.71 (m, 2 H), 1.69 (s, 3 H), 1.68–1.39 (m, 13 H), 1.30–1.23 (m, 3 H), 1.22–1.08 (m, 2 H), 1.06 (s, 3 H), 0.99 (s, 3 H), 0.97 (s, 3 H), 0.90 (td, *J* = 12.6, 4.0 Hz, 1 H), 0.83 (s, 3 H), 0.76 (s, 3 H), 0.68 (d, *J* = 9.6 Hz, 1 H); ^13^C NMR (151 MHz, CDCl_3_) *δ* 168.4, 162.7, 159.8, 150.3, 140.0, 130.4, 116.7, 116.1, 109.8, 105.2, 98.4, 79.0, 62.5, 55.4, 55.3, 50.4, 48.9, 47.7, 46.6, 42.7, 40.9, 38.9, 38.7, 37.6, 37.2, 34.7, 34.2, 29.9, 29.7, 28.0, 27.4, 27.2, 25.2, 20.8, 19.2, 18.3, 16.1, 16.0, 15.3, 14.8; HR-ESI (*m*/*z*) [M + H^+^] calculated for C_41_H_61_O_5_ 633.4519. Found: 633.4516; HPLC analysis: peak area 94.8%, t_R_ = 13.20 min, gradient: 90% (3 min to 90%) to 95% B over 5 min and from 95 to 100% B over 10 min.

**3d**: compound **1** was reacted with 3,4,5-trimethoxycinnamic acid according to the general procedure. Yield = 64%; Colorless oil; R_f_ (PhMe/EtOAc 9:1) = 0.29; ^1^H NMR (600 MHz, CDCl_3_) *δ* 7.59 (d, *J* = 15.6 Hz, 1 H), 6.75 (s, 2 H), 6.36 (d, *J* = 15.6 Hz, 1 H), 4.71 (d, *J* = 2.5 Hz, 1 H), 4.60 (t,* J* = 1.5 Hz, 1 H), 4.40 (dd, *J* = 11.4, 2.0 Hz, 1 H), 4.00 (d, *J* = 11.4 Hz, 1 H), 3.89 (s, 6 H), 3.88 (s, 3 H), 3.18 (dd, *J* = 11.4, 4.8 Hz, 1 H), 2.50 (td, *J* = 11.1, 5.6 Hz, 1 H), 2.05–1.97 (m, 1 H), 1.96–1.91 (m, 1 H), 1.89–1.83 (m, 1 H), 1.78–1.71 (m, 2 H), 1.70 (s, 3 H), 1.65–1.24 (m, 18 H), 1.06 (s, 3 H), 1.00 (s, 3 H), 0.97 (s, 3 H), 0.91 (td, *J* = 13.1, 4.2 Hz, 1 H), 0.83 (s, 3 H), 0.76 (s, 3 H), 0.69 (d, *J* = 11.2 Hz, 1 H); ^13^C NMR (151 MHz, CDCl_3_) *δ* 167.4, 153.4, 150.1, 144.6, 140.1, 129.9, 129.0, 128.2, 117.5, 109.9, 105.2, 79.0, 62.9, 61.0, 56.2, 55.3, 50.4, 48.9, 47.7, 46.6, 42.7, 40.9, 38.9, 38.7, 37.6, 37.2, 34.7, 34.2, 29.9, 29.6, 28.0, 27.4, 27.1, 25.2, 20.8, 19.1, 18.3, 16.1, 16.0, 15.3, 15.2, 14.8; HR-ESI (*m*/*z*) [M + H^+^] calculated for C_42_H_63_O_6_ 663.4624. Found: 633.4570; HPLC analysis: peak area 99.1%, t_R_ = 12.60 min, gradient: 90% (3 min to 90%) to 95% B over 5 min and from 95 to 100% B over 10 min.

**3e**: compound **1** was reacted with 2,4,5-trimethoxycinnamic acid according to the general procedure. Yield = 47%; Colorless oil; R_f_ (PhMe/EtOAc 9:1) = 0.16; ^1^H NMR (600 MHz, CDCl_3_) *δ* 7.98 (d, *J* = 16.2 Hz, 1 H), 7.02 (s, 1 H), 6.50 (s, 1 H), 6.38 (d, *J* = 16.2 Hz, 1 H), 4.71–4.70 (m, 1 H), 4.60 (t,* J* = 1.6 Hz, 1 H), 4.40 (dd, *J* = 11.2, 1.9 Hz, 1 H), 3.98 (d, *J* = 11.4 Hz, 1 H), 3.93 (s, 3 H), 3.87 (s, 3 H), 3.86 (s, 3 H), 3.19 (dd, *J* = 11.4, 4.8 Hz, 1 H), 2.51 (td, *J* = 11.1, 5.7 Hz, 1 H), 2.07–1.98 (m, 1 H), 1.96–1.91 (m, 1 H), 1.89–1.85 (m, 1 H), 1.80–1.71 (m, 2 H), 1.70 (s, 3 H), 1.66–1.11 (m, 18 H), 1.06 (s, 3 H), 0.99 (s, 3 H), 0.97 (s, 3 H), 0.91–0.86 (m, 1 H), 0.83 (s, 3 H), 0.76 (s, 3 H), 0.68 (d, *J* = 9.5 Hz, 1 H); ^13^C NMR (151 MHz, CDCl_3_) *δ* 168.2, 153.9, 152.1, 150.3, 143.3, 139.5, 129.0, 125.3, 115.8, 115.1, 110.9, 109.8, 96.9, 79.0, 62.5, 56.5, 56.0, 55.3, 50.4, 48.9, 47.7, 46.6, 42.7, 40.9, 38.9, 38.7, 37.6, 37.2, 34.7, 34.2, 29.9, 29.7, 28.0, 27.4, 27.2, 25.2, 20.8, 19.1, 18.3, 16.1, 16.0, 15.3, 14.8; HR-ESI (*m*/*z*) [M + H^+^] calculated for C_42_H_63_O_6_ 663.4624. Found: 633.4619; HPLC analysis: peak area 98.1%, t_R_ = 11.33 min, gradient: 90% (3 min to 90%) to 95% B over 5 min and from 95 to 100% B over 10 min.

#### General procedure for the synthesis of amide 4

To an ice-cold stirring solution of compound **2** (20 mg, 0.044 mmol) in dry THF, HOBt (8 mg, 0.06 mmol) and DCC (10 mg, 0.05 mmol) were added, and the solution was stirred at room temperature for 15 h. After completion of the reaction, the mixture was diluted in 1,2-dichloroethane (DCE) and washed sequentially with 5% aqueous ice-cold NaHCO_3_, water and brine. Then it was dried over anhydrous Na_2_SO_4_, filtered and concentrated to dryness, to provide the corresponding active ester as a white solid (22 mg, 88% yield); ^1^H NMR (600 MHz, CDCl_3_) *δ* 8.08 (d, *J* = 8.4 Hz, 1 H), 7.56 (ddd, *J* = 7.2, 1.2, 0.6 Hz, 1 H), 7.43 (ddd, *J* = 7.2, 1.2, 0.6 Hz, 1 H), 7.36 (d, *J* = 8.2 Hz, 1 H), 4.73 (d, *J* = 2.3 Hz, 1 H), 4.63 (t, *J* = 1.7 Hz, 1 H), 4.17 (d, *J* = 8.4 Hz, 1 H), 3.51–3.43 (m, 1 H), 3.21–3.15 (m, 2 H), 2.95 (td, *J* = 11.2, 5.0 Hz, 1 H), 2.65–2.61 (m, 1 H), 2.41 (dd, *J* = 13.0, 8.1 Hz, 1 H), 2.21 (td, *J* = 12.6, 3.7 Hz, 1 H), 2.12–2.04 (m, 1 H), 1.71 (s, 3 H), 1.61–1.51 (m, 7 H), 1.47–1.40 (m, 4 H), 1.35–1.25 (m, 6 H), 1.18–1.24 (m, 2 H), 1.08–1.10 (m, 1 H), 1.04 (s, 3 H), 0.98 (s, 3 H), 0.97 (s, 3 H), 0.90 (td,* J* = 13.6, 4.1 Hz, 1 H), 0.81 (s, 3 H), 0.76 (s, 3 H), 0.69–0.72 (m, 1 H); ^13^C NMR (151 MHz, CDCl_3_) *δ* 171.8, 157.1, 149.2, 143.6, 128.9, 128.7, 124.7, 120.6, 110.4, 107.9, 78.9, 57.0, 55.8, 55.4, 50.5, 49.9, 49.1, 46.6, 42.5, 40.8, 38.9, 38.7, 38.45, 37.2, 36.8, 34.9, 34.4, 33.9, 31.4, 30.3, 30.1, 28.0, 27.4, 25.6, 25.4, 24.9, 20.8, 19.4, 18.3, 16.1, 15.4, 14.8.

To a solution of the above active ester (22 mg, 0.04 mmol) in dry THF (50 μL) under Ar atmosphere and stirring at 0 °C, 3,4,5-trimethoxybenzylamine (12 μL, 0.067 mmol) was added and the reaction mixture was stirred for another 16 h at ambient temperature. After completion of the reaction, the mixture was diluted in ethyl acetate and washed sequentially with 5% aqueous ice-cold citric acid, water, and brine. The organic layer was dried over anhydrous Na_2_SO_4_, filtered, and concentrated to dryness. The residue obtained was subjected to FCC using PhMe/EtOAc 9:1 as eluent, affording the corresponding amide **4** in 50% yield; White solid; m.p.: 99.7–100.5 °C; R_f_ (PhMe/EtOAc 95:5) = 0.08; ^1^H NMR (600 MHz, CDCl_3_) *δ* 6.49 (s, 2 H), 5.94–5.89 (m, 1 H), 4.74 (d, *J* = 2.9 Hz, 1 H), 4.60 (t, *J* = 2.2 Hz, 1 H), 4.40 (dd, *J* = 14.4, 5.8 Hz, 1 H), 4.34 (dd,* J* = 14.8 Hz, 5.8 Hz, 1 H), 3.84 (d, *J* = 1.2 Hz, 6 H), 3.83 (d, *J* = 0.6 Hz, 3 H), 3.19–3.12 (m, 2 H), 2.52 (td,* J* = 13.0, 3.7 Hz, 1 H), 1.97–1.90 (m, 2 H), 1.78 (dd, *J* = 12.3, 7.6 Hz, 1 H), 1.73–1.71 (m, 1 H), 1.69 (s, 3 H), 1.66–1.64 (m, 1H), 1.62–1.52 (m, 5 H), 1.58–1.33 (m, 9 H), 1.30–1.23 (m, 3 H), 1.14 (dt, *J* = 13.3, 3.0 Hz, 1 H), 0.97 (s, 3 H), 0.96 (s, 3 H), 0.95–0.92 (m, 1 H), 0.90 (s, 3 H), 0.81 (s, 3 H), 0.75 (s, 3 H), 0.68–0.65 (m, 1 H); ^13^C NMR (151 MHz, CDCl_3_) *δ* 175.9, 153.4, 150.8, 137.1, 135.0, 129.0, 128.2, 109.5, 104.6, 79.0, 60.8, 56.0, 55.7, 55.4, 50.6, 50.1, 46.7, 43.3, 42.5, 40.7, 38.8, 38.7, 38.4, 37.6, 37.2, 34.4, 33.7, 30.9, 29.4, 28.0, 27.4, 25.6, 24.9, 20.9, 19.5, 18.3, 16.2, 16.1, 15.3, 14.6; HR-ESI (*m*/*z*) [M + H^+^] calculated for C_40_H_61_NO_5_ 635.4550. Found: 635.4573; HPLC analysis: analysis: peak area 98.9%, t_R_ = 6.02 min, gradient: 90% (3 min to 90%) to 95% B over 5 min and from 95 to 100% B over 10 min.

### General procedure for the synthesis of betulin p-nitrophenylcarbonate ester 5

To a stirring solution of compound **1** (200 mg, 0.45 mmol) in dry THF (10 mL), under Ar atmosphere, pyridine (36 μL, 0.45 mmol) was added, and the mixture was cooled to 0 °C. *p*-nitrophenylchloroformate (94.3 mg, 0.47 mmol) was added in 3 portions and the reaction mixture was stirred for 24 h at ambient temperature. Consequently, the mixture was concentrated under reduced pressure to dryness. The solid residue obtained was diluted in DCM and washed sequentially with 5% aqueous ice-cold citric acid, water, and brine, dried over anhydrous Na_2_SO_4_, filtered and concentrated to dryness. The desired product was afforded as a white foam (227 mg) after FCC purification using PhMe/EtOAc 95:5 as eluent. Yield = 83%; White foam; R_f_ (PhMe/EtOAc 95:5) = 0.14; ^1^H NMR (600 MHz, CDCl_3_) *δ* 8.29 (s, 1 H), 8.28 (s, 1 H), 7.41 (s, 1 H), 7.39 (s, 1 H), 4.71 (br s, 1 H), 4.61 (t,* J* = 1.7 Hz, 1 H), 4.51 (dd, *J* = 10.8 Hz, 2.0 Hz, 1 H), 4.08 (d, *J* = 10.7 Hz, 1 H), 3.19 (dd, *J* = 11.4, 4.8 Hz, 1 H), 2.45 (td, *J* = 10.8, 5.4 Hz, 1 H), 2.03 (dtd, *J* = 14.2, 10.5, 4.9 Hz, 1 H), 1.95–1.84 (m, 2 H), 1.75 (td, *J* = 13.9, 4.7 Hz, 1 H), 1.70 (s, 3 H), 1.68–1.08 (m, 19 H), 1.05 (s, 3 H), 1.00 (s, 3 H), 0.97 (s, 3 H), 0.91 (td, *J* = 12.5, 3.7 Hz, 1 H), 0.83 (s, 3 H), 0.76 (s, 3 H), 0.69 (d, *J* = 11.2 Hz, 1 H);. ^13^C NMR (151 MHz, CDCl_3_) *δ* 155.6, 153.0, 149.7, 145.3, 125.3, 121.8, 78.9, 68.3, 55.3, 50.3, 48.8, 47.7, 46.7, 42.7, 40.9, 38.9, 38.7, 37.7, 37.1, 34.4, 34.2, 29.6, 29.5, 28.0, 27.4, 27.0, 25.2, 20.7, 18.3, 16.1, 16.0, 15.3, 14.8.

#### General procedure for the synthesis of betulin carbamates 6a-m

To a stirring solution of carbonate ester **5** (0.16 mmol) in dry THF (0.2 M), under Ar atmosphere, the corresponding amine (appropriate equivalents for each case, see below) and DIPEA (1 eq) were added, and the reaction mixture was stirred at ambient temperature or heated at 50–60 °C. After completion of the reaction the mixture was diluted in EtOAc and washed sequentially with 5% aqueous ice-cold citric acid, water, and brine, dried over anhydrous Na_2_SO_4_, filtered, and concentrated to dryness. The residue thus obtained was purified by FCC to provide, upon solvent removal, the corresponding carbamates **6a-m**.

**6a**: compound **6a** was prepared using 3 equivalents of 3,4,5-trimethoxyaniline and overnight heating. Yield = 45%; Colorless oil; R_f_ (PhMe/EtOAc 9:1) = 0.11; ^1^H NMR (600 MHz, CDCl_3_) *δ* 6.69 (s, 1 H), 6.58 (s, 1 H), 4.70 (d,* J* = 2.5 Hz, 1 H), 4.60 (t, *J* = 1.7 Hz, 1 H), 4.34 (d, *J* = 10.7 Hz, 1 H), 4.07 (t, *J* = 6.8 Hz, 1 H), 3.98 (d, *J* = 10.1 Hz, 1 H), 3.85 (s, 6 H), 3.81 (s, 3 H), 3.19 (dd, *J* = 11.5, 4.7 Hz, 1 H), 2.47 (td, *J* = 11.1, 5.7 Hz, 1 H), 2.02–1.98 (m, 1 H), 1.91–1.71 (m, 4 H), 1.70 (s, 3 H), 1.66–1.51 (m, 5 H), 1.45–1.37 (m, 6 H), 1.30–1.25 (m, 2 H), 1.22 (dd, *J* = 12.8, 4.7 Hz, 1 H), 1.12–1.07 (m, 2 H), 1.06 (s, 3 H), 1.04–1.00 (m, 1 H), 0.99 (s, 3 H), 0.98 (s, 3 H), 0.95–0.92 (m, 1 H), 0.91–0.88 (m, 1 H), 0.83 (s, 3 H), 0.77 (s, 3 H), 0.69 (d, J = 9.5 Hz, 1 H); ^13^C NMR (151 MHz, CDCl_3_) *δ* 153.4, 150.1, 134.0, 125.3, 121.8, 109.9, 79.0, 64.4, 61.0, 56.1, 55.3, 50.4, 48.8, 47.7, 46.5, 42.7, 40.9, 38.9, 38.7, 37.6, 37.1, 34.5, 34.2, 29.8, 29.6, 28.0, 27.4, 27.1, 25.2, 20.8, 18.3, 16.1, 15.3, 14.8, 13.7; HR-ESI (*m*/*z*) [M + H^+^] calculated for C_40_H_62_NO_6_ 652.4509. Found: 652.4557; HPLC analysis: peak area 96.2%, t_R_ = 9.59 min, gradient: 90% (3 min to 90%) to 95% B over 5 min and from 95 to 100% B over 10 min.

**6b**: compound **6b** was prepared using 1.1 equivalents of 4-methoxybenzylamine at room temperature for 24 h. Yield = 60%; Colorless oil; R_f_ (PhMe/EtOAc 9:1) = 0.15; ^1^H NMR (600 MHz, CDCl_3_) *δ* 7.24–7.20 (m, 2 H), 6.88–6.84 (m, 2 H), 4.89 (br s, 1 H), 4.68 (d, *J* = 2.5 Hz, 1 H), 4.58 (t, *J* = 1.5 Hz, 1 H), 4.34–4.25 (m, 3 H), 3.88 (d, *J* = 10.6 Hz, 1 H), 3.80 (s, 3 H), 3.18 (dd, *J* = 11.5, 4.7 Hz, 1 H), 2.45 (tt, *J* = 10.8, 5 Hz, 1 H), 1.99 (h, *J* = 9.9 Hz, 2 H), 1.81 (d, *J* = 13.0 Hz, 1 H), 1.79–1.72 (m, 2 H), 1.71–1.69 (m, 1 H), 1.68 (s, 3 H), 1.62–1.50 (m, 9 H), 1.43–1.35 (m, 5 H), 1.26 (s, 3 H), 1.21 (dd, *J* = 13.2, 4.9 Hz, 2 H), 1.11 (s, 1 H), 1.04 (s, 3 H), 0.97 (s, 3 H), 0.93–0.86 (m, 1 H), 0.83 (s, 3 H), 0.76 (s, 3 H), 0.68 (d, *J* = 9.4 Hz, 1 H); ^13^C NMR (151 MHz, CDCl_3_) *δ* 129.0, 128.2, 114.1, 109.8, 79.0, 55.3, 50.4, 48.8, 47.7, 46.6, 42.7, 40.9, 38.9, 38.7, 37.5, 37.1, 34.2, 29.7, 28.0, 27.4, 27.1, 25.2, 20.8, 18.3, 16.1, 16.1, 15.3, 14.7; HR-ESI (*m*/*z*) [M + H^+^] calculated for C_39_H_60_NO_4_ 606.4522. Found: 606.4520; HPLC analysis: peak area 100%, t_R_ = 8.72 min, gradient: 90% (3 min to 90%) to 95% B over 5 min and from 95 to 100% B over 10 min.

**6c**: compound **6c** was prepared using 1.1 equivalents of 3,4-dimethoxybenzylamine at room temperature for 1 h. Yield = 66%; Colorless oil; R_f_ (PhMe/EtOAc 9:1) = 0.09; ^1^H NMR (600 MHz, CDCl_3_) *δ* 6.84 (s, 1 H), 6.82 (s, 2 H), 4.92 (s, 1 H), 4.69 (d*, J* = 2.5 Hz, 1 H), 4.58 (t, *J* = 1.5 Hz, 1 H), 4.31 (s, 2 H), 4.29 (s, 1 H), 3.87 (s, 3 H), 3.87 (s, 3 H), 3.18 (dd, *J* = 11.4, 4.8 Hz, 1 H), 2.45 (td, *J* = 11.1, 5.7 Hz, 1 H), 1.99 (qui, *J* = 9.9 Hz, 1 H), 1.82 (d, *J* = 13.7 Hz, 1 H), 1.79–1.72 (m, 3 H), 1.68 (s, 3 H), 1.66–1.11 (m, 19 H), 1.04 (s, 3 H), 0.97 (s, 6 H), 0.92–0.86 (m, 1 H), 0.82 (s, 3 H), 0.76 (s, 3 H), 0.68 (d, *J* = 8.4 Hz, 1 H); ^13^C NMR (151 MHz, CDCl_3_) *δ* 149.2, 137.9, 129.0, 128.2, 125.3, 111.2, 111.0, 109.8, 79.0, 56.0, 55.9, 55.3, 50.4, 48.8, 47.7, 46.6, 42.7, 40.9, 38.8, 38.7, 37.5, 34.5, 34.2, 29.8, 29.6, 28.0, 27.4, 27.1, 25.2, 21.4, 20.8, 19.1, 18.3, 16.1, 16.0, 15.3, 14.8; HR-ESI (*m*/*z*) [M + H^+^] calculated for C_40_H_62_NO_5_ 636.4628. Found: 636.4631; HPLC analysis: peak area 95.1%, t_R_ = 8.56 min, gradient: 90% (3 min to 90%) to 95% B over 5 min and from 95 to 100% B over 10 min.

**6d**: compound **6d** was prepared using 1.1 equivalents of 3,4,5-trimethoxybenzylamine at room temperature for 1.5 h. Yield = 62%; White solid; m.p.: 153.154.5 °C; R_f_ (PhMe/EtOAc 9:1) = 0.14; ^1^H NMR (600 MHz, CDCl_3_) *δ* 6.52 (s, 2 H), 4.96 (s, 1 H), 4.69 (d, *J* = 2.3 Hz, 1 H), 4.59 (t, *J* = 1.5 Hz, 1 H), 4.33 (s, 1 H), 4.31 (s, 2 H), 3.90 (d, *J* = 11.4 Hz, 1 H), 3.85 (s, 6 H), 3.83 (s, 3 H), 3.18 (dd, *J* = 11.4, 4.7 Hz, 1 H), 2.45 (td, *J* = 11.1, 5.8 Hz, 1 H), 2.04–1.94 (m, 1 H), 1.82 (d, *J* = 14.0 Hz, 1 H), 1.79–1.72 (m, 3 H), 1.68 (s, 3 H), 1.61–1.47 (m, 9 H), 1.44–1.34 (m, 5 H), 1.29–1.18 (m, 4 H), 1.05 (s, 3 H), 0.97 (s, 6 H), 0.90 (dd, *J* = 12.7, 3.7 Hz, 1 H), 0.82 (s, 3 H), 0.76 (s, 3 H), 0.68 (d, *J* = 9.2 Hz, 1 H); ^13^C NMR (151 MHz, CDCl_3_) *δ* 153.4, 134.3, 109.8, 104.6, 79.0, 60.8, 56.1, 55.3, 50.4, 48.8, 47.7, 46.6, 42.7, 40.9, 38.9, 38.7, 37.5, 37.1, 34.5, 34.2, 29.8, 29.6, 28.0, 27.4, 27.1, 25.2, 20.8, 19.1, 18.3, 16.1, 16.0, 15.3, 14.8; HR-ESI (*m*/*z*) [M + H^+^] calculated for C_41_H_64_NO_6_ 666.4733. Found: 666.4730; HPLC analysis: peak area 95.0%, t_R_ = 8.49 min, gradient: 90% (3 min to 90%) to 95% B over 5 min and from 95 to 100% B over 10 min.

**6e**: compound **6e** was prepared using 1.1 equivalents of 4-methoxyphenylethylamine at room temperature for 24 h. Yield = 55%; Colorless oil; R_f_ (PhMe/EtOAc 95:5) = 0.1; ^1^H NMR (600 MHz, CDCl_3_) *δ* 7.10 (d, *J* = 8.5 Hz, 2 H), 6.84 (d, *J* = 8.5 Hz, 2 H), 4.69 (d, *J* = 2.3 Hz, 1 H), 4.65 (br s, 1 H), 4.58 (t, *J* = 1.7 Hz, 1 H), 4.25 (dd, *J* = 10.9, 1.9 Hz, 1 H), 3.83 (d, *J* = 11.6 Hz, 1 H), 3.79 (s, 3 H), 3.45–3.37 (m, 2 H), 3.18 (dd, *J* = 11.5, 4.8 Hz, 1 H), 2.76 (t, *J* = 6.8 Hz, 2 H), 2.54–2.37 (m, 1 H), 1.97 (m, 1 H), 1.88–1.70 (m, 3 H), 1.68 (s, 3 H), 1.66–1.48 (m, 7 H), 1.44–1.35 (m, 5 H), 1.29–1.77 (m, 5 H), 1.11 (s, 1 H), 1.04 (s, 3 H), 1.02 (s, 1 H), 0.97 (m, 6 H), 0.91 (dd, *J* = 12.9, 3.7 Hz, 1 H), 0.82 (s, 3 H), 0.76 (s, 3 H), 0.68 (d, *J* = 9.6 Hz, 1 H); ^13^C NMR (151 MHz, CDCl_3_) *δ* 158.3, 157.0, 150.5, 150.3, 129.7, 129.0, 128.2, 125.3, 114.0, 109.7, 79.0, 63.0, 60.5, 55.3, 50.4, 48.8, 47.8, 47.7, 46.6, 42.7, 40.9, 38.9, 38.7, 37.5, 37.1, 34.2, 29.7, 29.2, 28.0, 27.4, 27.1, 25.2, 21.4, 20.8, 19.1, 18.3, 16.1, 16.0, 15.3, 14.7; HR-ESI (*m*/*z*) [M + H^+^] calculated for C_40_H_62_NO_4_ 620.4679. Found: 620.4676; HPLC analysis: peak area 98.0%, t_R_ = 9.32 min, gradient: 90% (3 min to 90%) to 95% B over 5 min and from 95 to 100% B over 10 min.

**6f**: compound **6f** was prepared using 1.1 equivalents of 3,4-dimethoxyphenylethylamine at room temperature for 72 h. Yield = 50%; Colorless oil; Rf (PhMe/EtOAc 9:1) = 0.10; ^1^H NMR (600 MHz, CDCl_3_) *δ* 6.81 (d, *J* = 8.1 Hz, 1 H), 6.74–6.69 (m, 2 H), 4.68 (d, *J* = 2.6 Hz, 1 H), 4.67–4.64 (m, 1 H), 4.58 (t, *J* = 1.7 Hz, 1 H), 4.26 (d, *J* = 12.4 Hz, 1 H), 3.87 (s, 3 H), 3.86 (s, 3 H), 3.84–3.81 (m, 1 H), 3.47–3.36 (m, 2 H), 3.18 (dd, *J* = 11.4, 4.7 Hz, 1 H), 2.76 (t, *J* = 6.9 Hz, 2 H), 2.44 (td, *J* = 10.7, 5.7 Hz, 1 H), 1.98 (qui, *J* = 9.1 Hz, 1 H), 1.81–1.69 (m, 3 H), 1.67 (s, 3 H), 1.65–1.48 (m, 7 H), 1.39 (m, 6 H), 1.30–1.18 (m, 5 H), 1.04 (s, 3 H), 1.02–0.99 (m, 1 H), 0.96 (s, 6 H), 0.89 (td, *J* = 12.9, 4.0 Hz, 1 H), 0.82 (s, 3 H), 0.76 (s, 3 H), 0.67 (d, *J* = 9.4 Hz, 1 H) ^13^C NMR (151 MHz, CDCl_3_) *δ* 158.4, 157.0, 149.0, 147.7, 120.7, 118.8, 112.0, 111.4, 109.8, 79.0, 55.9, 55.8, 55.3, 50.4, 48.8, 47.7, 46.6, 42.7, 42.3, 40.9, 38.9, 38.7, 37.5, 37.1, 35.7, 34.2, 29.6, 28.0, 27.4, 27.1, 25.2, 20.8, 19.1, 18.3, 16.1, 16.0, 15.3, 14.7; HR-ESI (*m*/*z*) [2 M + H^+^] calculated for [C_41_H_63_NO_5_] 1299.9492. Found: 1299.9467. HPLC analysis: peak area 96.4%, t_R_ = 8.35 min, gradient: 90% (3 min to 90%) to 95% B over 5 min and from 95 to 100% B over 10 min.

**6g**: compound **6g** was prepared using 1.1 equivalents of 3,4,5-trimethoxyphenylethylamine at room temperature for 72 h. Yield = 55%; Colorless oil; Rf (PhMe/EtOAc 9:1) = 0.25; ^1^H NMR (600 MHz, CDCl_3_) *δ* 6.40 (s, 2 H), 4.71–4.69 (m, 1 H), 4.69 (d, *J* = 2.5 Hz, 1 H), 4.58 (t, *J* = 1.9 Hz, 1 H), 4.27 (d, *J* = 10.9 Hz, 1 H), 3.88–3.86 (m, 1 H), 3.85 (s, 6 H), 3.83 (s, 3 H), 3.44 (qui, *J* = 6.4 Hz, 2 H), 3.18 (dd, *J* = 11.4, 4.8 Hz, 1 H), 2.76 (t, *J* = 7.2 Hz, 2 H), 2.49–2.39 (m, 1 H), 1.99 (qui, *J* = 9.2 Hz, 1 H), 1.82–1.71 (m, 3 H), 1.68 (s, 3 H), 1.65–1.48 (m, 8 H), 1.39 (m, 6 H), 1.29–1.17 (m, 4 H), 1.04 (s, 3 H), 1.03–1.00 (m, 1 H), 0.97 (s, 6 H), 0.90 (td, *J* = 12.9, 4.0 Hz, 1 H), 0.82 (s, 3 H), 0.76 (s, 3 H), 0.68 (d, *J* = 9.4 Hz, 1 H); ^13^C NMR (151 MHz, CDCl_3_) *δ* 157.0, 153.3, 150.2 137.9, 129.0, 128.2, 125.3, 109.8, 105.7, 79.0, 63.0, 60.8, 56.1, 55.3, 50.4, 48.8, 47.7, 46.6, 42.7, 42.2, 40.9, 38.8, 38.7, 37.5, 37.1, 36.6, 34.5, 34.2, 29.8, 29.6, 28.0, 27.4, 27.1, 25.2, 21.4, 20.8, 19.1, 18.3, 16.1, 16.0, 15.3, 14.8; HR-ESI (*m*/*z*) [M + H^+^] calculated for C_42_H_66_NO_6_ 680.4890. Found: 680.4791; HPLC analysis: peak area 95.2%, t_R_ = 8.99 min, gradient: 90% (3 min to 90%) to 95% B over 5 min and from 95 to 100% B over 10 min.

**6h**: compound **6h** was prepared using 20 equivalents of piperazine at room temperature for 18 h. Yield = 75%; Colorless oil; Rf (DCE/MeOH 9:1) = 0.36; ^1^H NMR (600 MHz, CDCl_3_) *δ* 4.68 (d, *J* = 2.6 Hz, 1 H), 4.58 (t, *J* = 1.7 Hz, 1 H), 4.27 (d, *J* = 10.7 Hz, 1 H), 3.86 (d, *J* = 10.8 Hz, 1 H), 3.72 (s, 1 H), 3.51 (br s, 4 H), 3.17 (dd, *J* = 11.4, 4.8 Hz, 1 H), 2.89 (br s, 4 H), 2.80–2.62 (m, 2 H), 2.45 (td, *J* = 11.1, 5.0 Hz, 1 H), 2.03–1.93 (m, 1 H), 1.83–1.79 (m, 1 H), 1.78–1.69 (m, 3 H), 1.67 (s, 3 H), 1.66–1.48 (m, 5 H), 1.43–1.33 (m, 5 H), 1.31–1.15 (m, 4 H), 1.10–1.04 (m, 2 H), 1.03 (s, 3 H), 0.97 (s, 3 H), 0.96 (s, 3 H), 0.89 (td, *J* = 12.9, 4.2 Hz, 1 H), 0.81 (s, 3 H), 0.75 (s, 3 H), 0.67 (d, *J* = 9.3 Hz, 1 H); ^13^C NMR (151 MHz, CDCl_3_) *δ* 155.8, 150.2, 129.0, 128.2, 109.8, 78.9, 63.9, 55.3, 50.4, 48.8, 47.7, 46.6, 45.3, 43.4, 42.7, 40.9, 38.8, 38.7, 37.5, 37.1, 34.6, 34.2, 30.0, 29.6, 28.0, 27.4, 27.1, 25.2, 20.8, 19.1, 18.3, 16.1, 16.1, 15.4, 14.8; HR-ESI (*m*/*z*) [M + H^+^] calculated for C_35_H_59_N_2_O_3_ 555.4457. Found 555.4487; HPLC analysis: peak area 98.2%, t_R_ = 12.34 min, gradient: 45% (3 min to 45%) to 100% B over 20 min.

**6i**: compound **6i** was prepared using 1.1 equivalents of *N*-methylpiperazine without DIPEA at room temperature for 15 h. Yield = 68%; White solid; m.p.: 189–190 °C; Rf (PhMe/EtOAc 1:9) = 0.13; ^1^H NMR (600 MHz, CDCl_3_) *δ* 4.69 (d, *J* = 2.5 Hz, 1 H), 4.59 (t, *J* = 1.7 Hz, 1 H), 4.29 (d, *J* = 9.3 Hz, 1 H), 3.88 (d, *J* = 10.9 Hz, 1 H), 3.68 (br s, 4 H), 3.18 (dd, *J* = 11.6, 4.8 Hz, 1 H), 2.78–2.48 (m, 4 H), 2.47–2.39 (m, 1 H), 2.02–1.94 (m, 1 H), 1.83–1.69 (m, 4 H), 1.68 (s, 3 H), 1.66–1.47 (m, 6 H), 1.43–1.36 (m, 5 H), 1.30–1.15 (m, 7 H), 1.11–1.05 (m, 2 H), 1.04 (s, 3 H), 0.98 (s, 3 H), 0.97 (s, 3 H), 0.93–0.85 (m, 2 H), 0.82 (s, 3 H), 0.76 (s, 3 H), 0.68 (d, *J* = 9.6 Hz, 1 H); ^13^C NMR (151 MHz, CDCl_3_) *δ* 155.6, 150.1, 129.0, 128.2, 125.3, 109.9, 91.5, 79.0, 64.2, 55.3, 54.2, 50.4, 48.8, 47.7, 46.6, 42.7, 40.9, 38.9, 38.7, 37.5, 37.1, 34.6, 34.2, 30.0, 29.6, 28.0, 27.4, 27.1, 25.2, 20.8, 19.1, 18.3, 16.1, 16.1, 15.3, 14.8; HR-ESI (*m*/*z*) [M + H^+^] calculated for C_36_H_61_N_2_O_3_ 569.4682. Found: 569.4669; HPLC analysis: peak area 98.0%, t_R_ = 9.65 min, gradient: 25% (3 min to 25%) to 75% B over 20 min.

**6j**: compound **6j** was prepared using 1.1 equivalents of tert-butyl-4-(2-aminoethyl) piperazine-1-carboxylate at room temperature for 20 h. Yield = 70%; Colorless oil; Rf (EtOAc) = 0.4; ^1^H NMR (600 MHz, CDCl_3_) *δ* 5.08 (s, 1 H), 4.68 (d, *J* = 2.6 Hz, 1 H), 4.58 (t, *J* = 2.0 Hz, 1 H), 4.30–4.24 (m, 2 H), 3.85 (d, *J* = 11.1 Hz, 1 H), 3.71 (td, *J* = 5.8, 1.7 Hz, 2 H), 3.42 (br s, 4 H), 3.30 (br s, 1 H), 3.18 (dd, *J* = 11.6, 4.8 Hz, 1 H), 2.50–2.47 (m, 1 H), 2.39 (br s, 4 H), 2.02–1.95 (m, 1 H), 1.85 (d, *J* = 12.9 Hz, 1 H), 1.79 (dd, *J* = 12.4, 7.3 Hz, 1 H), 1.74 (d, *J* = 12.5 Hz, 1 H), 1.68 (s, 3 H), 1.65–1.48 (m, 9 H), 1.46 (s, 9 H), 1.43–1.35 (m, 5 H), 1.31–1.22 (m, 5 H), 1.04 (s, 3 H), 0.97 (s, 3 H), 0.96 (s, 3 H), 0.92–0.85 (m, 1 H), 0.82 (s, 3 H), 0.76 (s, 3 H), 0.68 (d, *J* = 9.3 Hz, 1 H); ^13^C NMR (151 MHz, CDCl_3_) *δ* 171.1, 157.1, 154.7, 150.3, 129.0, 128.2, 125.3, 109.8, 79.7, 79.0, 64.3, 63.1, 57.2, 55.3, 52.7, 50.4, 48.8, 47.7, 46.6, 42.7, 40.9, 38.9, 38.7, 37.5, 37.1, 34.6, 34.2, 30.6, 29.7, 28.4, 28.0, 27.4, 27.1, 25.2, 20.8, 19.1, 18.3, 16.1, 16.0, 15.3, 14.8, 13.7; HR-ESI (*m*/*z*) [M + H^+^] calculated for C_42_H_72_N_3_O_5_ 698.5472. Found: 698.5447; HPLC analysis: peak area 98.7%, t_R_ = 8.27 min, gradient: 25% (3 min to 25%) to 75% B over 20 min.

**6l**: compound **6l** was prepared using 1.1 equivalents of 4-(4-Methylpiperazino) benzylamine at room temperature for 24 h. Yield = 42%; White solid; m.p.: 151–152 °C; Rf (EtOAc/Et_3_N 10:0.1) = 0.33; ^1^H NMR (600 MHz, CDCl_3_) *δ* 7.21–7.17 (m, 2 H), 6.90 (s, 1 H), 6.88 (s, 1 H), 4.88 (br s, 1 H), 4.68 (d, *J* = 2.5 Hz, 1 H), 4.58 (t, *J* = 1.9 Hz, 1 H), 4.30–4.24 (m, 3 H), 3.87 (d, *J* = 11.2 Hz, 1 H), 3.29 (br s, 4 H), 3.18 (d, *J* = 11.1 Hz, 1 H), 2.72 (br s, 4 H), 2.48–2.41 (m, 4 H), 2.01–1.94 (m, 1 H), 1.84–1.72 (m, 3 H), 1.67 (s, 3 H), 1.63–1.48 (m, 6 H), 1.44–1.34 (m, 5 H), 1.29–1.17 (m, 6 H), 1.04 (s, 3 H), 1.02–0.99 (m, 1 H), 0.96 (s, 6 H), 0.89 (td, *J* = 13.1, 4.4 Hz, 2 H), 0.82 (s, 3 H), 0.76 (s, 3 H), 0.67 (d, *J* = 9.5 Hz, 1 H); ^13^C NMR (151 MHz, CDCl_3_) *δ* 171.1, 157.0, 150.3, 129.0, 128.8, 128.2, 116.4, 109.7, 79.0, 63.2, 60.4, 55.3, 54.7, 50.4, 48.8, 48.6, 47.7, 46.6, 44.6, 42.7, 40.9, 38.9, 38.7, 37.5, 37.1, 34.5, 34.2, 29.8, 29.7, 28.0, 27.4, 27.1, 25.2, 21.0, 20.8, 19.1, 18.3, 16.1, 16.1, 15.3, 14.7, 14.2; HR-ESI (*m*/*z*) [M + H^+^] calculated for C_43_H_68_N_3_O_3_ 674.5260. Found: 674.5258; HPLC analysis: peak area 96.4%, t_R_ = 11.5 min, gradient: 50% (3 min to 50%) to 100% B over 20 min.

**6m**: compound **6m** was prepared using 1.1 equivalents of 6,7-dimethoxy-1,2,3,4-tetrahydro-isoquinoline at room temperature for 17 h. Yield = 42%; Rf (PhMe/EtOAc9:1) = 0.18; Colorless oil; ^1^H NMR (600 MHz, CDCl_3_) *δ* 6.62 (s, 1 H), 6.60 (br s, 1H), 4.69 (d, *J* = 2.6 Hz, 1 H), 4.59 (t, *J* = 1.9 Hz, 1 H), 4.55 (d, *J* = 10.3 Hz, 2 H), 4.31 (d, *J* = 10.4 Hz, 1 H), 3.90 (d, *J* = 10.9 Hz, 1 H), 3.86 (s, 6 H), 3.72–3.63 (m, 2 H), 3.18 (dd, *J* = 11.5, 4.7 Hz, 1 H), 2.77 (s, 2 H), 2.48 (td, *J* = 11.2, 5.8 Hz, 1 H), 2.06–1.96 (m, 1 H), 1.93–1.81 (m, 2 H), 1.78–1.70 (m, 2 H), 1.69 (s, 3 H), 1.67–1.48 (m, 8 H), 1.45–1.35 (m, 5 H), 1.31–1.17 (m, 4 H), 1.07–1.05 (m, 1 H), 1.04 (s, 3 H), 0.99 (s, 3 H), 0.97 (s, 3 H), 0.90 (td, *J* = 13.5, 4.7 Hz, 1 H), 0.82 (s, 3 H), 0.76 (s, 3 H), 0.68 (d, *J* = 9.4 Hz, 1 H); ^13^C NMR (151 MHz, CDCl_3_) *δ* 150.2, 147.7, 147.7, 137.9, 129.0, 128.2, 125.3, 111.5, 109.8, 79.0, 63.8, 56.0, 56.0, 55.3, 50.4, 48.8, 47.7, 46.7, 42.7, 40.9, 38.9, 38.7, 37.5, 37.2, 34.7, 34.2, 30.1, 29.7, 28.0, 27.4, 27.2, 25.2, 21.4, 20.8, 19.2, 18.3, 16.1, 15.3, 14.8; HR-ESI (*m*/*z*) [M + H^+^] calculated for C_42_H_64_NO_5_ 662.4784. Found: 662.4775; HPLC analysis: peak area 98.6%, t_R_ = 10.90 min, gradient: 90% (3 min to 90%) to 95% B over 5 min and from 95 to 100% B over 10 min.

### Deprotection of compound 6j

To an ice-cold solution of **6j** (9 mg, 0.01 mmol) in DCM (40 μL), trifluoroethanol (0.05 mmol, 5μL) and TFA (0.25 mmol, 20μL) were added and the reaction mixture was stirred at ambient temperature for 3 h. Volatile components were evaporated under vacuum, the oily residue was triturated with a mixture of diethyl ether (Et_2_O)/hexane, and refrigerated overnight. The desired trifluoroacetate salt **6k** was afforded as pale-yellow oil. Yield = 90%; HR-ESI (*m*/*z*) [M^+^] calculated for C_37_H_65_N_3_O_3_^+^ 598.4942. Found: 598.4932; HPLC analysis: peak area 98.4%, t_R_ = 8.28 min, gradient: 25% (3 min to 25%) to 75% B over 20 min.

### Material for biological studies

3-(4,5-Dimethyl-2-thiazolyl)-2,5-diphenyl-2H-tetrazolium bromide (MTT), histopaque 1077, lectin from *Phaseolus vulgaris* (PHA), and Rho123 were obtained from Sigma Aldrich (Sigma-Aldrich Co., St Louis, MO). Doxorubicin hydrochloride (Dox, 99.8%, Synbias Pharma Ltd.) was obtained from Nanox Release Technology (Buenos Aires, Argentina) and was prepared immediately prior to use dissolved in bi-distilled water. Verapamil hydrochloride 98.0% was provided by Parafarm (Buenos Aires, Argentina) while tariquidar was purchased from ChemFaces Biochemical Co., Ltd (Wuhan, China). Both were prepared in DMSO. Sterile plastic material was purchased from Greiner Bio-One (Frickenhausen, Germany). Flow cytometry was carried out in a Life Technologies Attune-NxT flow cytometer with 96-well autosampler (Thermo Fisher Scientific, USA).

### Cell lines and cell culture

The sensitive CML cells K562 and its counterpart P-gp-overexpressing K562/Dox cells, formerly Lucena 1 with MDR phenotype mainly characterized by the overexpression of ABCB1/P-gp^54^, were cultured in RPMI-1640 medium (Invitrogen Life Technologies, Carlsbad, CA) supplemented with 10% fetal bovine serum, 2 mM L-glutamine, 100 U/mL penicillin, and 100 μg/mL streptomycin (Invitrogen Life Technologies, Carlsbad, CA) at 37 °C in a 5% CO_2_ humidified atmosphere. Both cell lines were provided by Dr. V. Rumjanek, Universidade Federal do Rio de Janeiro, Rio de Janeiro, Brazil^54^. Passages were performed twice a week and cells in exponential growth phase with a viability of 90%, determined by trypan blue exclusion, were used for the experiments. To give rise to the MDR phenotype, resistant lines were subjected to continuous treatment with 60 nM of Dox. This treatment was interrupted 4 days before each trial. K562/Dox displayed 32.3-fold more resistance to Dox than K562, as evidenced by the IC_50_ values obtained by MTT assay (IC_50_ = 84.21 ± 7.08 and 2.61 ± 0.70 µM, respectively). This level of resistance correlates with the level of expression of P-gp^31^.

### MTT colorimetric assay

The cytotoxic effect of the compounds and of the gold standards verapamil and tariquidar was determined against K562/Dox and K562 using MTT assay. This colorimetric technique is based on the ability of viable cells to capture MTT and reduce it through the mitochondrial enzyme succinic dehydrogenase to its insoluble form, formazan crystals^55^. The target compounds dissolved in DMSO (0.5% v/v as final concentration which was non-toxic to cells) at a maximum concentration of 50 µM were exposed to 5 × 10^4^ cells distributed in a 96-well plate and incubated for 48 h. At the end of the incubation period, the cells were treated with 5 mg/mL of MTT solution prepared in phosphate buffered saline (PBS) and incubated at 37 °C in a humid atmosphere with 5% CO_2_ for another 4 h. After this time, the plate was centrifuged at 2000 rpm for 10 min, and 100 µL of DMSO was added to dissolve the formazan crystals formed. Absorbance (Abs) was read at a wavelength of 595 nm in an iMark microplate reader (Bio-Rad, USA). The percentages of cytotoxicity were determined as compared to DMSO treated control cells, which were considered 100% viable as showing the same level of growth as the untreated cells. Those compounds showing a cytotoxicity > 55% at 50 µM were further assayed with a range of two-fold decreasing concentrations for their IC_50_ values, which were calculated as previously reported^56,57^ using non-linear regression by GraphPad Prism 7 (GraphPad Software, Inc., San Diego, CA, USA, www.graphpad.com).

### Rhodamine 123 and doxorubicin intracellular accumulation assays

The ability of the compounds to increase the intracellular concentration of Rho123 or of Dox by the inhibition of P-gp was evaluated using flow cytometry, as described earlier^32,33,58^. K562/Dox or K562 cells adjusted to a density of 5 × 10^4^ cells/well were seeded in 96-well plates, previously containing RPMI-1640 medium in the presence of each compound dissolved in DMSO, at concentrations ranging from 0.049 to 50 µM and from 6.25 to 50 µM for each cell line, respectively. Cells treated only with DMSO at 0.5% v/v were used as negative controls, while those treated with the standard P-gp inhibitors^42^, verapamil and tariquidar were used as positive controls. No differences between negative controls and untreated cells were observed. The samples were incubated for 1 h at 37 °C in a humid atmosphere with 5% CO_2_. At the end of the incubation period, the corresponding fluorescent substrate, Rho123 (500 ng/mL) or Dox (5 μM), was added, and the cells were incubated for another 1 h. Subsequently, cells were thoroughly washed twice with cold PBS and kept in dark and cold. Samples were gated on a forward versus size scatter, and 15,000 events from each were analyzed to determine the cell-associated fluorescence. It is important to notice that the target derivatives were not fluorescent by themselves (data not shown).

Dox and Rho123 were excited at 488 nm, and the fluorescence emitted was collected with 585/42 nm or 530/30 nm bandpass filters, respectively. Mean fluorescence intensity (MFI) was analyzed using Flowjo software (Tree Star, Inc. Ashland, OR). The blocking of the P-gp transport function was evaluated by the parameter fluorescence activity ratio (FAR), calculated as the ratio of the MFI of Rho123 or Dox with the addition of the likely inhibitor to the MFI of Rho123 or Dox alone.

### Doxorubicin resistance reversal assay

These experiments were undertaken to evaluate the ability of the two most effective compounds to potentiate Dox cytotoxicity by inhibiting the outward transport mediated by P-gp. MTT staining was used, for which 5 × 10^4^ K562/Dox and K562 cells/well and increasing concentrations of Dox (3.4–431 and 0.33–34.5 µM, respectively) in the absence or presence of 0.024 to 1.56 µM and 0.19 to 1.56 µM of compounds **6g** and **6i,** respectively, were incubated in 96-well plates containing RPMI-1640 medium. DMSO (0.5% v/v) was used as a negative control, while verapamil was chosen as standard inhibitor. Simultaneously, viability controls were run with no addition of the dissolution solvent. The cells were incubated at 37 °C at 5% CO_2_ for 48 h and the same protocol described above was followed. The potency of the compounds was established by means of the fold reversal (FR) values calculated from dividing the IC_50_ of Dox alone by the IC_50_ of Dox with different concentrations of each compound tested.

### Cytotoxicity on peripheral blood mononuclear cells and hemolysis assay

In order to preliminarily determine the safety of the active compounds **6g** and **6i** in normal cells, different concentrations (1.56–50 µM) of these were assayed by the MTT technique on peripheral blood mononuclear cells (PBMC) according to published works^33,58^. PBMC were isolated by the Ficoll-Hypaque density gradient centrifugation technique of fresh heparinized blood from healthy human volunteers. The protocol was previously approved by the Catholic University of Córdoba Research Ethics Board. Signed informed consents were obtained from donors. All methods were performed in accordance with the relevant guidelines and regulations. Briefly, 1 × 10^5^ cells/well in a 96-well plate were incubated with 10 µg/mL PHA, in the presence of the corresponding concentrations of the compounds or 0.5% v/v DMSO (negative control) for 48 h, and processed as above described for the MTT method. The percentages of inhibition on cell proliferation were determined by comparison with negative controls, and the corresponding IC_50_ values were calculated from the best fit of the concentration-dependent inhibition curves in GraphPad Prism 7.

For the erythrocyte hemolysis assay, the red blood cell suspension was obtained from fresh heparinized blood collected from healthy human volunteer donors, without receiving any treatment. The blood sample was centrifuged at 2,500 rpm for 10 min at 4 °C. The supernatant was discarded, and the red blood cell pellet was subjected to two consecutive washes with sterile PBS, using the same centrifugation conditions. An aliquot of the pellet obtained was resuspended in PBS (3% v/v). The compounds were incubated at decreasing concentrations starting from 50 µM with the suspension at 37 °C for 1 h. Controls with 1% DMSO and no solvent were included.

### Statistical analysis

The results are expressed as the mean ± standard error (SE). Data were analyzed using the one-tailed paired Student's t-test and one-tailed unpaired Student's t-test, as appropriate, by GraphPad Prism 7.0. *p* values  ≤ 0.05 were considered statistically significant. Each experiment was performed in triplicate or quadruplicate and done independently at least three times.

### Molecular modeling

The structural model of the P-gp was based on the structure cocrystallized with paclitaxel (PDB entry 6QEX)^51^, and prepared as described in detail in Laiolo et al.^33^. In this work, the structure 7A69, cocrystallized with the antitumoral agent vincristine^52^, was also used for checking the docking procedure, although the simulations of vincristine were made on the same receptor (based on 6QEX) used for the rest of the substrates and inhibitors.

The docking protocol was fairly similar to that in Laiolo et al.^33^. Briefly, the box included the whole TM region (no assumption about a particular binding site) and the docking was run with Autodock Vina 1.1.2-5^59^, collecting the first 12 lowest energy poses or those within 3 kcal/mol above the lowest. The “exhaustiveness” parameter was set between 64 and 192 (much bigger than the default, 8, due to the size of the region), and repeating the simulation at least 12 times. Standard Gagsteiger’s charges^59^ were set for all ligands except for Rho123, for which RESP charges^60^ at the CAM-B3LYP/6–31 + G(d) level of theory^61^ were used to correctly draw the charge delocalization of the cationic dye. The structure of the ligands were obtained by full geometry optimization with the semiempirical PM6 Hamiltonian as implemented in Gaussian 16 Rev. A03^62^ The structures were confirmed as minima by diagonalization of the Hessian matrix.

The initial structures for the MD simulations were prepared from the most stable docked structures using the AMBER18^63^ utilities LEaP, parmchk2 and antechamber^64^. The atomic charges on the ligands were obtained with the AMBER18 module sqm, using AM1 quantum calculations and the parameters assigned based on the auxiliary force field GAFF.^63^. The protocol for running the MD simulations was successfully applied in Laiolo et al.^32,33^. This involves: (I) 250 steepest descent minimization steps of the whole system, keeping the protein tightly restrained and embedded into a box of TIP3P water molecules with a minimum distance of 10 Å to each wall, and enough chloride counter-ions to reach electro-neutrality; (II) 6500 conjugate gradient minimization steps of the whole system; (III) 100 ps slowly heating in the NTV ensemble with the protein positionally restrained in the backbone; (IV) 60 ns of simulation in the NTP ensemble, at 1 atm and 300 K. Procedures III-IV were repeated in two or three independent trajectories using the Andersen thermostat and barostat^65^. In the reasonably equilibrated system, the RMSd of the ligand and all residues surrounding it within 5 Å was in most cases between 1.1 and 1.6 Å, excluding hydrogens (examples in Supplementary Material Fig. S6). Due to the transmembrane nature of P-gp, a 50.0 kcal/Å^2^ harmonic restraint was kept for the backbone atoms. Electrostatic interactions were computed using the Particle Mesh Ewald (PME) method with a cutoff of 10 Å^66^. The SHAKE algorithm, as implemented in pmemd.cuda, was applied to constrain hydrogen-heavy atom bonds^63^. The force field used for the atoms of the protein was ff14SB^67^.The trajectory analyses were made with cpptraj^63^ and VMD 1.9.3^68^, with the latter also used for graphics rendering.

The free energy calculations were made using the mmpbsa module of AMBER 18 by applying Poisson–Boltzman (PB) and Generalized Born (GB) models^69^. The energy analyses were made for the last 8–12 ns of simulation as the average over at least two independent trajectories. The frames were sampled once each 10–20 ps in order to keep the energy self-correlation negligible. A detailed analysis of the dynamics of the contacts was made for all simulated compounds by partitioning the energy contribution of each amino acid with the inhibitor from one representative run as shown for Rho123, vincristine, and compounds **6g**, **6i**, **6f**, **3a,** and **3d** displayed in Fig. 11. For comparison purposes, the pattern obtained for compound **6 m** (which slightly departs in its mode of binding from the subset of compounds that behave as compound **6i**) is shown in the Supplementary Material Fig. S5, together with data previously obtained using the same modeling models for doxorubicin and tariquidar as other reference agents run as substrate and inhibitor, respectively^33^.

The average total number of H-bonds were in general between 0.5 and 1.5 during the equilibrated trajectories, with some examples shown as Supplementary Material Fig. S7. Chimera 1.15^70^ was used for clustering analyses, using the last 50 ns of each trajectory and for the graphics rendering of representative conformations thus obtained.

### Supplementary Information

Supplementary Information.

## Data Availability

All data generated or analyzed during this study are included in this published article (and its Supplementary Information files).

## Abbreviations

CML: Chronic myelogenous leukemia
DCC: N,N΄-dicyclohexylcarbodiimide
DIPEA: N,N΄-diisopropylehtylamine
DMAP: 4-Dimethylaminopyridine
Dox: Doxorubicin
FR: Fold reversal
HOBt: 1-Hydroxybenzotriazole
MDR: Multidrug resistance
NBD: Nucleotide binding domain
Rho123: Rhodamine 123
TFE: 2,2,2-Trifluoroethanol
