# Supplementary material for: Novel betulin derivatives as multidrug reversal agents targeting P-glycoprotein
Source: Sci Rep. 2024 Jan 2;14:70. doi: 10.1038/s41598-023-49939-9 (PMC10762177; doi:10.1038/s41598-023-49939-9)
Supplement: Supplementary file 1 — Supplementary Information. [file 41598_2023_49939_MOESM1_ESM.docx]

**SUPPORTING INFORMATION**

**Novel betulin derivatives as multidrug reversal agents targeting P-glycoprotein**

**Jerónimo Laiolo^1, †^, Dafni G. Graikioti^2, †^, Cecilia L. Barbieri^3^, Mariana B. Joray^4^, Antonia I. Antoniou^2^, D. Mariano A. Vera^3, ***^,** [**Constantinos M. Athanassopoulos**](https://www.ncbi.nlm.nih.gov/pubmed/?term=Athanassopoulos%20CM%5BAuthor%5D&cauthor=true&cauthor_uid=32435406)**^2, **^, María C. Carpinella^4, *^**

^1^ Fine Chemical and Natural Products Laboratory, IRNASUS CONICET-UCC, Universidad Católica de Córdoba, Córdoba, Argentina.

^2^ Synthetic Organic Chemistry Laboratory, Department of Chemistry, University of Patras, GR-26504, Patras, Greece.

^3^ Department of Chemistry and Biochemistry, College of Exact and Natural Sciences, Universidad Nacional de Mar del Plata - QUIAMM – INBIOTEC CONICET, Mar del Plata, Argentina

^4^ Fine Chemical and Natural Products Laboratory, IRNASUS CONICET-UCC and *CIDIE CONICET-UCC, Universidad Católica de Córdoba, Córdoba, Argentina.*

* Corresponding author. Email: [ceciliacarpinella@ucc.edu.ar](mailto:ceciliacarpinella@ucc.edu.ar) (M.C. Carpinella).

** Corresponding author. Email: [kath@upatras.gr](mailto:kath@upatras.gr) ([C. M. Athanassopoulos](https://www.ncbi.nlm.nih.gov/pubmed/?term=Athanassopoulos%20CM%5BAuthor%5D&cauthor=true&cauthor_uid=32435406)).

*** Corresponding author. Email: [dmavera@yahoo.com](mailto:dmavera@yahoo.com) (D.M.A. Vera).

^†^ Equal first author contribution.

**Table of Contents**

[**General procedure for the synthesis of phenylethylamines** 5](#_Toc141208842)

[**Synthesis of nitrostyrenes** 5](#_Toc141208843)

[**Synthesis of phenylethylamines** 5](#_Toc141208844)

[**Molecular modeling supplementary figures** 7](#_Toc141208845)

[**Fig. S1.** Superimposition of the modes of binding of compounds **3a**, **6g** and the reference chemotherapeutics vincristine and doxorubicin (Dox) as a yellow translucent surface.. 8](#_Toc141208846)

[**Fig. S2.** Same as Fig. S1 for compound **6i**. 9](#_Toc141208847)

[**Fig. S3.** Various active subject compounds superimposed to vincristine. 10](#_Toc141208848)

[**Fig. S4.** A) hydrophobicity surface B) Frontal cortex showing compound **6g** and C) frontal cortex showing compound **6i**. 11](#_Toc141208849)

[**Fig. S5.** Same as in main text Fig. 8. A-B) Doxorubicin and tariquidar simulations, respectively reproduced from Laiolo et al. [1] and C) compound **6m**. 12](#_Toc141208850)

[**Fig. S6.** RMSd analyses of two trajectories for the complex P-gp/compound **6g**. 13](#_Toc141208851)

[**Fig. S7.** H-bond monitored and averaged for compounds **6k**, **6e**, **4**, **6i** and **6g**. 14](#_Toc141208852)

[**Experimental NMR and HPLC spectra** 15](#_Toc141208853)

[**Fig. S8.** ^1^H-NMR spectrum of compound **3a.** 16](#_Toc141208854)

[**Fig. S9.** ^13^C-NMR spectrum of compound **3a.** 16](#_Toc141208855)

[**Fig. S10.** ^1^H-NMR spectrum of compound **3b.** 17](#_Toc141208856)

[**Fig. S11**. ^13^C-NMR spectrum of compound **3b.** 17](#_Toc141208857)

[**Fig. S12.** ^1^H-NMR spectrum of compound **3c.** 18](#_Toc141208858)

[**Fig. S13.** ^13^C-NMR spectrum of compound **3c.** 18](#_Toc141208859)

[**Fig. S14.** ^1^H-NMR spectrum of compound **3d.** 19](#_Toc141208860)

[**Fig. S15.** ^13^C-NMR spectrum of compound **3d.** 19](#_Toc141208861)

[**Fig. S16.** ^1^H-NMR spectrum of compound **3e.** 20](#_Toc141208862)

[**Fig. S17.** ^13^C-NMR spectrum of compound **3e.** 20](#_Toc141208863)

[**Fig. S18.** ^1^H-NMR spectrum of compound **4.** 21](#_Toc141208864)

[**Fig. S19.** ^13^C-NMR spectrum of compound **4.** 21](#_Toc141208865)

[**Fig. S20.** ^1^H-NMR spectrum of compound **6a.** 22](#_Toc141208866)

[**Fig. S21.** ^13^C-NMR spectrum of compound **6a.** 22](#_Toc141208867)

[**Fig. S22.** ^1^H-NMR spectrum of compound **6b.** 23](#_Toc141208868)

[**Fig. S23.** ^13^C-NMR spectrum of compound **6b.** 23](#_Toc141208869)

[**Fig. S24.** ^1^H-NMR spectrum of compound **6c.** 24](#_Toc141208870)

[**Fig. S25.** ^13^C-NMR spectrum of compound **6c.** 24](#_Toc141208871)

[**Fig. S26.** ^1^H-NMR spectrum of compound **6d.** 25](#_Toc141208872)

[**Fig. S27.** ^13^C-NMR spectrum of compound **6d.** 25](#_Toc141208873)

[**Fig. S28.** ^1^H-NMR spectrum of compound **6e.** 26](#_Toc141208874)

[**Fig. S29.** ^13^C-NMR spectrum of compound **6e.** 26](#_Toc141208875)

[**Fig. S30.** ^1^H-NMR spectrum of compound **6f.** 27](#_Toc141208876)

[**Fig. S31.** ^13^C-NMR spectrum of compound **6f.** 27](#_Toc141208877)

[**Fig. S32.** ^1^H-NMR spectrum of compound **6g.** 28](#_Toc141208878)

[**Fig. S33.** ^13^C-NMR spectrum of compound **6g.** 28](#_Toc141208879)

[**Fig. S34.** ^1^H-NMR spectrum of compound **6h.** 29](#_Toc141208880)

[**Fig. S35.** ^13^C-NMR spectrum of compound **6h.** 29](#_Toc141208881)

[**Fig. S36.** ^1^H-NMR spectrum of compound **6i.** 30](#_Toc141208882)

[**Fig. S37.** ^13^C-NMR spectrum of compound **6i.** 30](#_Toc141208883)

[**Fig. S38.** ^1^H-NMR spectrum of compound **6j.** 31](#_Toc141208884)

[**Fig. S39.** ^13^C-NMR spectrum of compound **6j.** 31](#_Toc141208885)

[**Fig. S40.** ^1^H-NMR spectrum of compound **6l.** 32](#_Toc141208886)

[**Fig. S41.** ^13^C-NMR spectrum of compound **6l.** 32](#_Toc141208887)

[**Fig. S42.** ^1^H-NMR spectrum of compound **6m.** 33](#_Toc141208888)

[**Fig. S43.** ^13^C-NMR spectrum of compound **6m.** 33](#_Toc141208889)

[**Fig. S44.** HPLC chromatogram of compound **3a.** 34](#_Toc141208890)

[**Fig. S45.** HPLC chromatogram of compound **3b.** 34](#_Toc141208891)

[**Fig. S46.** HPLC chromatogram of compound **3c.** 35](#_Toc141208892)

[**Fig. S47.** HPLC chromatogram of compound **3d.** 35](#_Toc141208893)

[**Fig. S48.** HPLC chromatogram of compound **3e.** 36](#_Toc141208894)

[**Fig. S49.** HPLC chromatogram of compound **4.** 36](#_Toc141208895)

[**Fig. S50.** HPLC chromatogram of compound **6a.** 37](#_Toc141208896)

[**Fig. S51.** HPLC chromatogram of compound **6b.** 37](#_Toc141208897)

[**Fig. S52.** HPLC chromatogram of compound **6c.** 38](#_Toc141208898)

[**Fig. S53.** HPLC chromatogram of compound **6d.** 38](#_Toc141208899)

[**Fig. S54.** HPLC chromatogram of compound **6e.** 39](#_Toc141208900)

[**Fig. S55.** HPLC chromatogram of compound **6f.** 39](#_Toc141208901)

[**Fig. S56.** HPLC chromatogram of compound **6g.** 40](#_Toc141208902)

[**Fig. S57.** HPLC chromatogram of compound **6h.** 40](#_Toc141208903)

[**Fig. S58.** HPLC chromatogram of compound **6i.** 41](#_Toc141208904)

[**Fig. S59.** HPLC chromatogram of compound **6j.** 41](#_Toc141208905)

[**Fig. S60.** HPLC chromatogram of compound **6k.** 42](#_Toc141208906)

[**Fig. S61.** HPLC chromatogram of compound **6l.** 42](#_Toc141208907)

[**Fig. S62.** HPLC chromatogram of compound **6m.** 43](#_Toc141208908)

[**Cytotoxic activity of the compounds 4, 6h, 6i, 6k, 6l and tariquidar** 44](#_Toc141208909)

[**Fig. S63.** Antiproliferative effect of the derivatives **4**, **6h**, **6i**, **6k**, and **6l** and of tariquidar against multidrug-resistant Lucena 1 cells. 44](#_Toc141208910)

[**Fig. S64.** Antiproliferative effect of the derivatives **4**, **6h**, **6i**, **6k**, and **6l** and of tariquidar against sensitive K562 cells. 45](#_Toc141208911)

**General procedure for the synthesis of phenylethylamines**

**Synthesis of nitrostyrenes**

In a round bottom flask glacial acetic acid (14.6 mL) and ammonium acetate (41.6 mmol) were added and the suspension was stirred until dissolution of the white solid. Then, nitromethane (61.4 mmol) and the corresponding benzaldehyde (10.2 mmol) were added, and the mixture was heated under reflux for 3 h. After completion of the reaction the mixture was condensed to dryness and the residue was subjected to FCC, affording the corresponding nitrostyrenes as yellow solids.

*3,4-Dimethoxynitrostyrene*

Yield: 85%; R_f_ (PhMe/AcOEt 8:2): 0.51; m.p.=140-140.8 ^ο^C; ^1^H NMR (600 MHz, CDCl_3_) *δ* 8.20 (d, *J=*15.8 Hz, 1 H), 8.05 (d, *J*=16 Hz, 1 H), 7.43-7.46 (m, 1 H), 7.30 (br s, 1 H), 6.77-6.80 (m, 1 H), 3.84 (s, 6 H); ^13^C NMR (151 MHz, CDCl_3_) *δ* 150.3, 149.4, 139.2, 138.7, 124.4, 124.0, 113.5, 112.6, 55.9, 55.9.

*3,4,5-Trimethoxynitrostyrene*

Yield: 82%; R_f_ (PhMe): 0.28; m.p.: 120-121 ^ο^C; ^1^H NMR (600 MHz, CDCl_3_) *δ* 8.20 (d, *J=*16 Hz, 1 H), 8.05 (d, *J*=16.2 Hz, 1 H), 7.06 (s, 2 H), 3.89 (s, 6 H), 3.88 (s, 3 H); ^13^C NMR (151 MHz, CDCl_3_) *δ* 153.6, 140.3, 138.2, 134.6, 124.9, 106.9, 60.8, 56.2.

**Synthesis of phenylethylamines**

A solution of the corresponding nitrostyrene (2.93 mmol) in ethanol (EtOH) (28 mL) and 6 M HCl_(aq)_ (6 mmol) was subjected to hydrogenation over 10% Pd/C (220 mg) at ambient temperature and pressure. After 48 h filtration of the reaction mixture through Celite^®^, removal of the solvent under reduced pressure and FCC purification were received the corresponding phenylethylamines as pale-yellow oils.

*3,4-Dimethoxyphenylethylamine*

Yield: 70%; R_f_ (DCM/MeOH/NH_3_ 8:2:0.2): 0.30; ^1^H NMR (600 MHz, CDCl_3_) *δ* 6.73-6.76 (m, 2 H), 6.72-6.74 (m, 1 H), 3.88 (s, 6 H), 3.42 (br s, 2 H), 3.04-3.06 (m, 2 H), 2.67-2.69 (m, 2 H); ^13^C NMR (151 MHz, CDCl_3_) *δ* 150.6, 147.9, 132.8, 121.5, 113.7, 112.4, 55.9, 55.8, 43.6, 39.7.

*3,4,5-Trimethoxyphenylethylamine*

Yield: 65%; R_f_ (DCM/MeOH/NH_3_ 9:1:0.1): 0.39; ^1^H NMR (600 MHz, CDCl_3_) *δ* 6.36-6.38 (m, 2 H), 3.90 (s, 6 H), 3.88 (s, 3 H), 3.51 (br s, 2 H), 3.05-3.07 (m, 2 H), 2.67-2.69 (m, 2 H); ^13^C NMR (151 MHz, CDCl_3_) *δ* 152.9, 136.4, 134.8, 105.5, 60.8, 56.2, 40.3, 38.3.

**Molecular modeling supplementary figures**

**
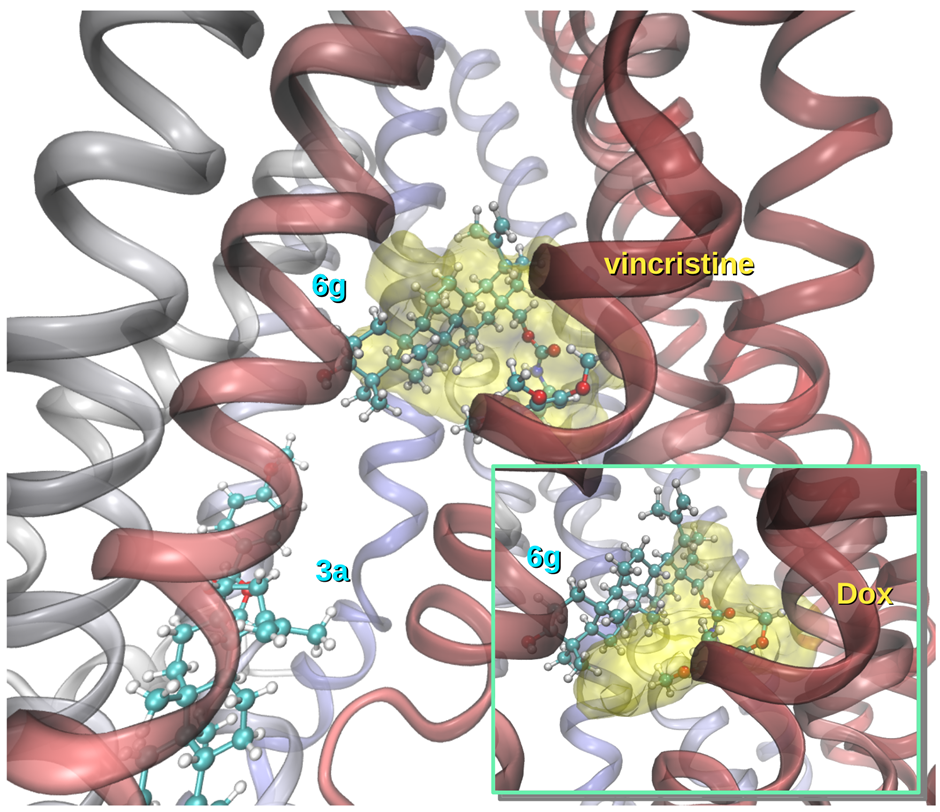
**

**Fig. S1.** Superimposition of the modes of binding of compounds **3a**, **6g** and the reference chemotherapeutics vincristine and doxorubicin (Dox) as a yellow translucent surface. All structures taken from a representative snapshot the most populated cluster of a trajectory; α-helices colored according to the sequence from red to blue.


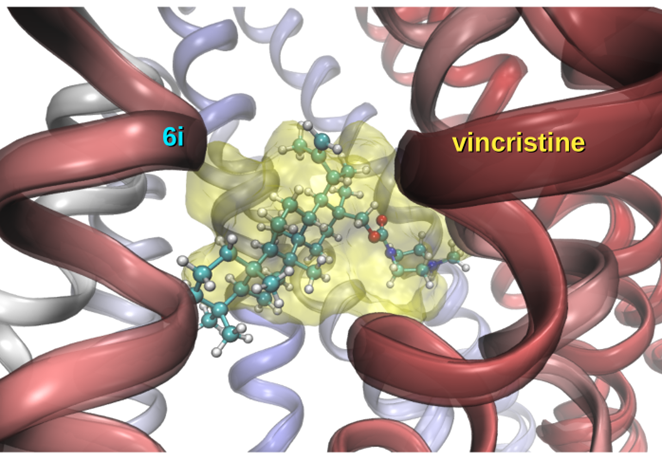


**Fig. S2.** Same as Fig. S1 for compound **6i**.


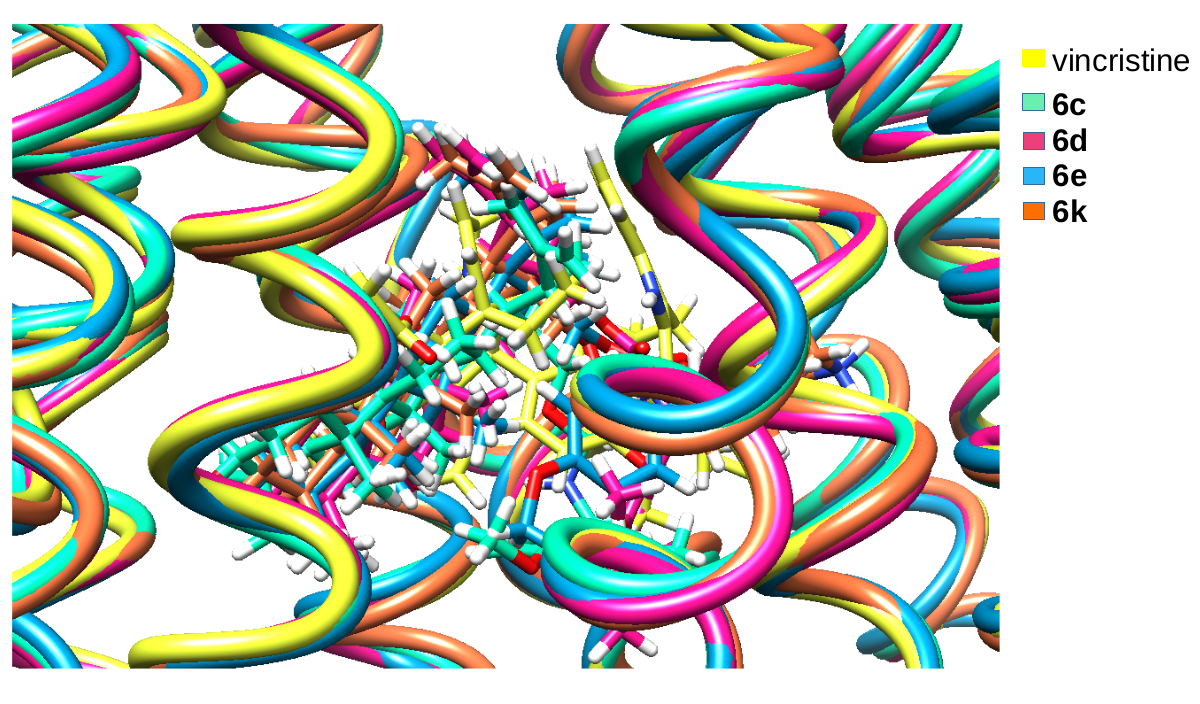


**Fig. S3.** Various active subject compounds superimposed to vincristine. Structures aligned from a representative snapshot of the most populated cluster of each trajectory.


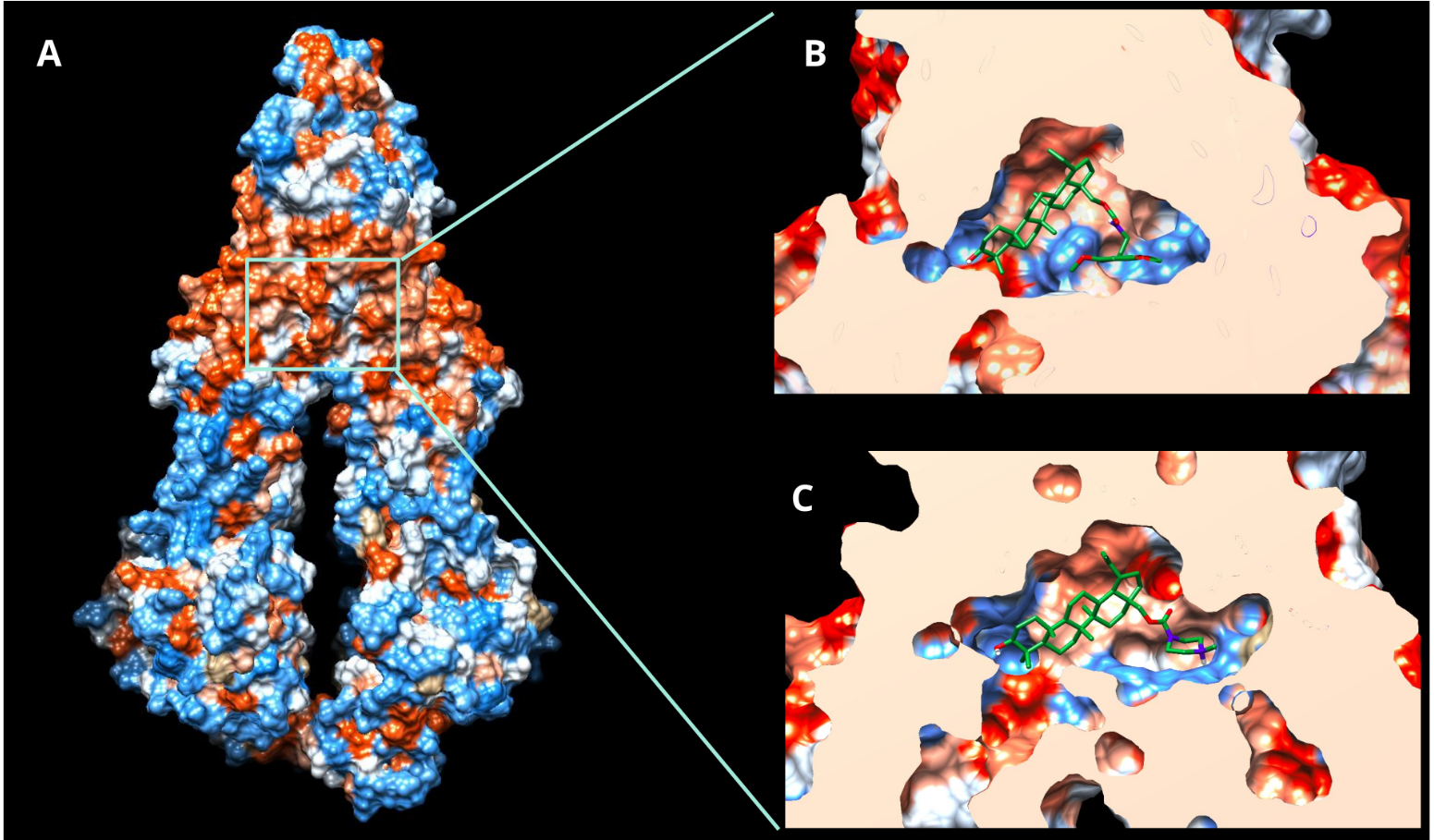


**Fig. S4.** A) hydrophobicity surface (orange = most hydrophobic). B) Frontal cortex showing compound **6g** and C) frontal cortex showing compound **6i**.


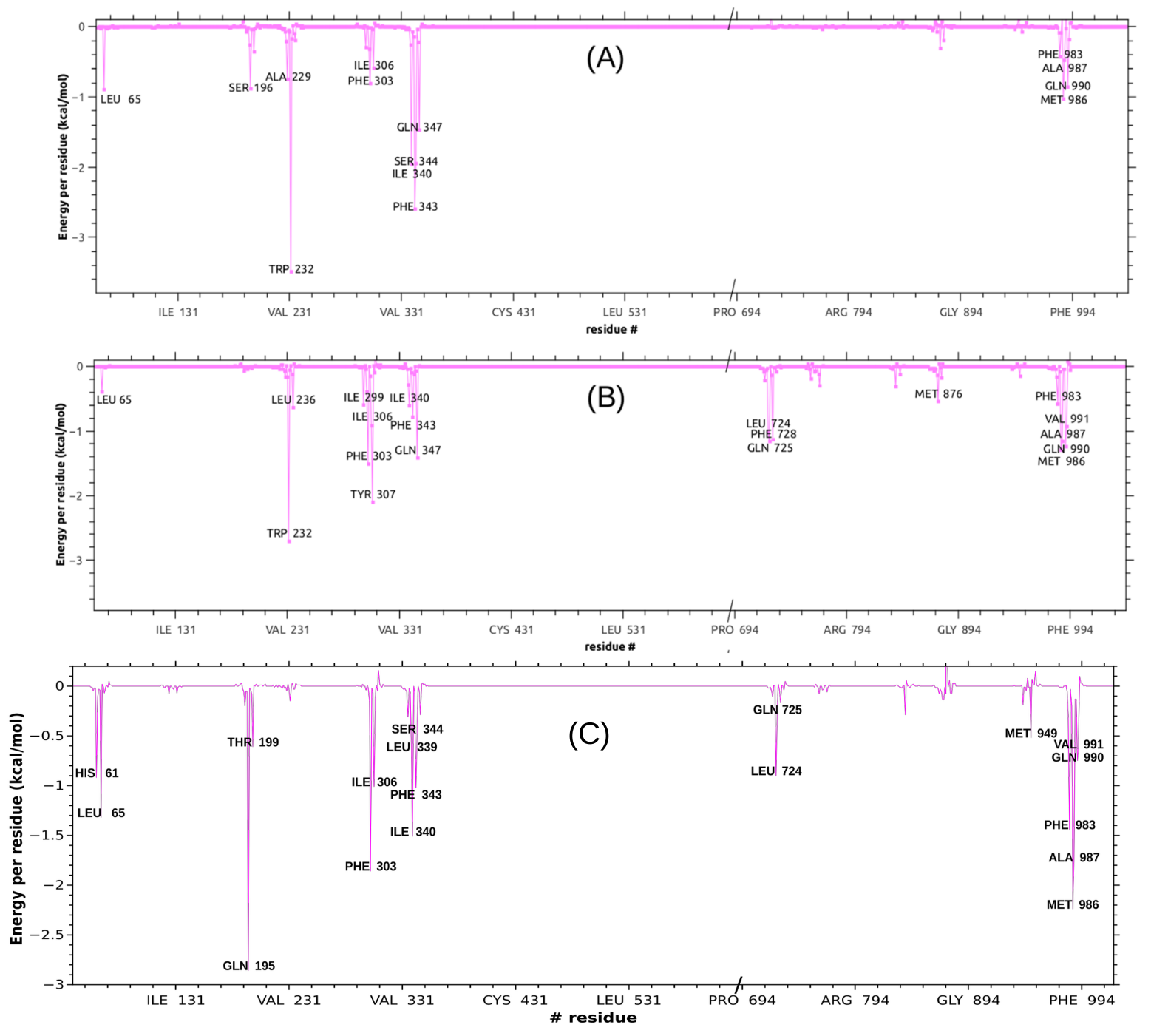


**Fig. S5.** Same as in main text Fig. 8. A-B) Doxorubicin and tariquidar simulations, respectively reproduced from Laiolo et al. [1] and C) compound **6m**.


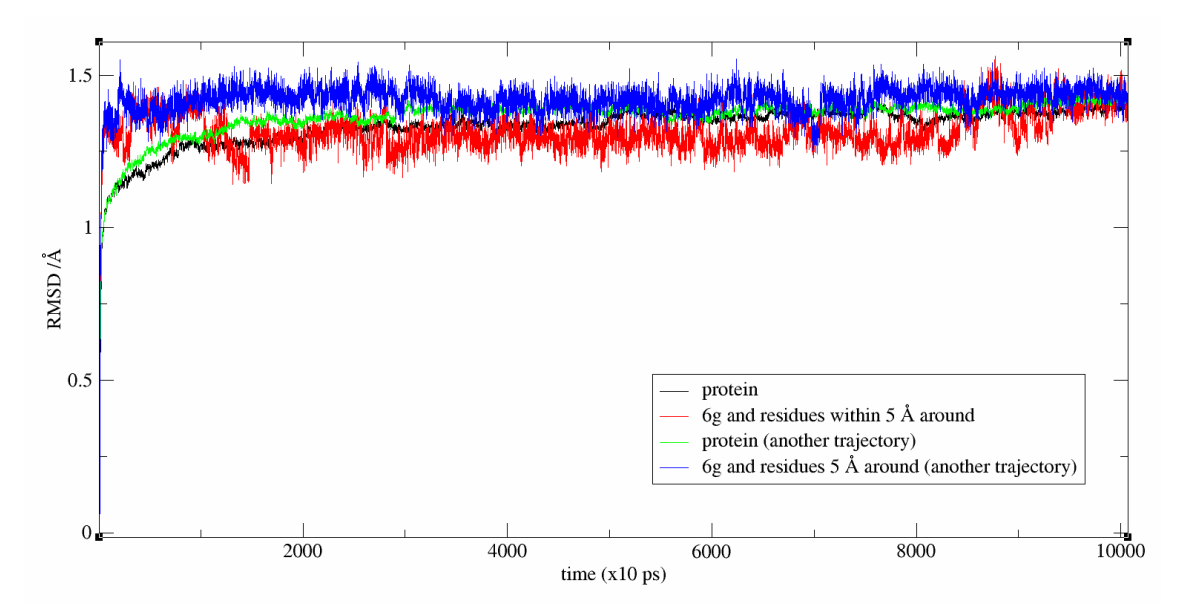


**Fig. S6.** RMSd analyses of two trajectories for the complex P-gp/compound **6g**.


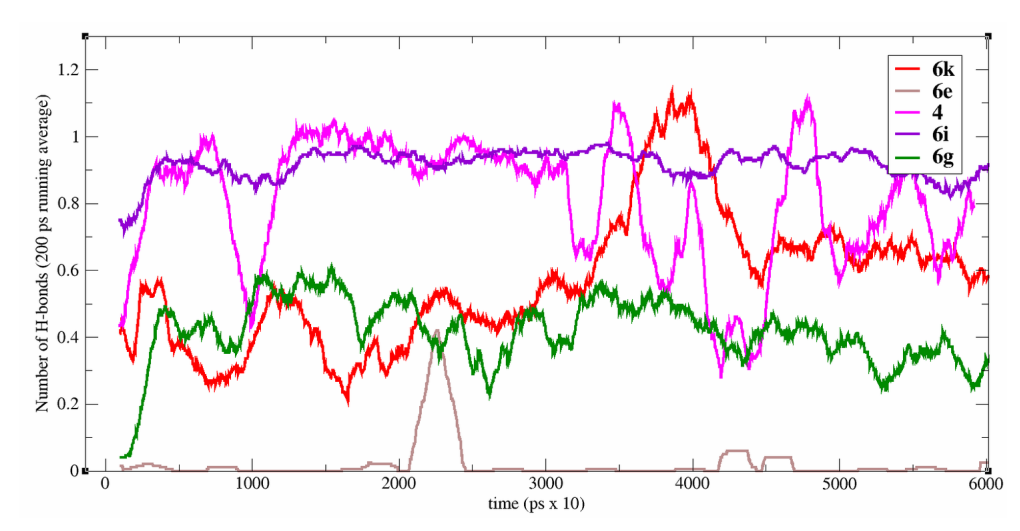


**Fig. S7.** H-bond monitored and averaged (200 ps running average) for compounds **6k**, **6e**, **4**, **6i** and **6g**. Most simulations were between 0.5 and 1.5 total average number of H-bonds.

**Experimental NMR and HPLC spectra**


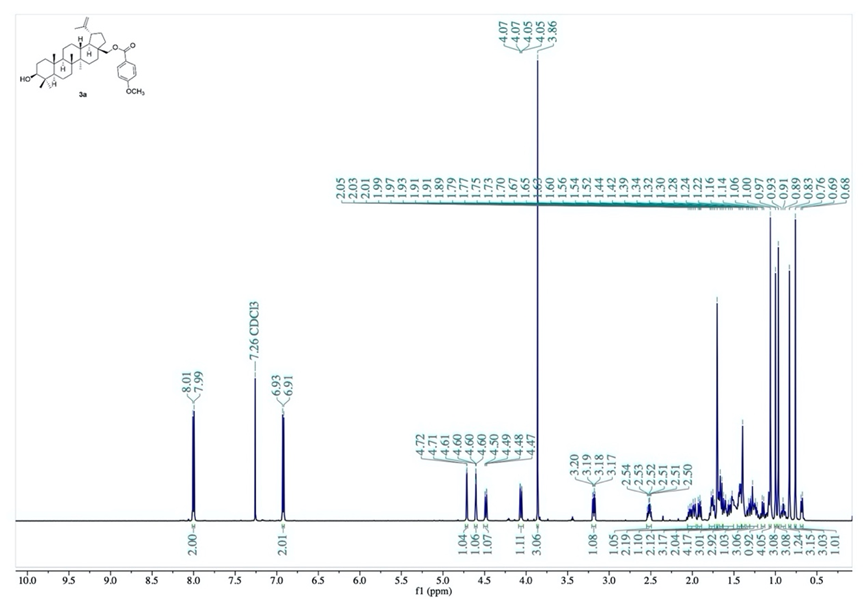


**Fig. S8.** ^1^H-NMR spectrum of compound **3a.**


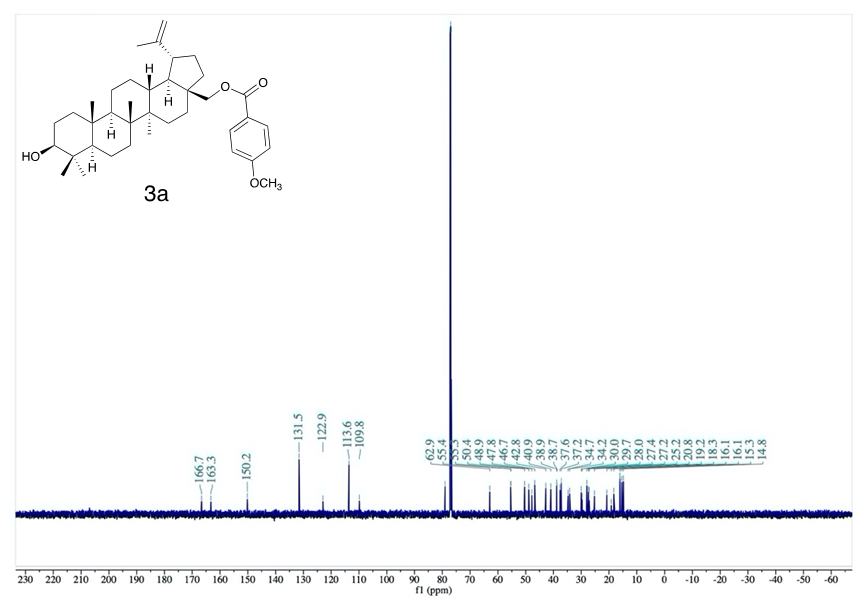


**Fig. S9.** ^13^C-NMR spectrum of compound **3a.**


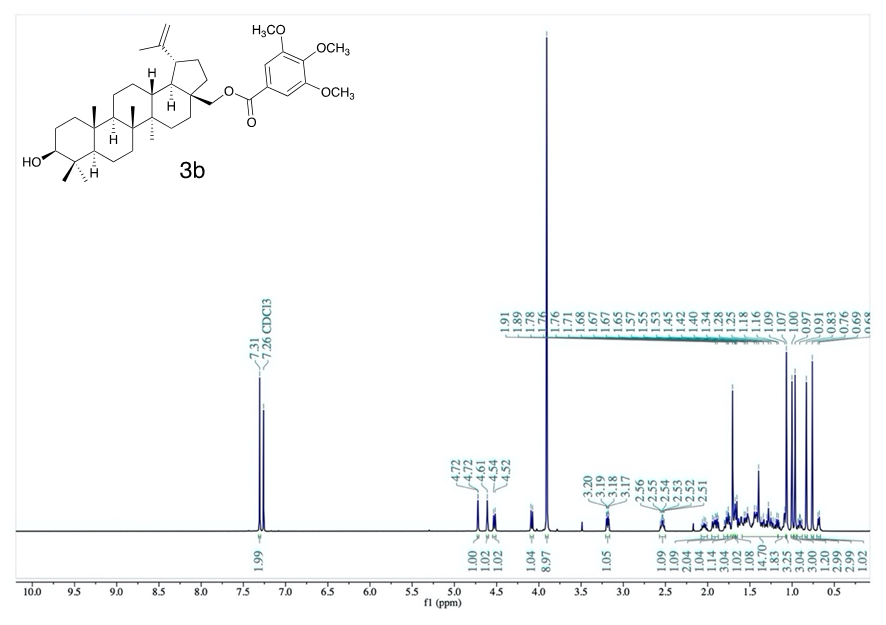


**Fig. S10.** ^1^H-NMR spectrum of compound **3b.**


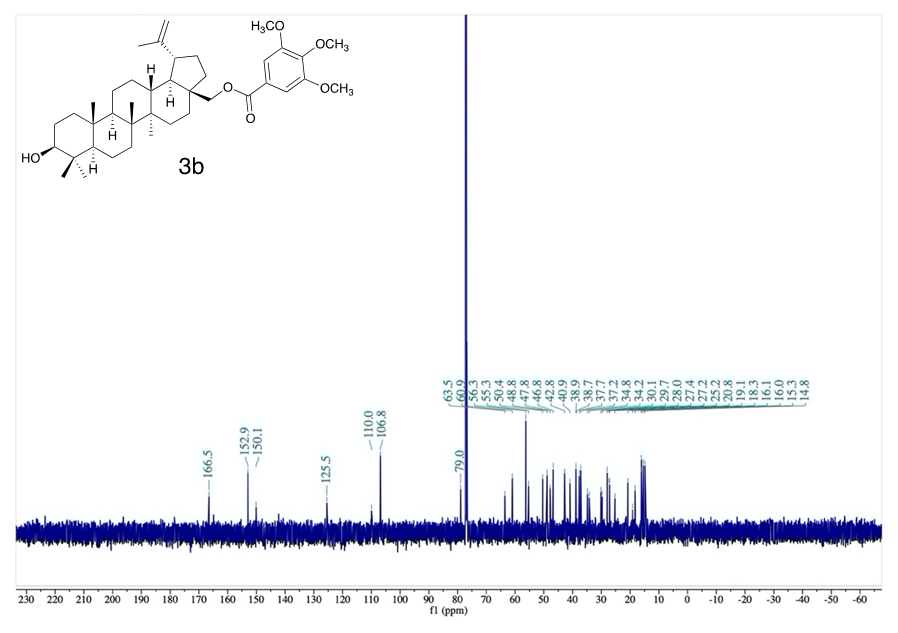


**Fig. S11**. ^13^C-NMR spectrum of compound **3b.**


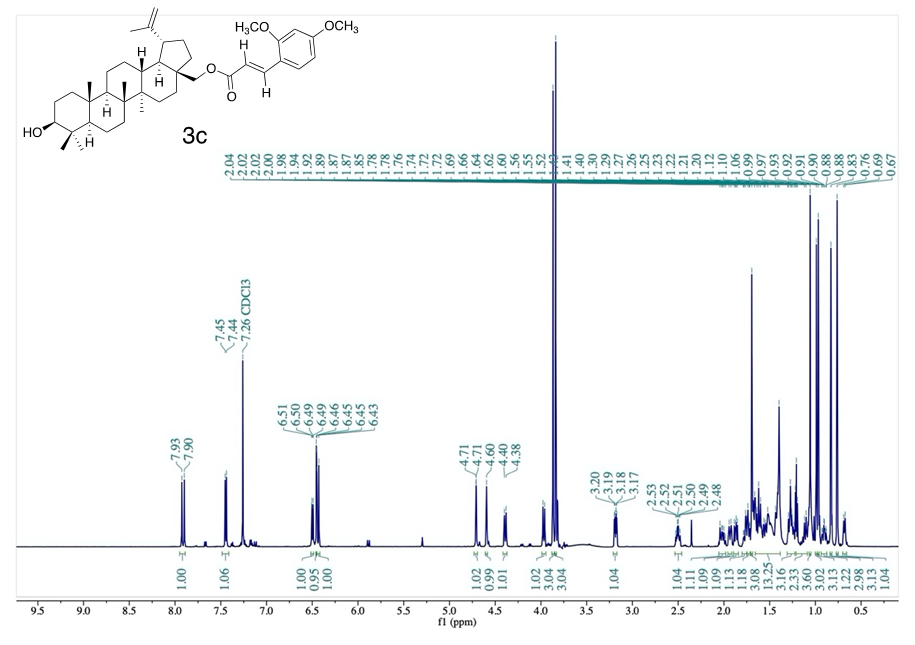


**Fig. S12.** ^1^H-NMR spectrum of compound **3c.**


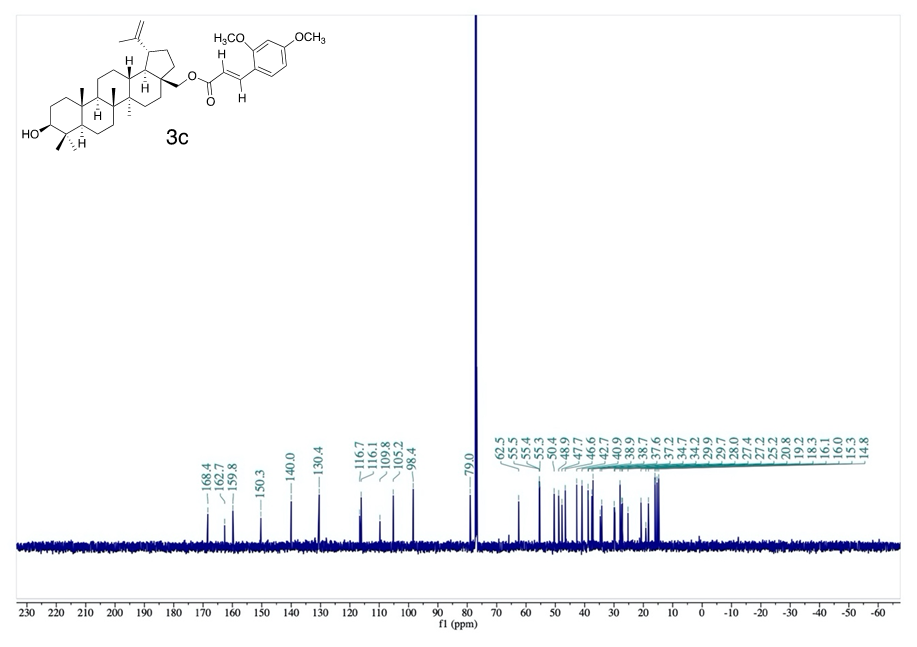


**Fig. S13.** ^13^C-NMR spectrum of compound **3c.**


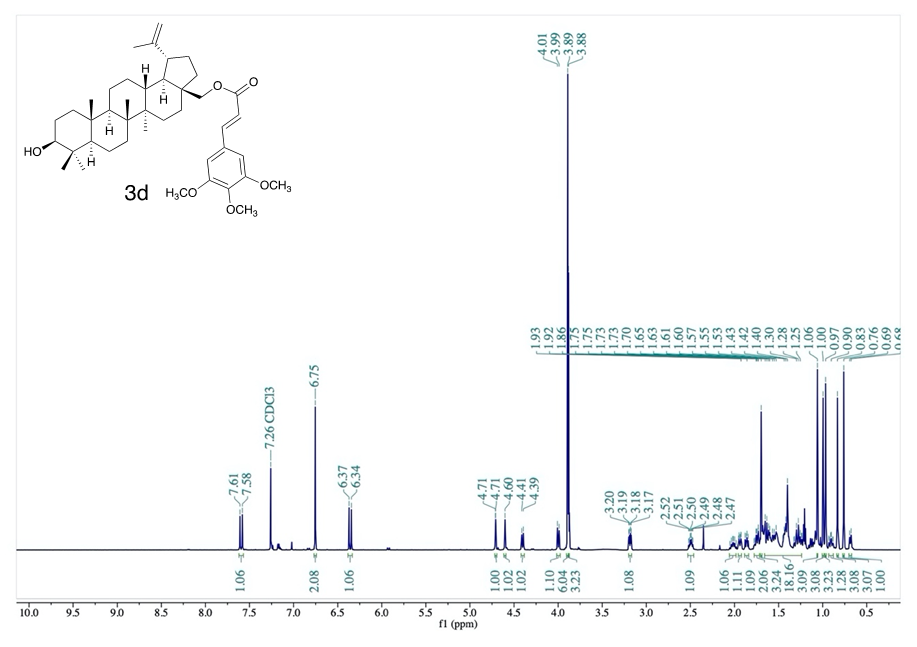


**Fig. S14.** ^1^H-NMR spectrum of compound **3d.**


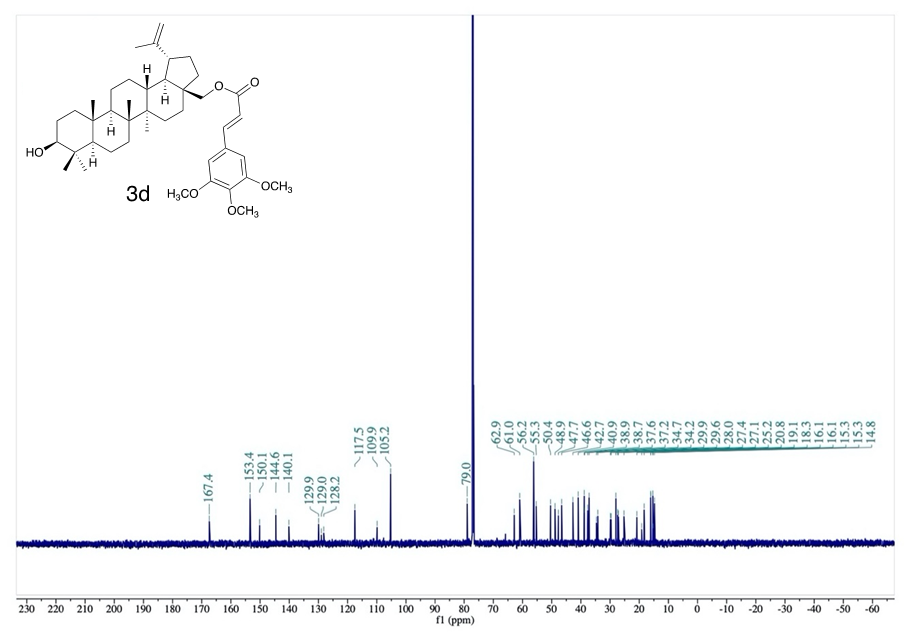


**Fig. S15.** ^13^C-NMR spectrum of compound **3d.**


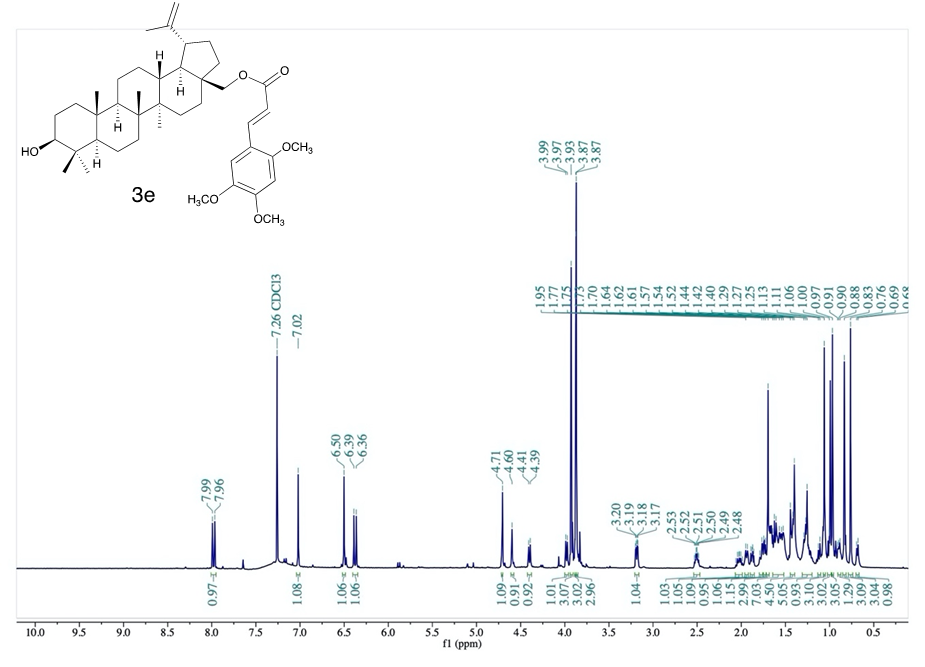


**Fig. S16.** ^1^H-NMR spectrum of compound **3e.**


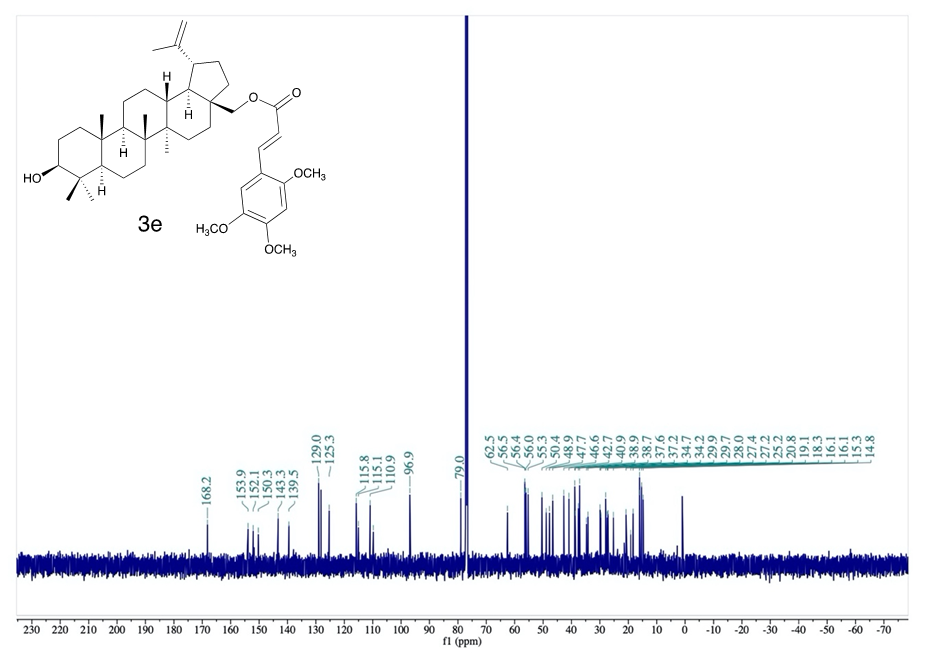


**Fig. S17.** ^13^C-NMR spectrum of compound **3e.**


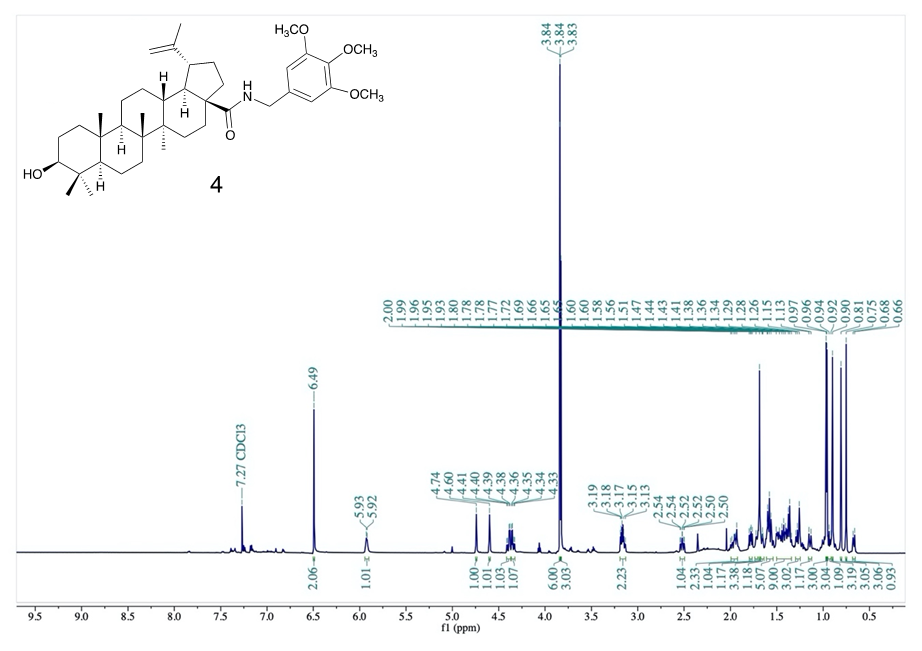


**Fig. S18.** ^1^H-NMR spectrum of compound **4.**


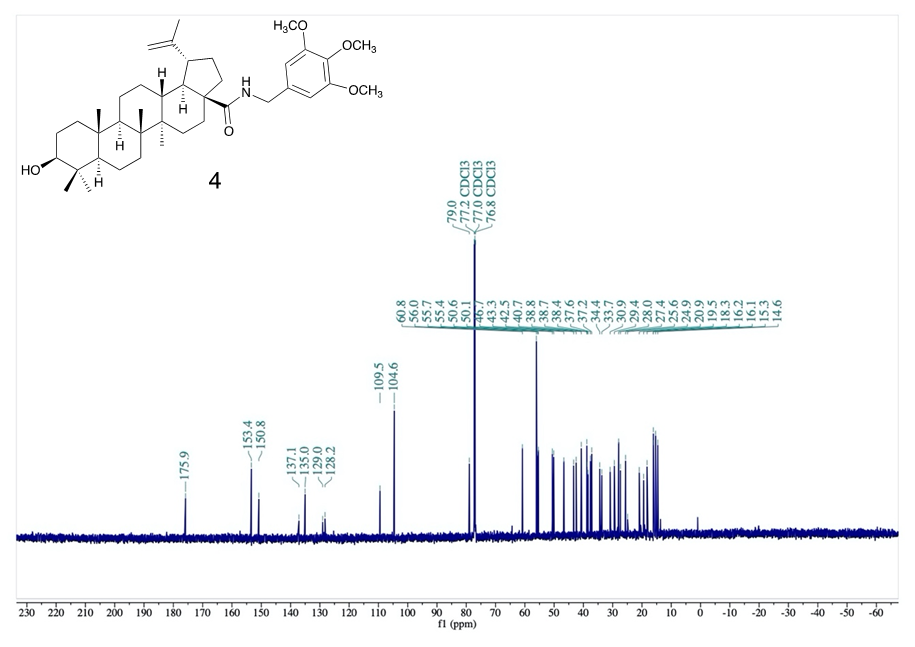


**Fig. S19.** ^13^C-NMR spectrum of compound **4.**


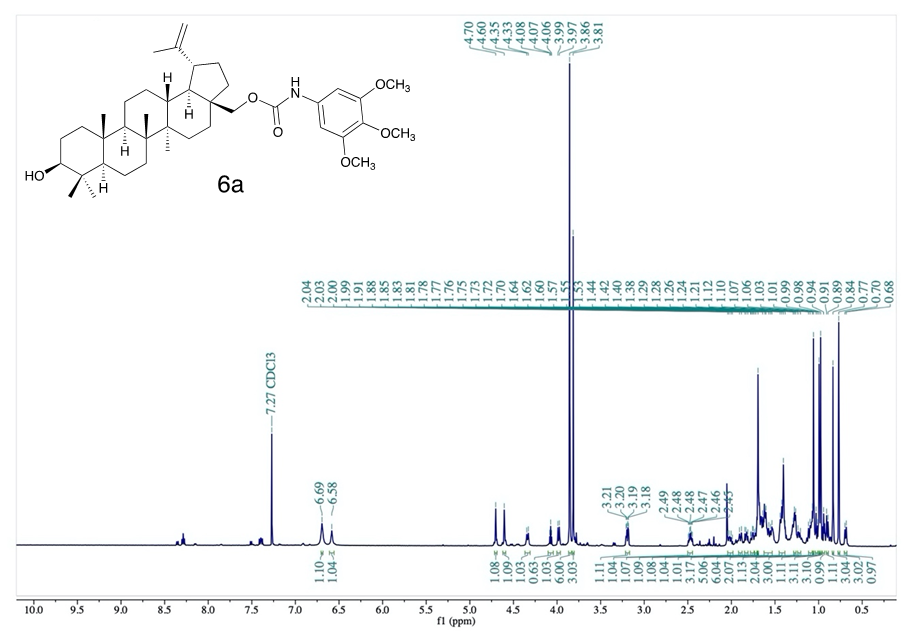


**Fig. S20.** ^1^H-NMR spectrum of compound **6a.**


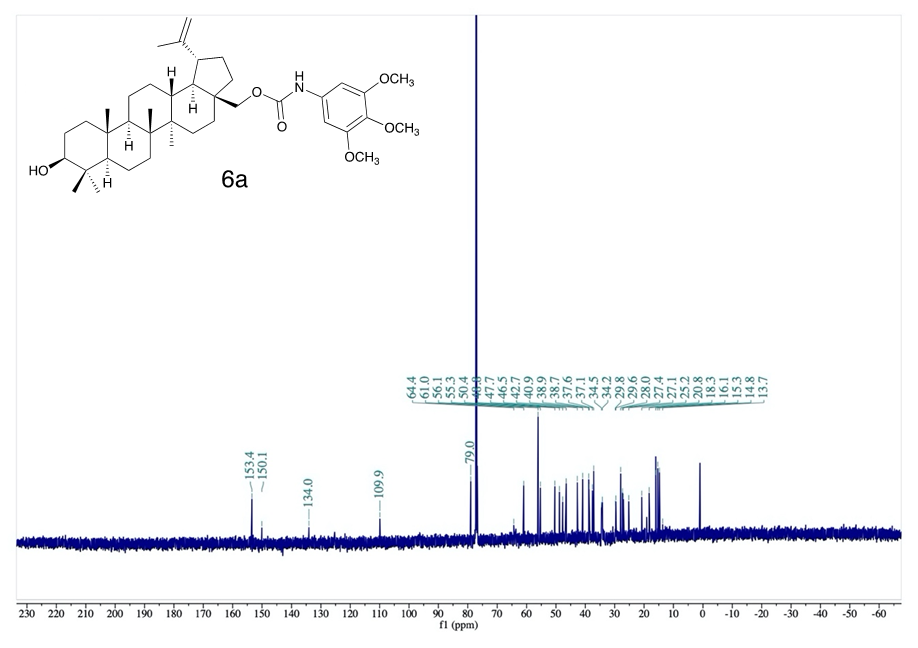


**Fig. S21.** ^13^C-NMR spectrum of compound **6a.**


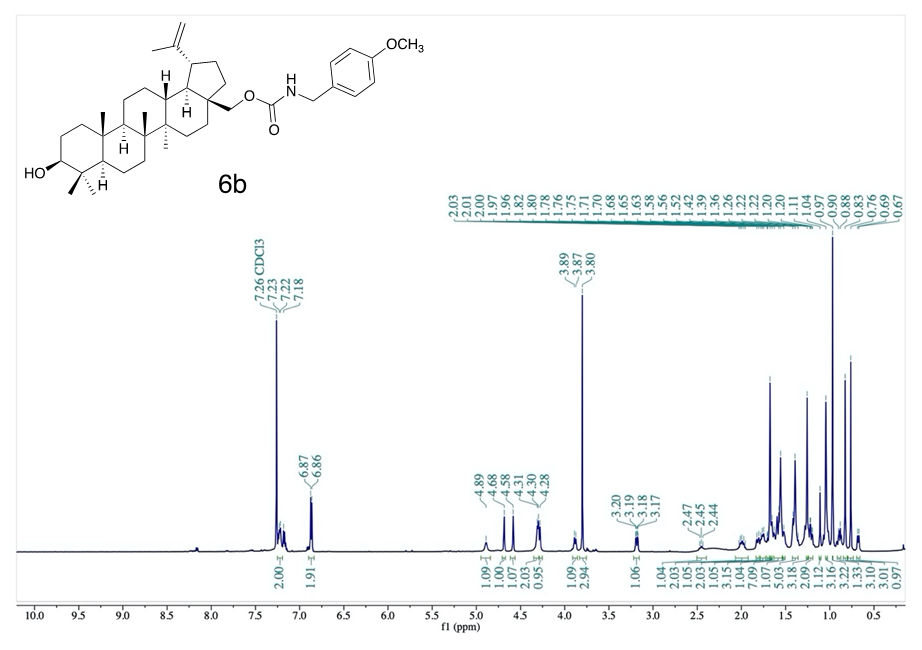


**Fig. S22.** ^1^H-NMR spectrum of compound **6b.**


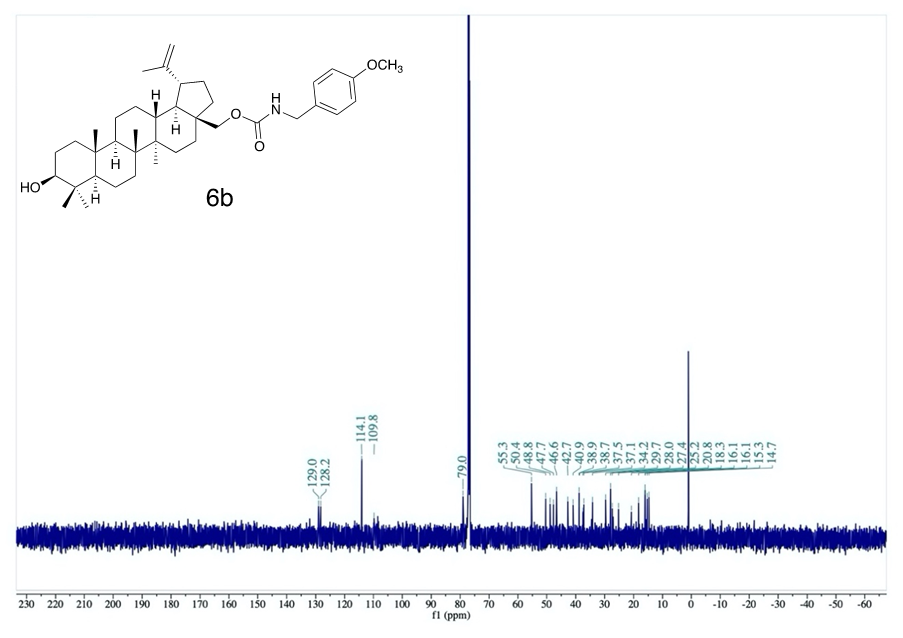


**Fig. S23.** ^13^C-NMR spectrum of compound **6b.**


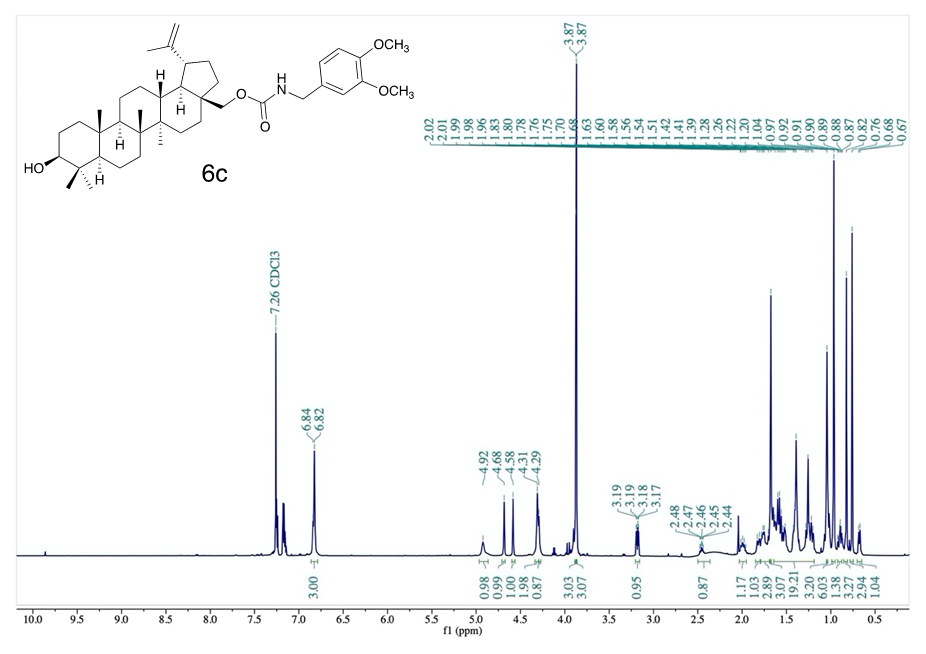


**Fig. S24.** ^1^H-NMR spectrum of compound **6c.**


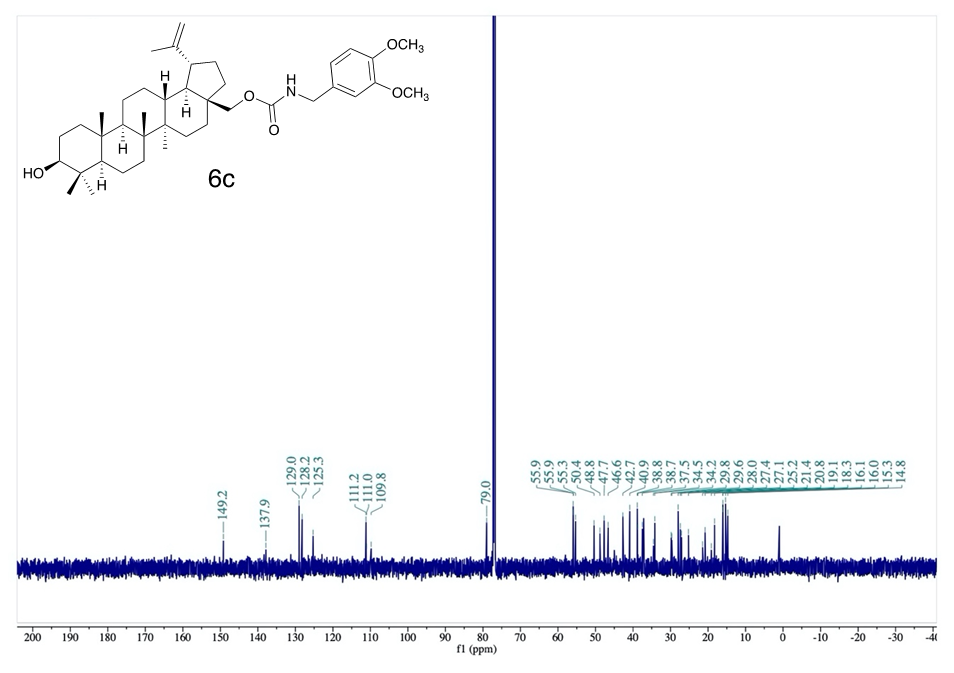


**Fig. S25.** ^13^C-NMR spectrum of compound **6c.**


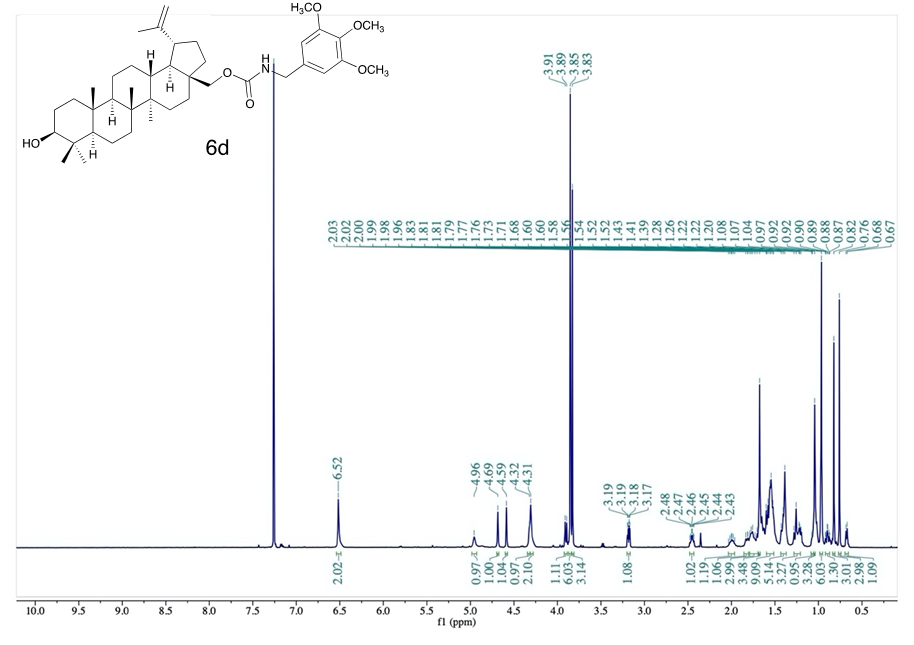


**Fig. S26.** ^1^H-NMR spectrum of compound **6d.**


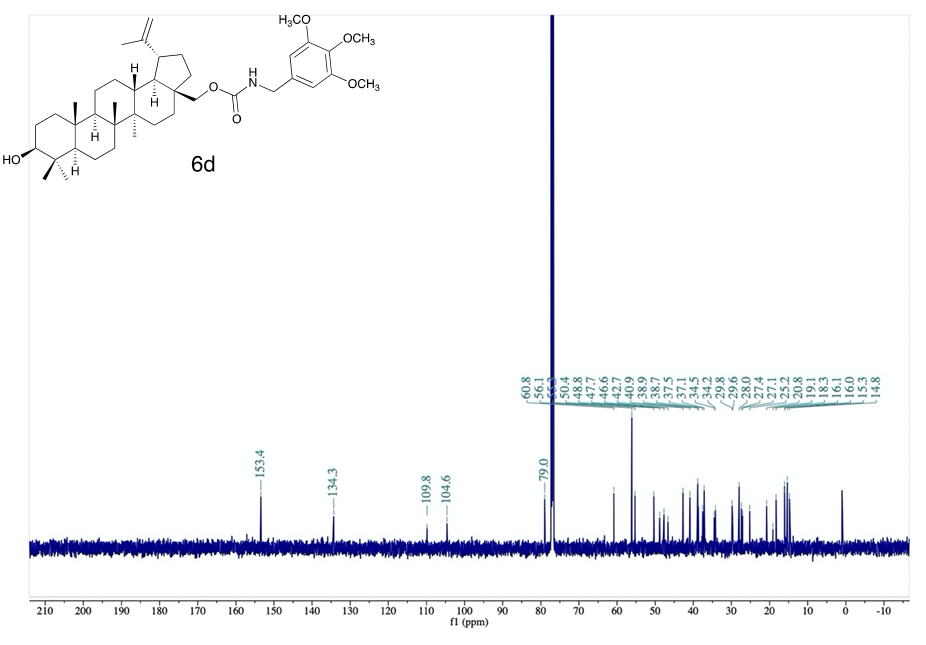


**Fig. S27.** ^13^C-NMR spectrum of compound **6d.**


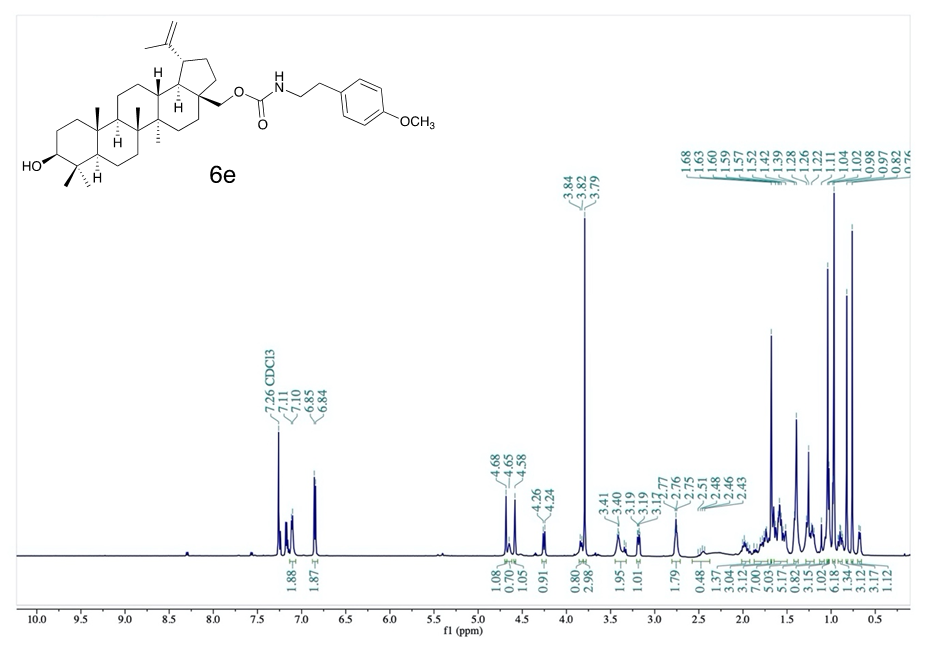


**Fig. S28.** ^1^H-NMR spectrum of compound **6e.**


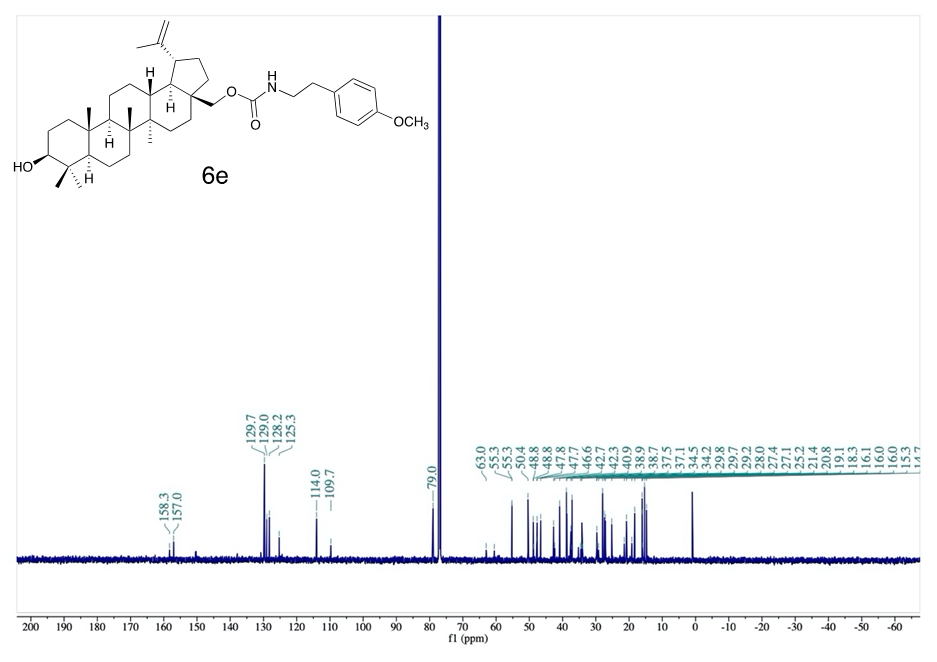


**Fig. S29.** ^13^C-NMR spectrum of compound **6e.**


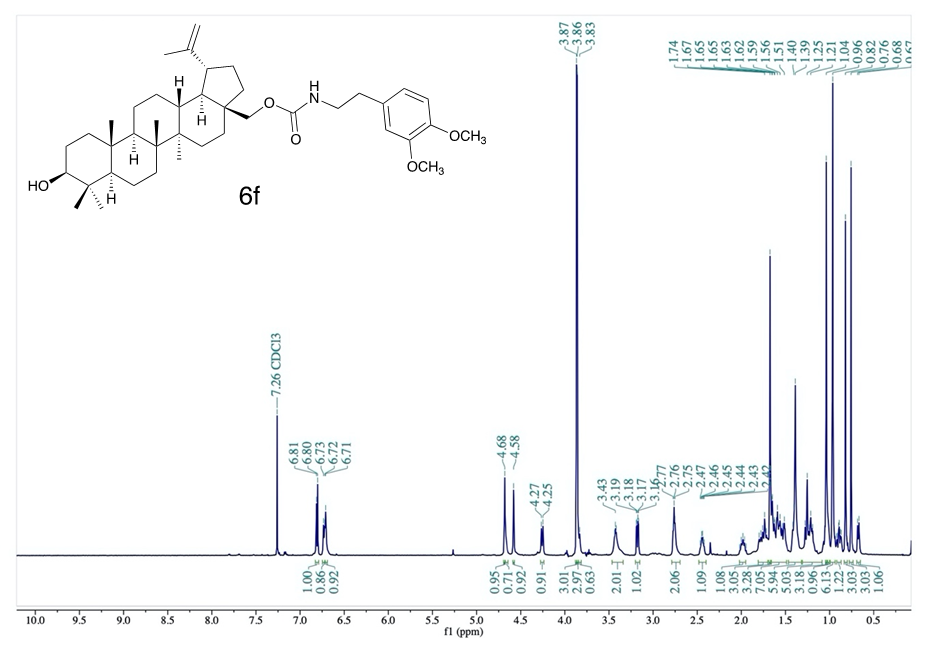


**Fig. S30.** ^1^H-NMR spectrum of compound **6f.**


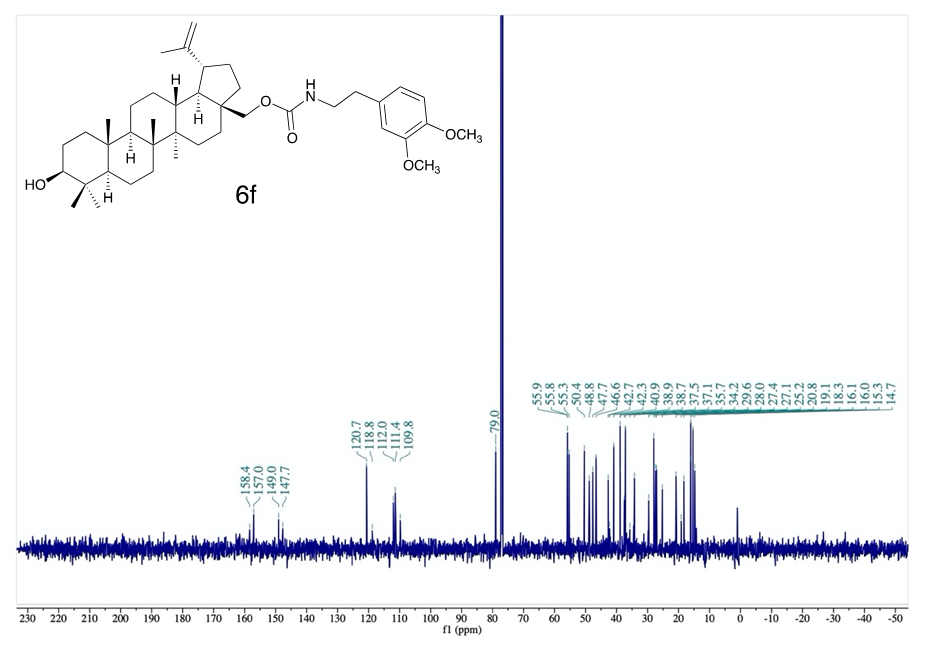


**Fig. S31.** ^13^C-NMR spectrum of compound **6f.**


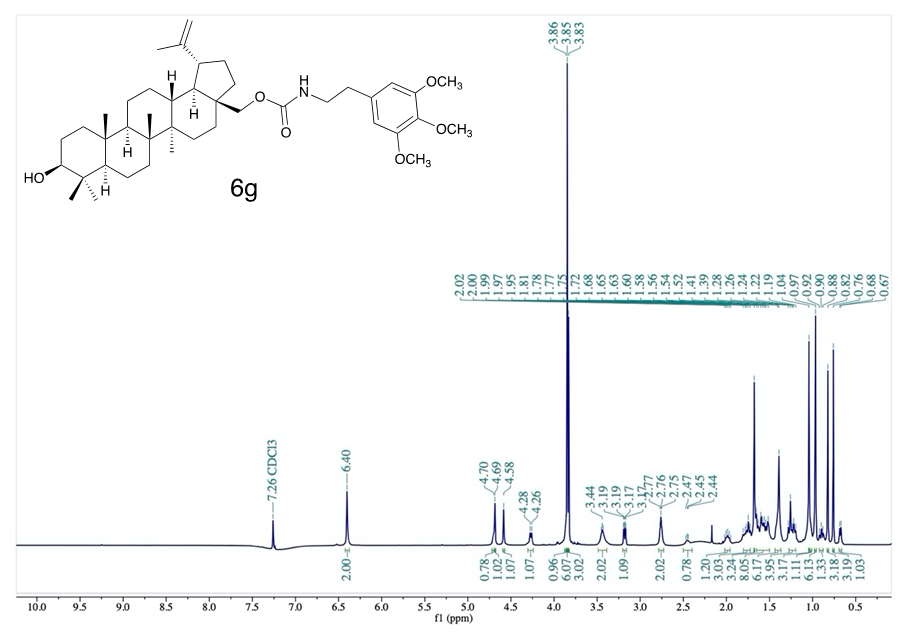


**Fig. S32.** ^1^H-NMR spectrum of compound **6g.**


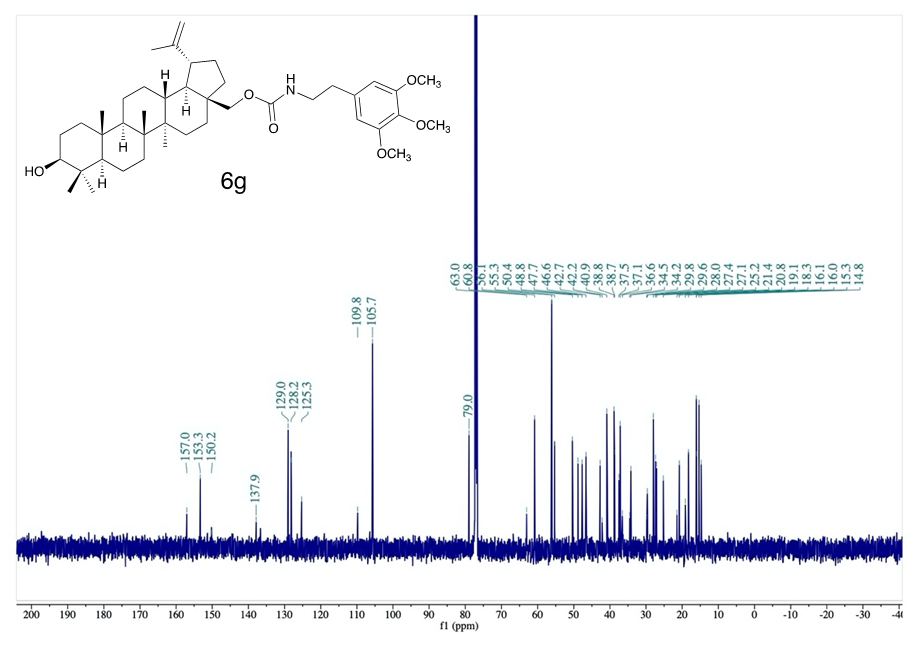


**Fig. S33.** ^13^C-NMR spectrum of compound **6g.**


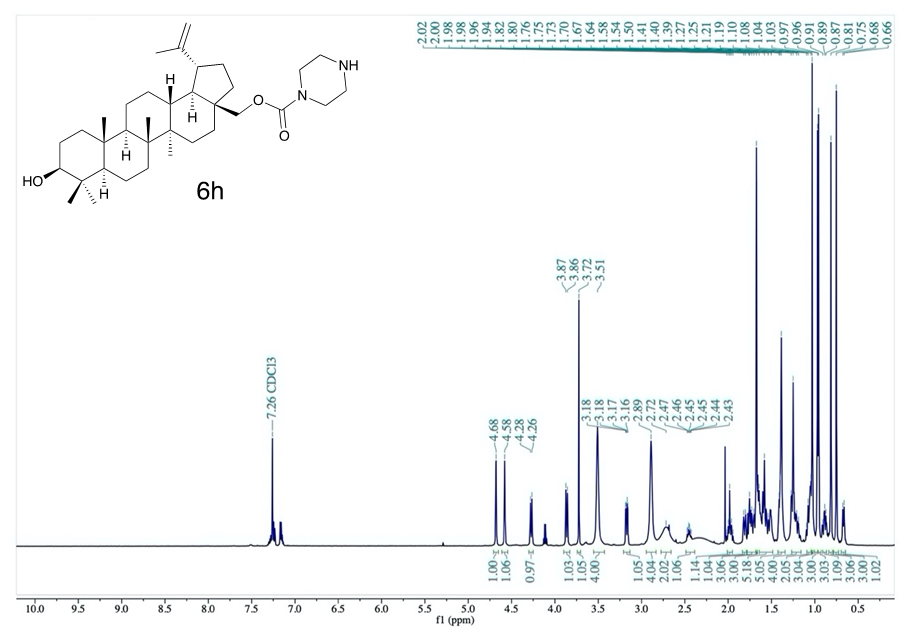


**Fig. S34.** ^1^H-NMR spectrum of compound **6h.**


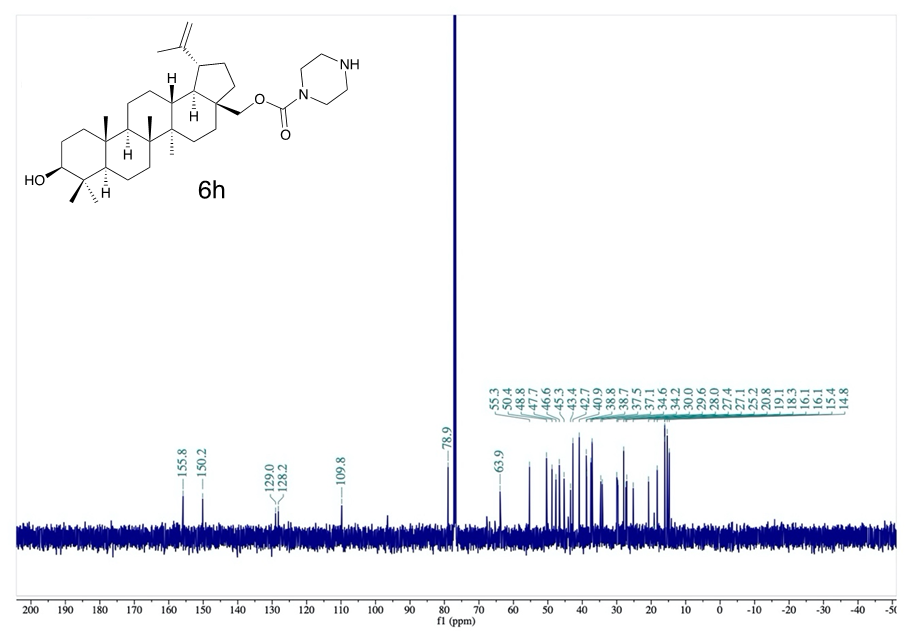


**Fig. S35.** ^13^C-NMR spectrum of compound **6h.**


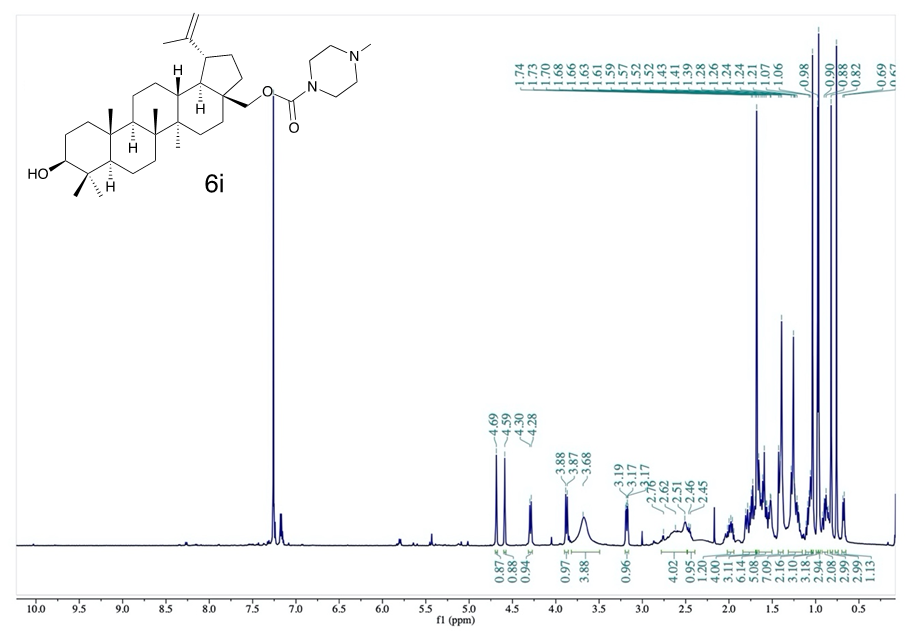


**Fig. S36.** ^1^H-NMR spectrum of compound **6i.**


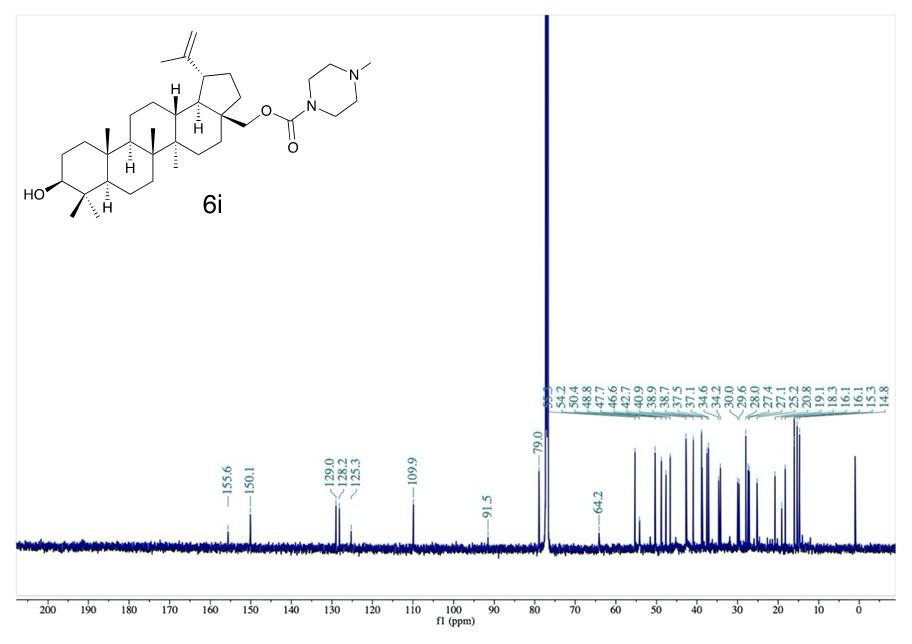


**Fig. S37.** ^13^C-NMR spectrum of compound **6i.**


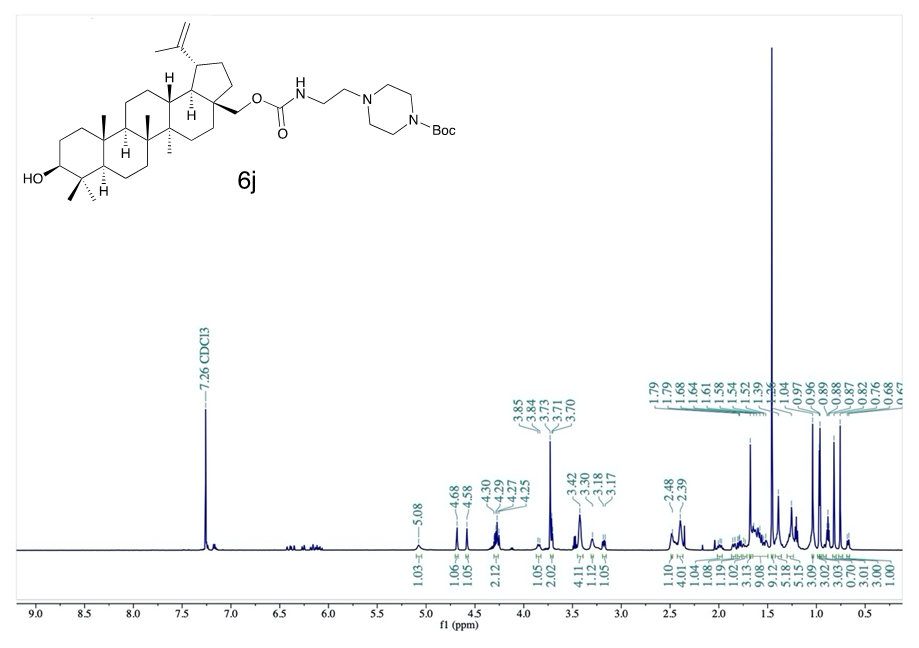


**Fig. S38.** ^1^H-NMR spectrum of compound **6j.**


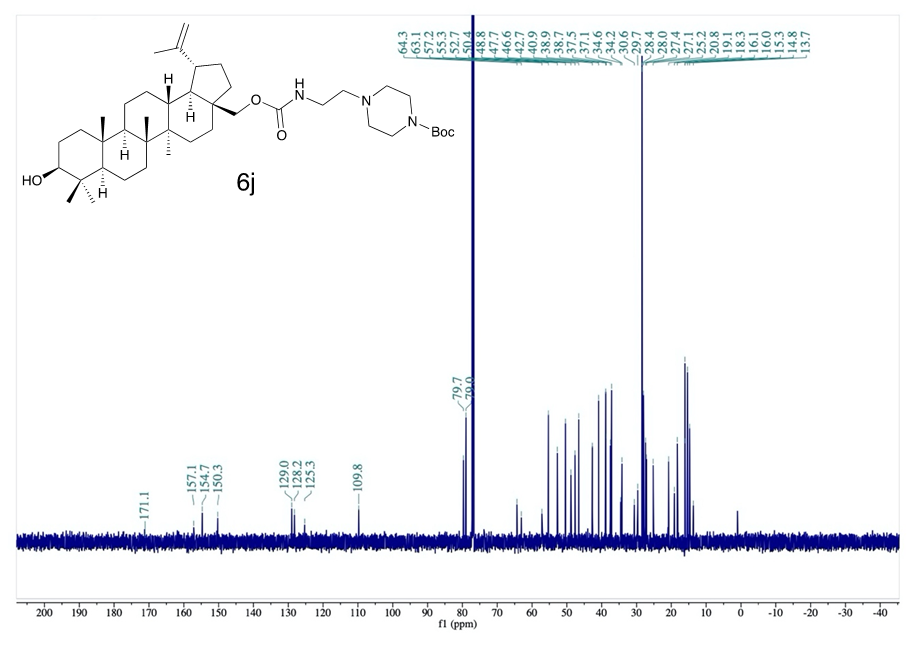


**Fig. S39.** ^13^C-NMR spectrum of compound **6j.**


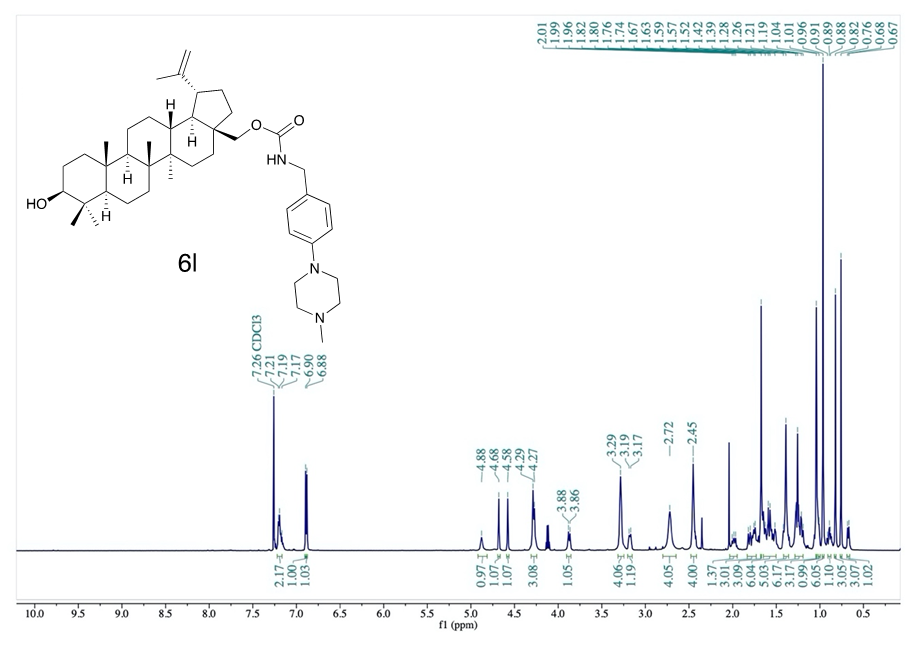


**Fig. S40.** ^1^H-NMR spectrum of compound **6l.**


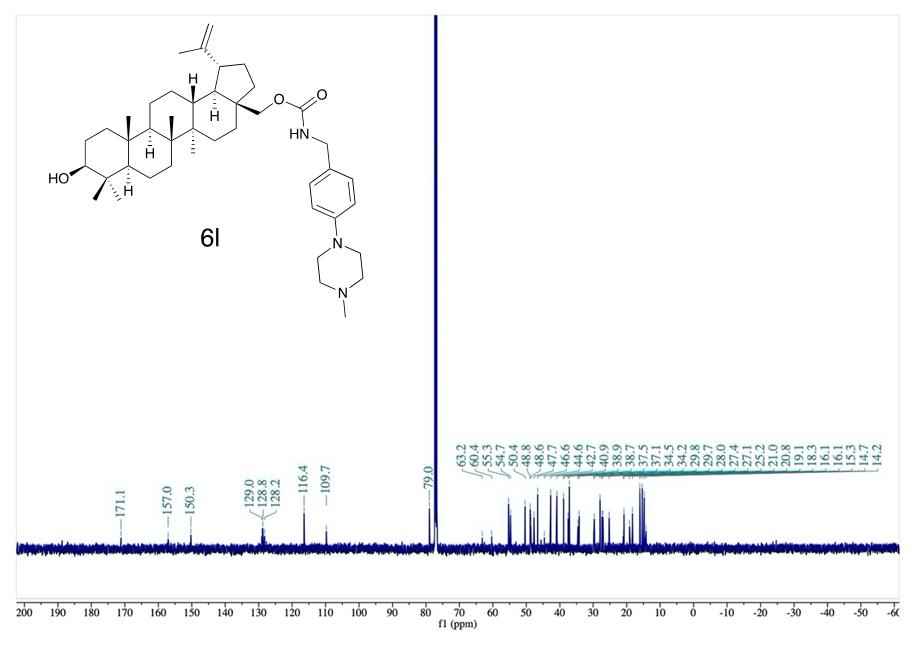


**Fig. S41.** ^13^C-NMR spectrum of compound **6l.**


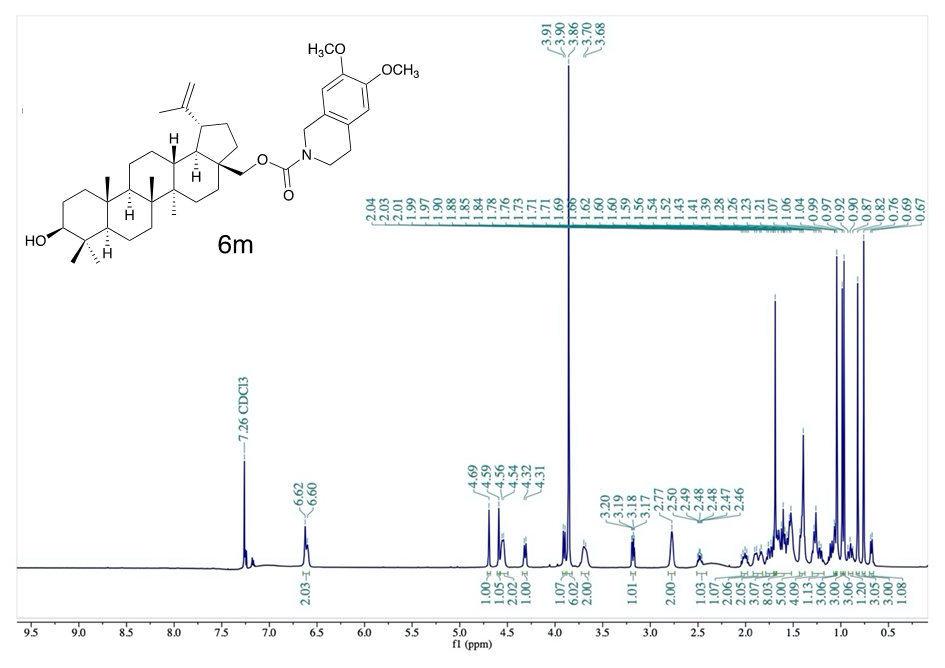


**Fig. S42.** ^1^H-NMR spectrum of compound **6m.**


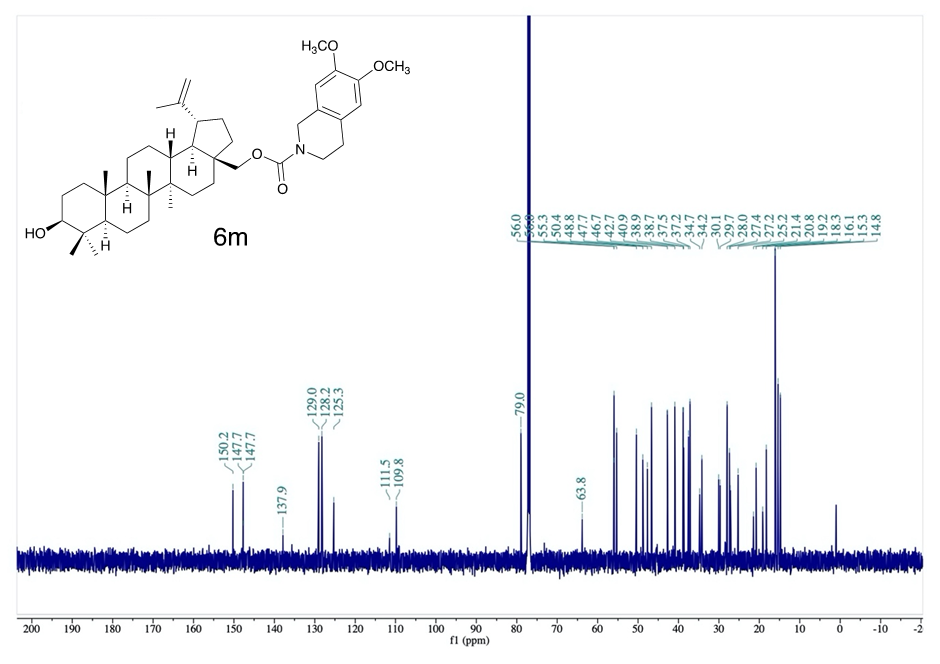


**Fig. S43.** ^13^C-NMR spectrum of compound **6m.**




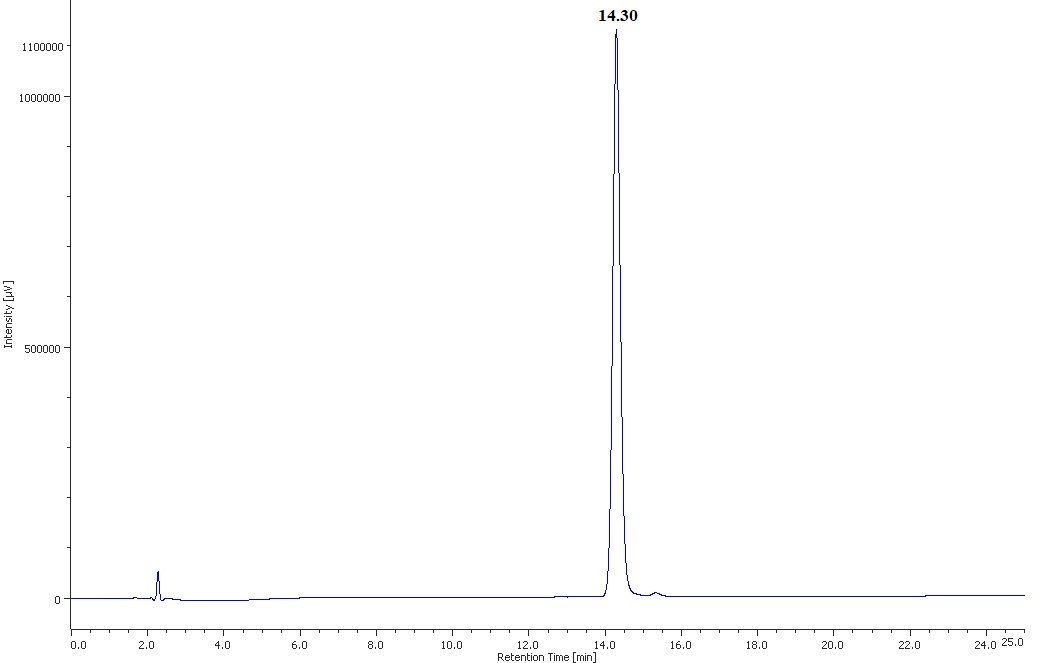


**Fig. S44.** HPLC chromatogram of compound **3a.**




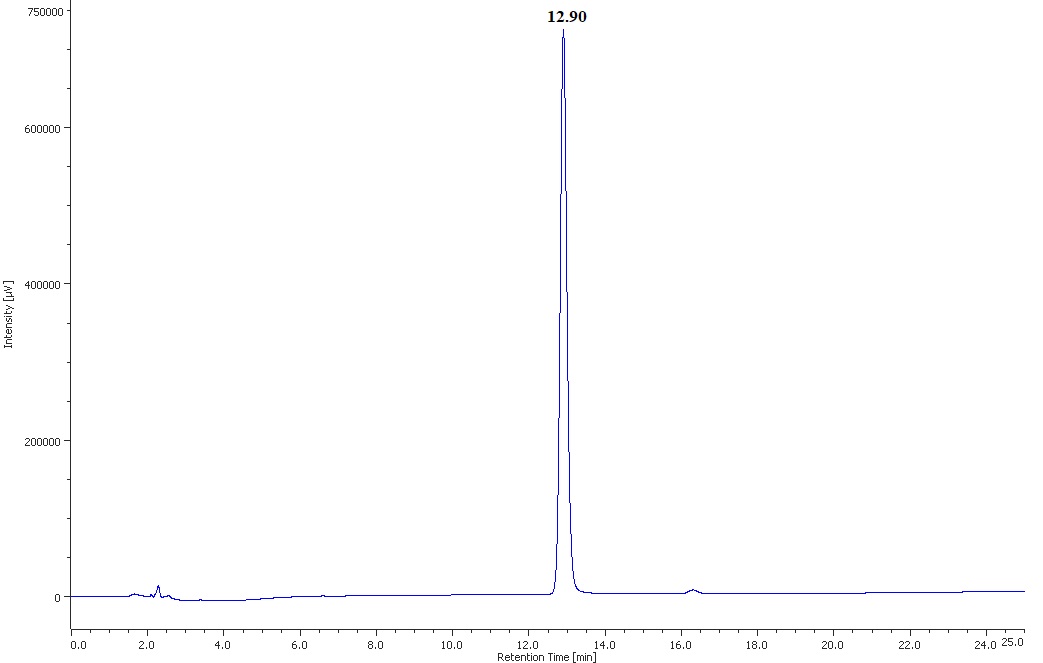


**Fig. S45.** HPLC chromatogram of compound **3b.**




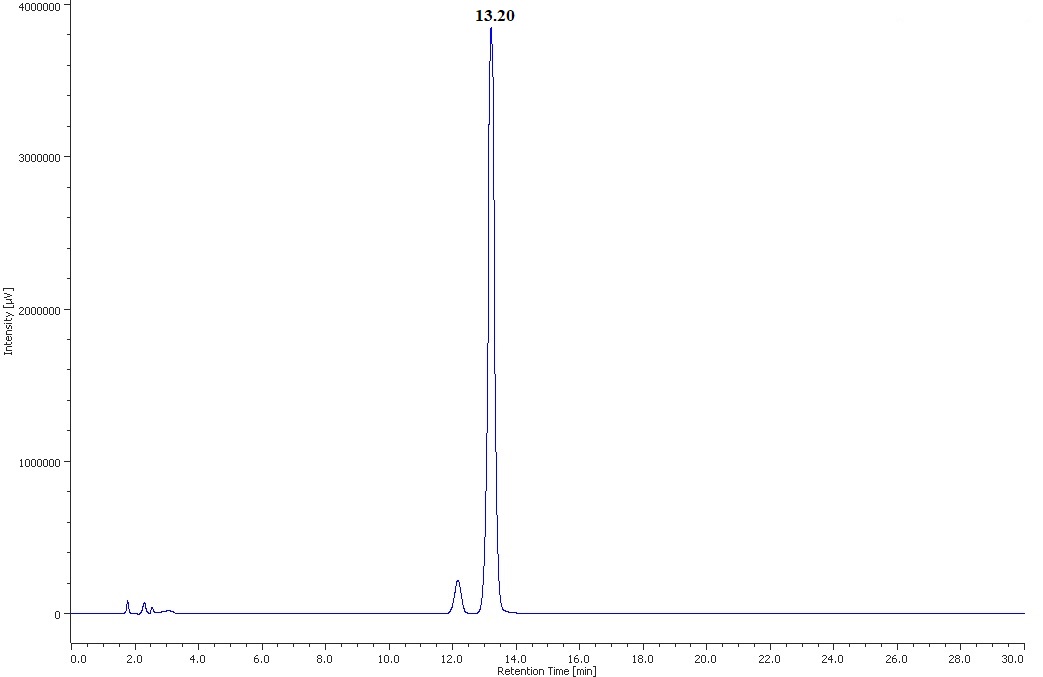


**Fig. S46.** HPLC chromatogram of compound **3c.**




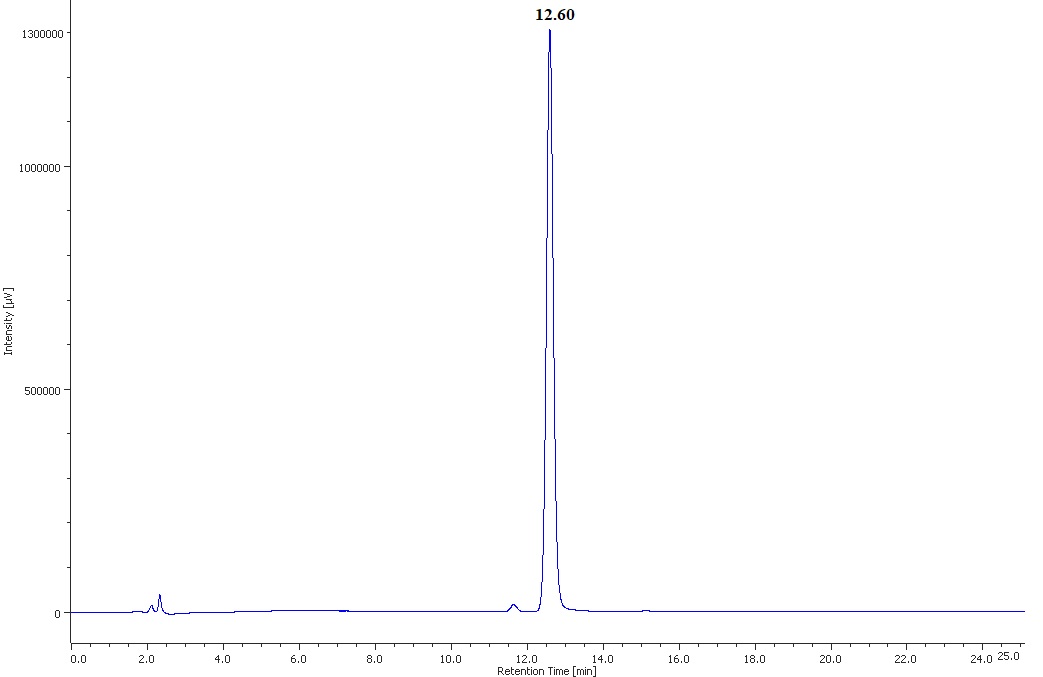


**Fig. S47.** HPLC chromatogram of compound **3d.**




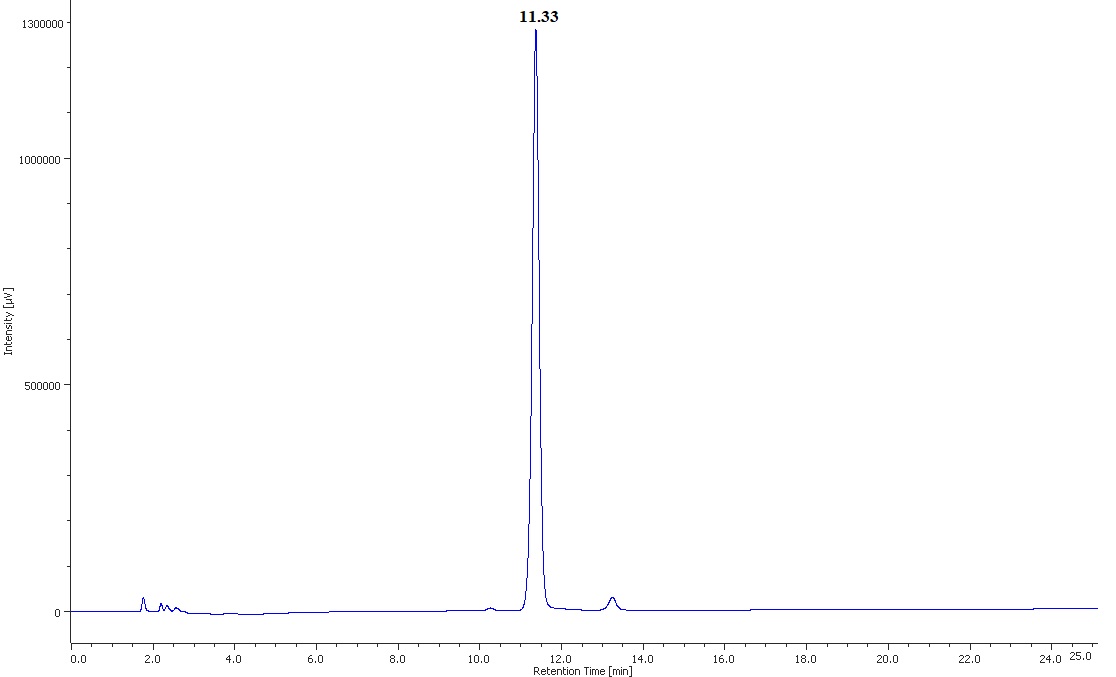


**Fig. S48.** HPLC chromatogram of compound **3e.**




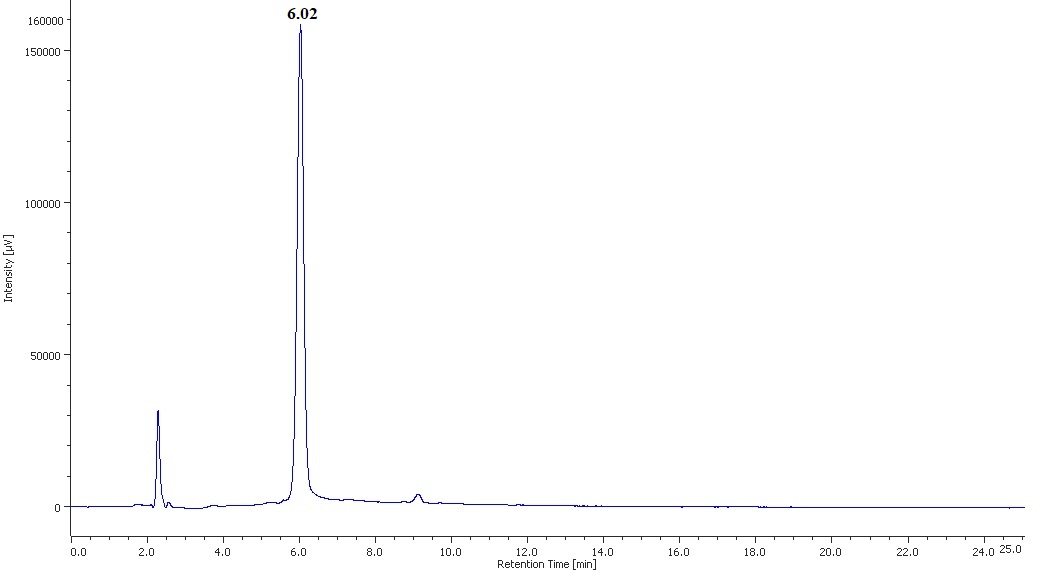


**Fig. S49.** HPLC chromatogram of compound **4.**




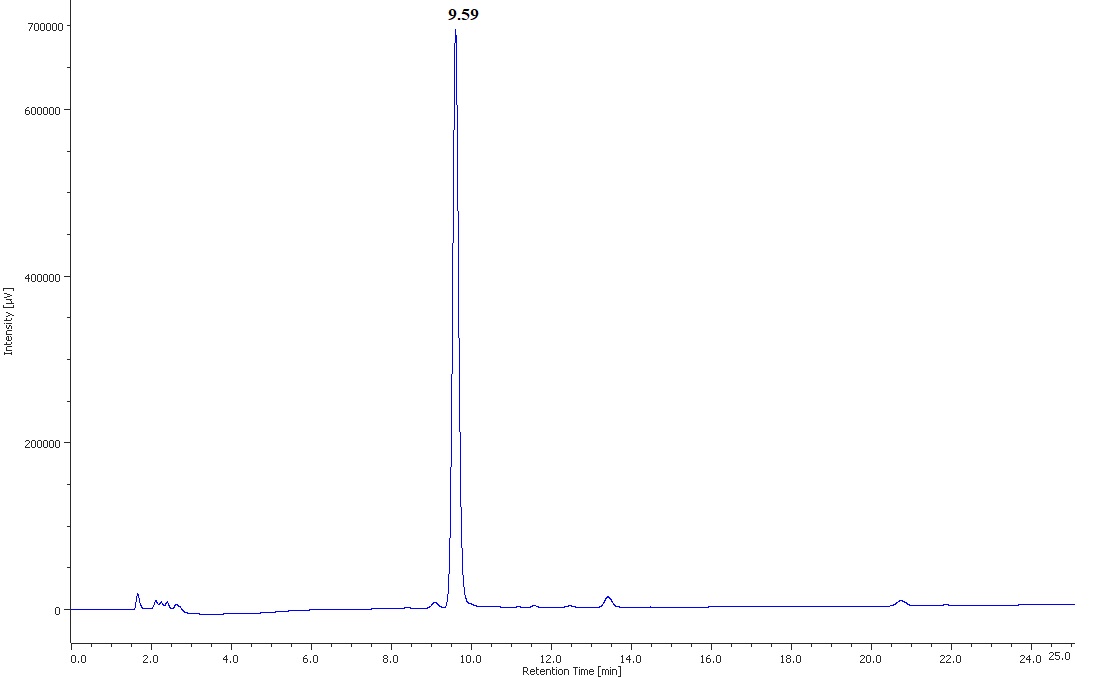


**Fig. S50.** HPLC chromatogram of compound **6a.**




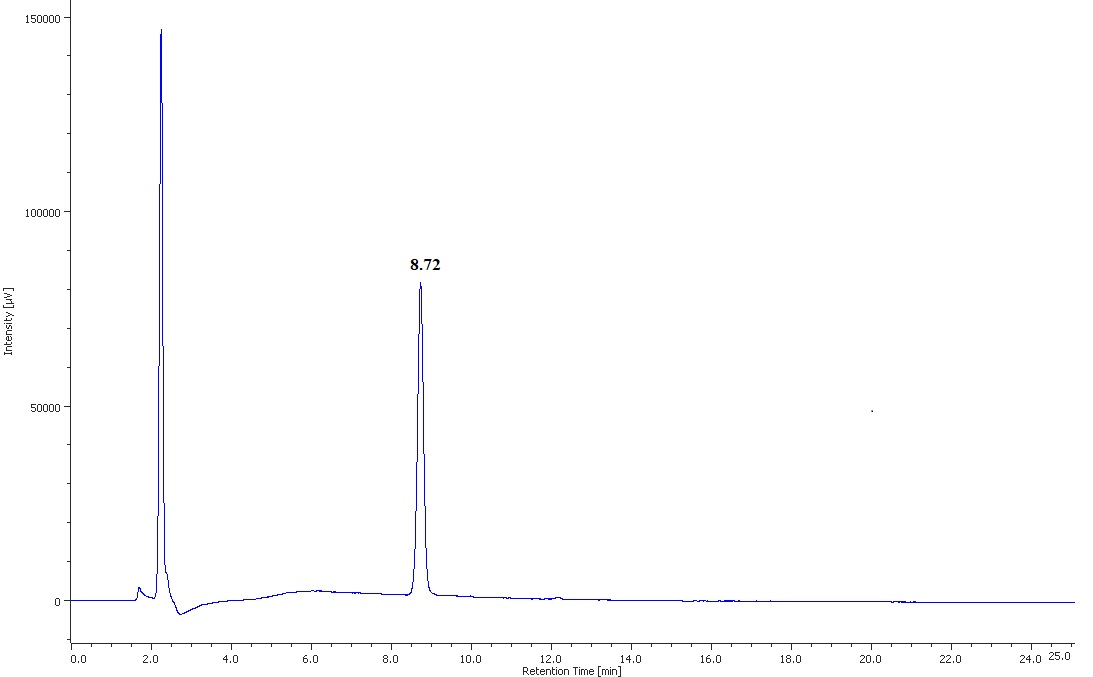


**Fig. S51.** HPLC chromatogram of compound **6b.**




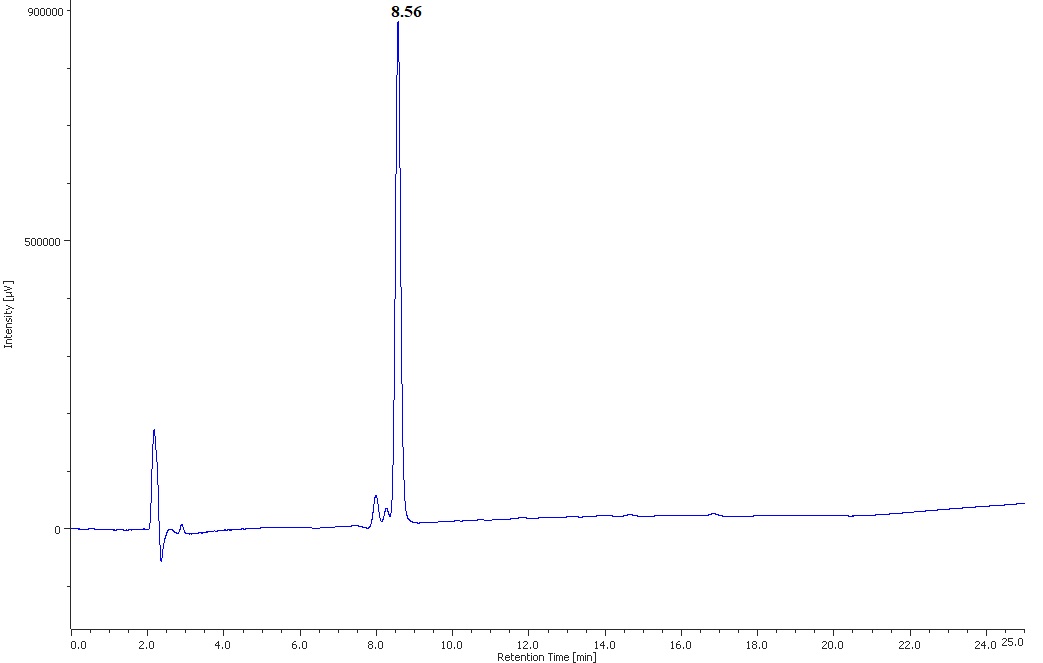


**Fig. S52.** HPLC chromatogram of compound **6c.**




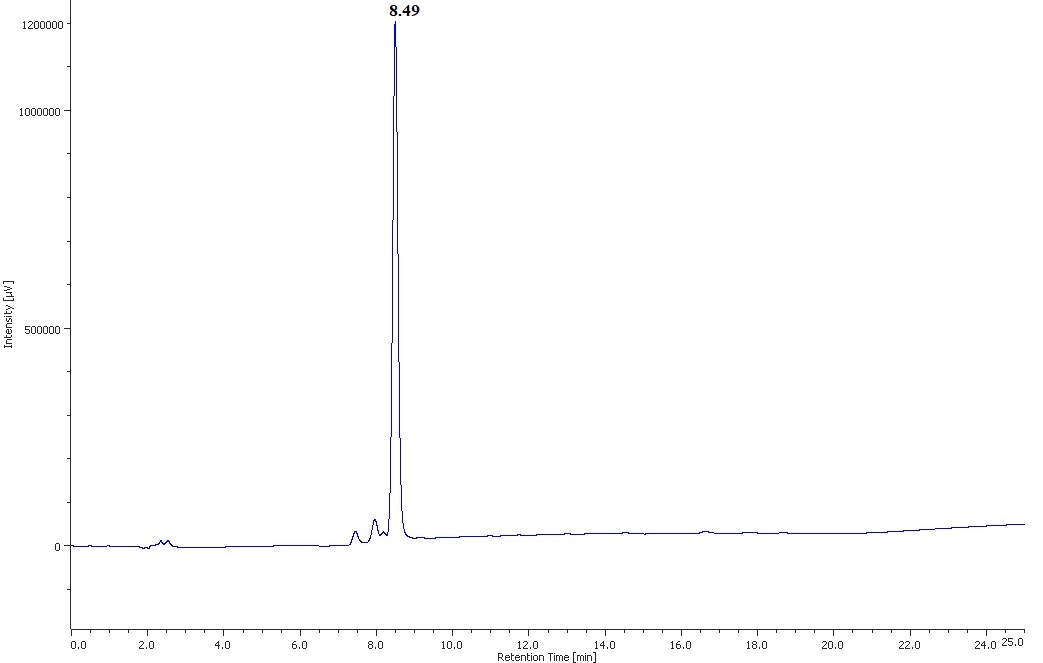


**Fig. S53.** HPLC chromatogram of compound **6d.**




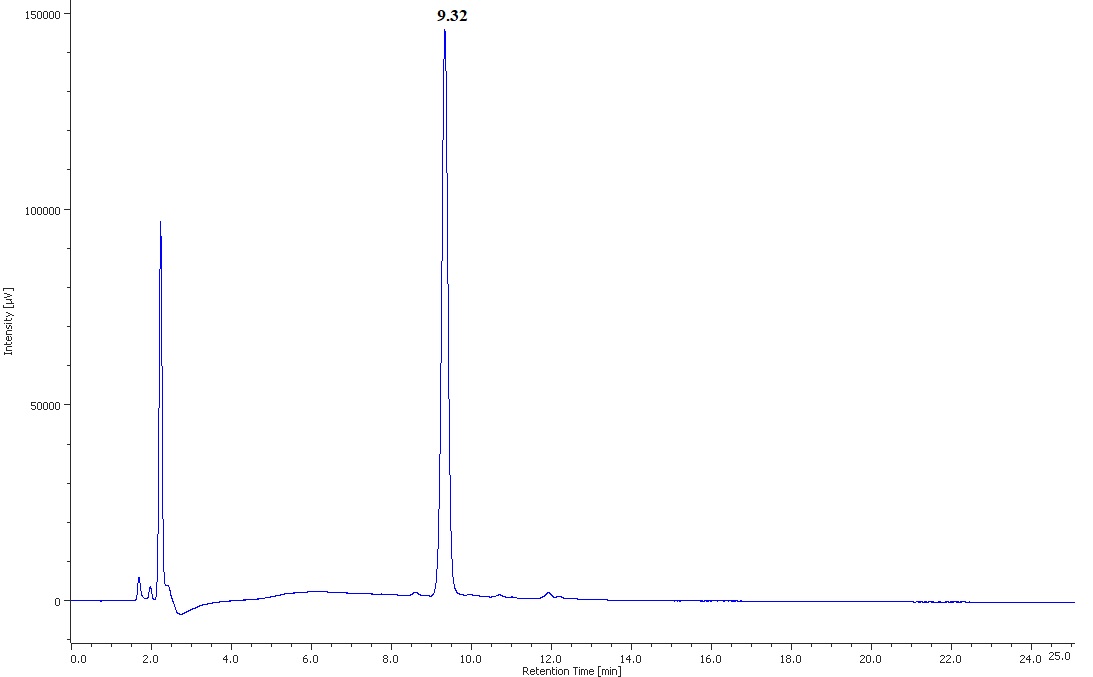


**Fig. S54.** HPLC chromatogram of compound **6e.**




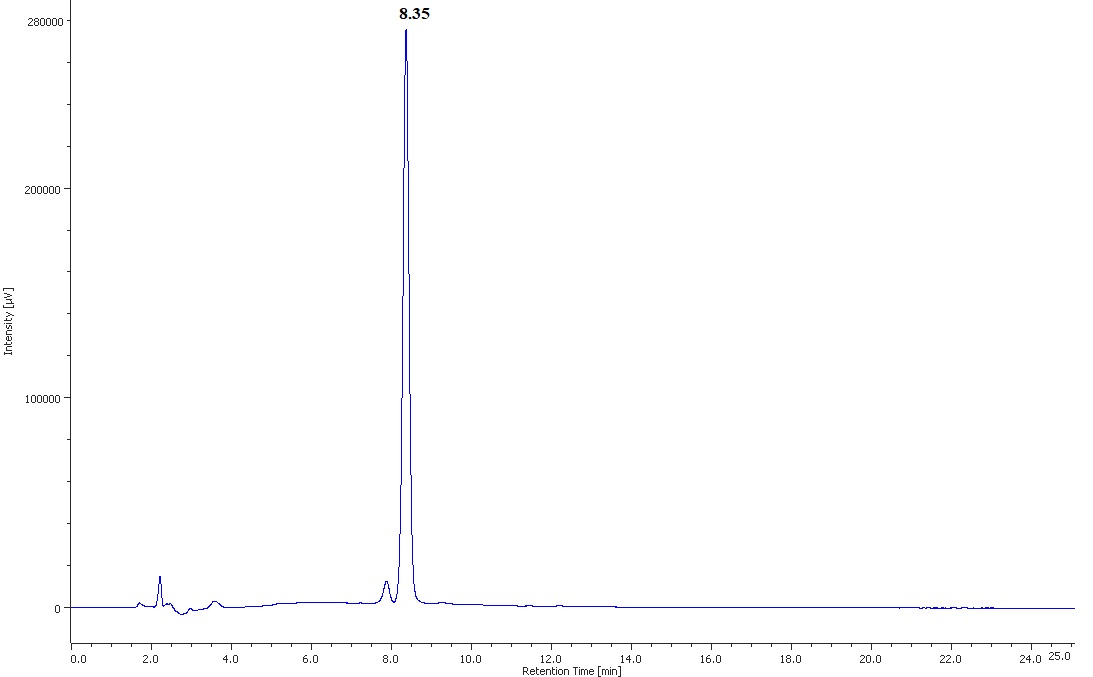


**Fig. S55.** HPLC chromatogram of compound **6f.**




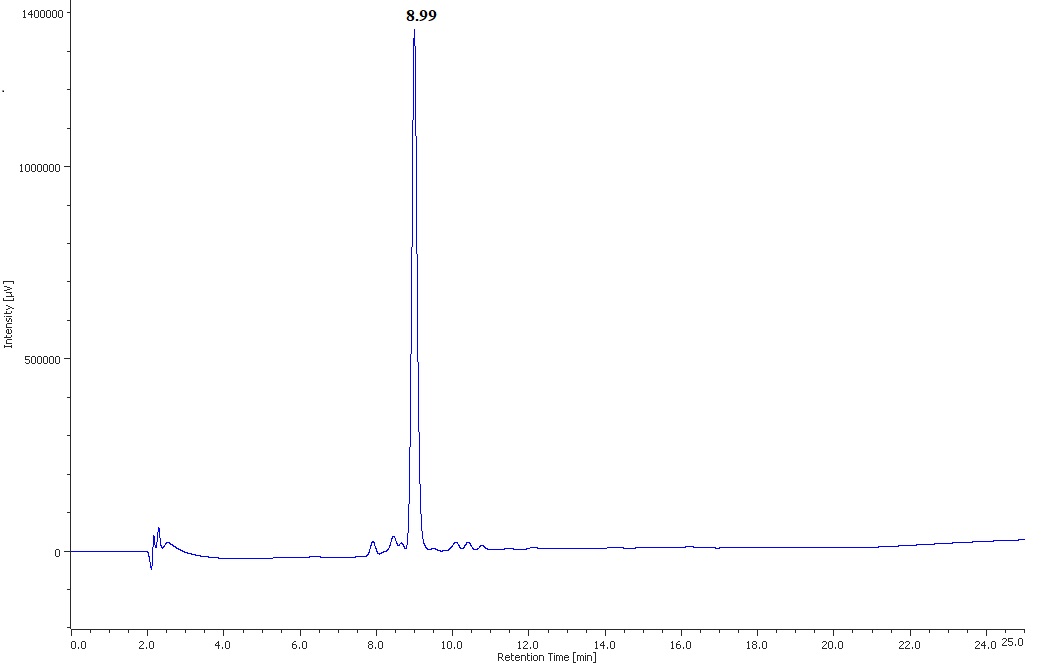


**Fig. S56.** HPLC chromatogram of compound **6g.**




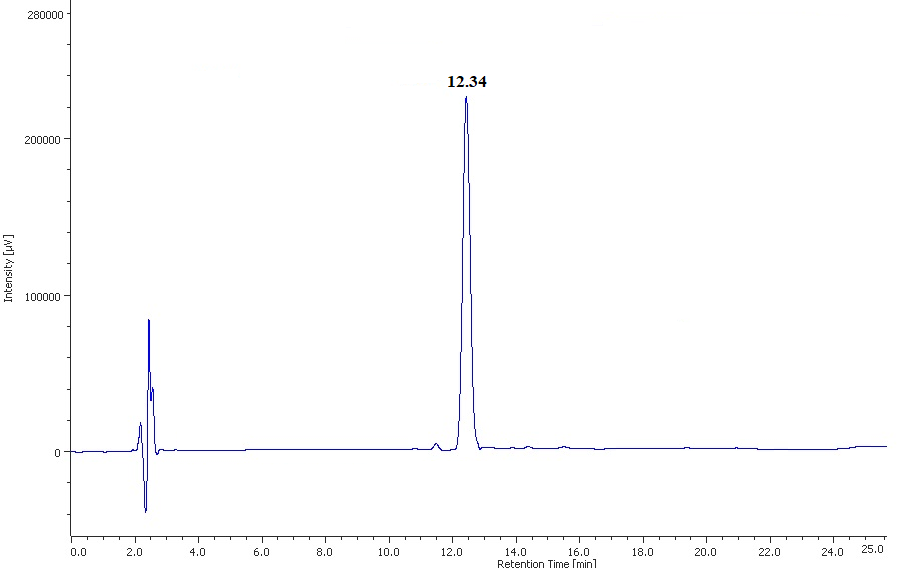


**Fig. S57.** HPLC chromatogram of compound **6h.**




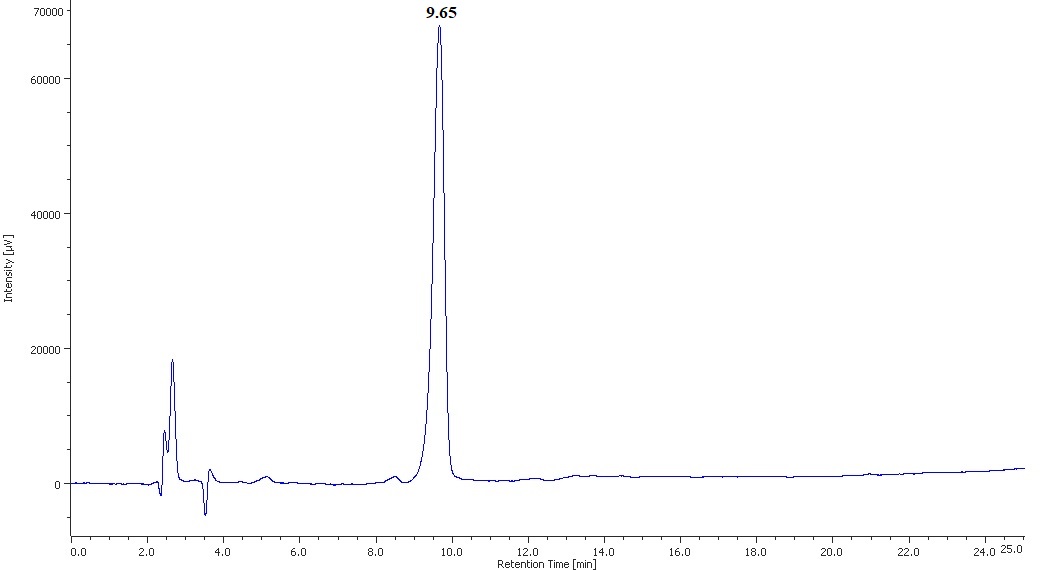


**Fig. S58.** HPLC chromatogram of compound **6i.**




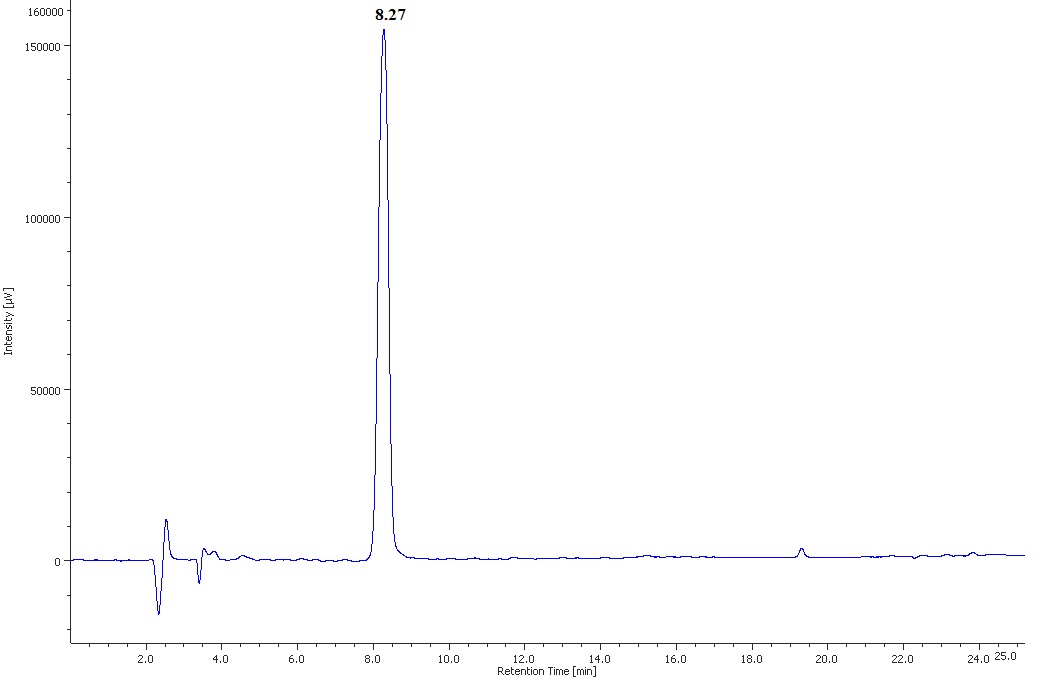


**Fig. S59.** HPLC chromatogram of compound **6j.**




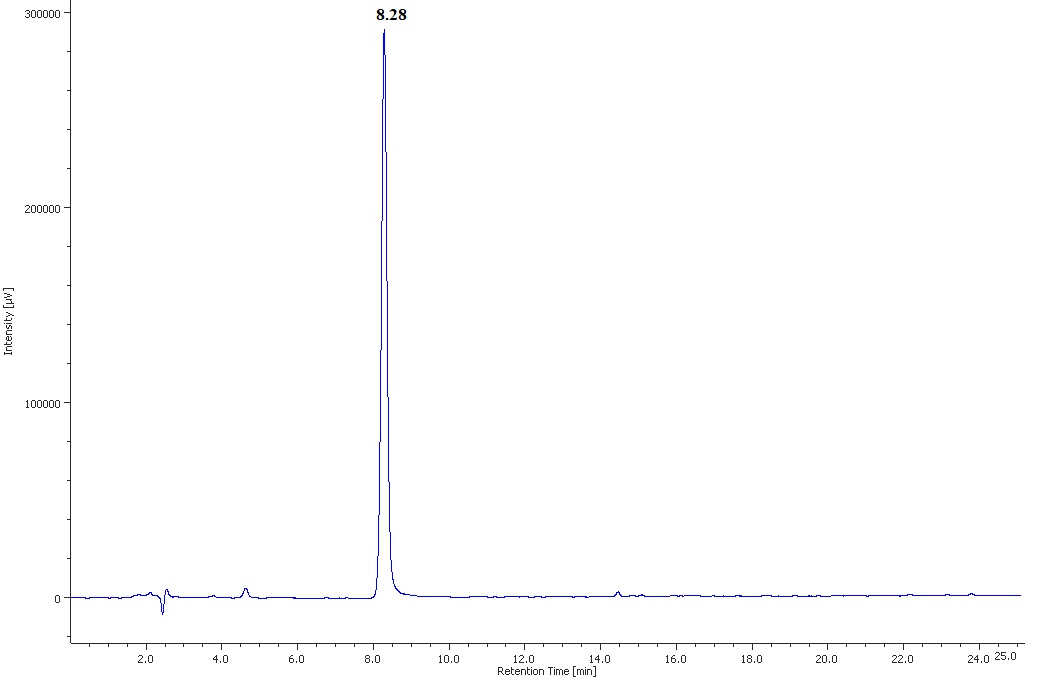


**Fig. S60.** HPLC chromatogram of compound **6k.**




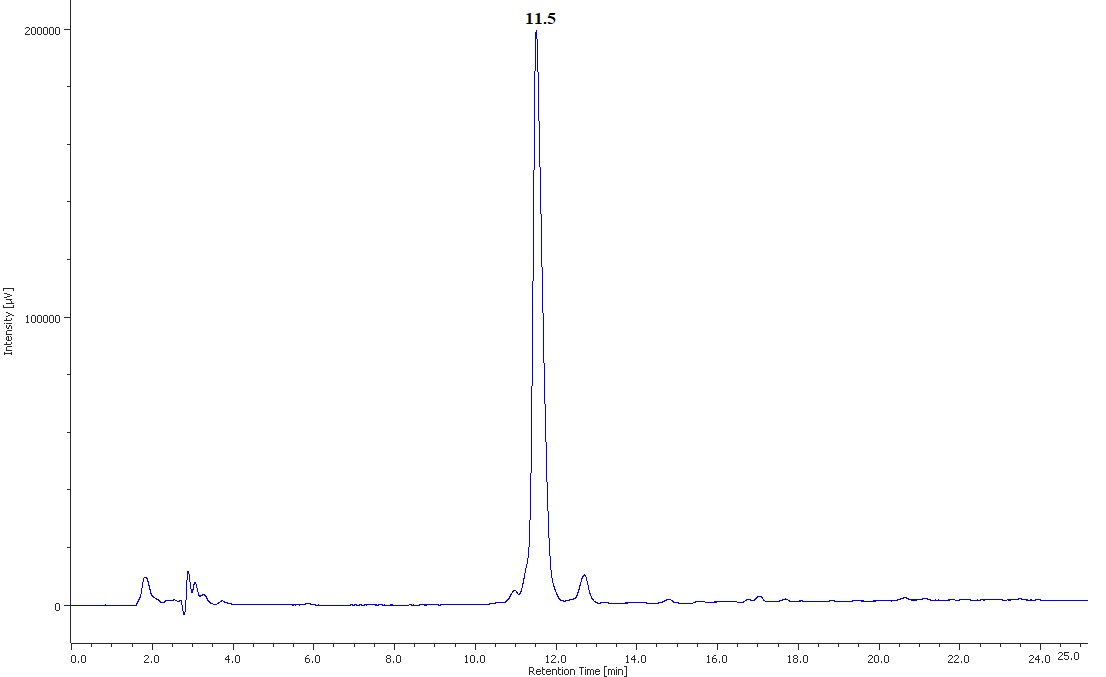


**Fig. S61.** HPLC chromatogram of compound **6l.**




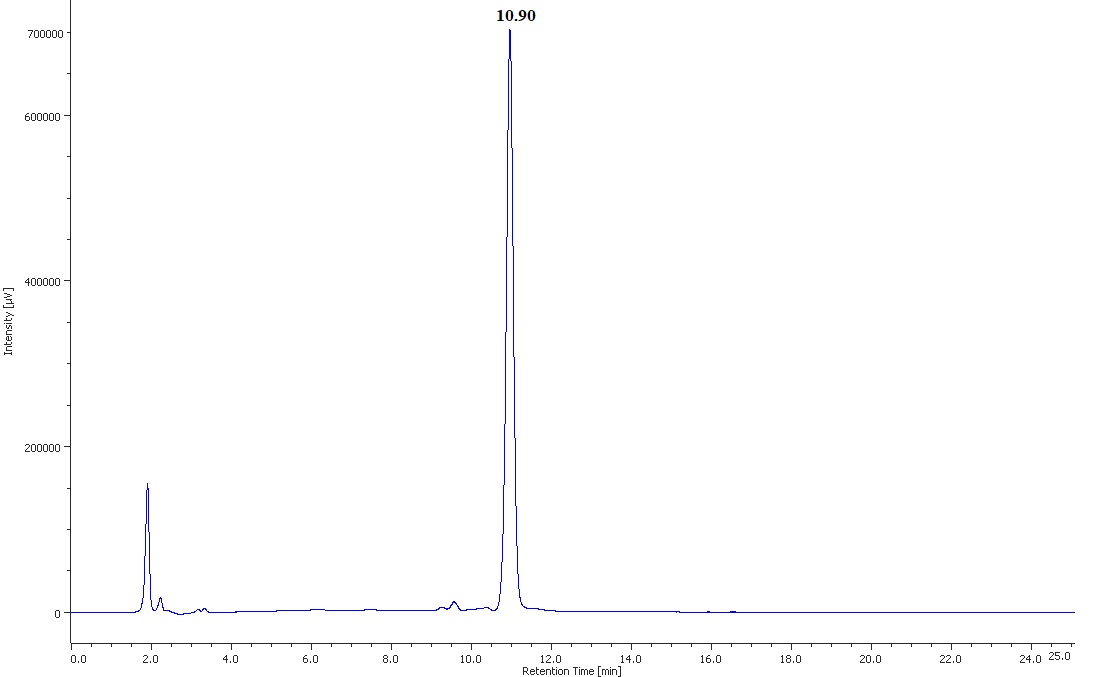


**Fig. S62.** HPLC chromatogram of compound **6m.**

[1] J. Laiolo, P.A. Lanza, O. Parravicini, C. Barbieri, D. Insuasty, J. Cobo, D. Vera, R.D. Enriz, M.C. Carpinella, Structure activity relationships and the binding mode of quinolinone-pyrimidine hybrids as reversal agents of multidrug resistance mediated by P-gp, Sci. Rep., 11 (2021) 1-18.

**Cytotoxicity activity of compounds 4, 6h, 6i, 6k, 6l and tariquidar**


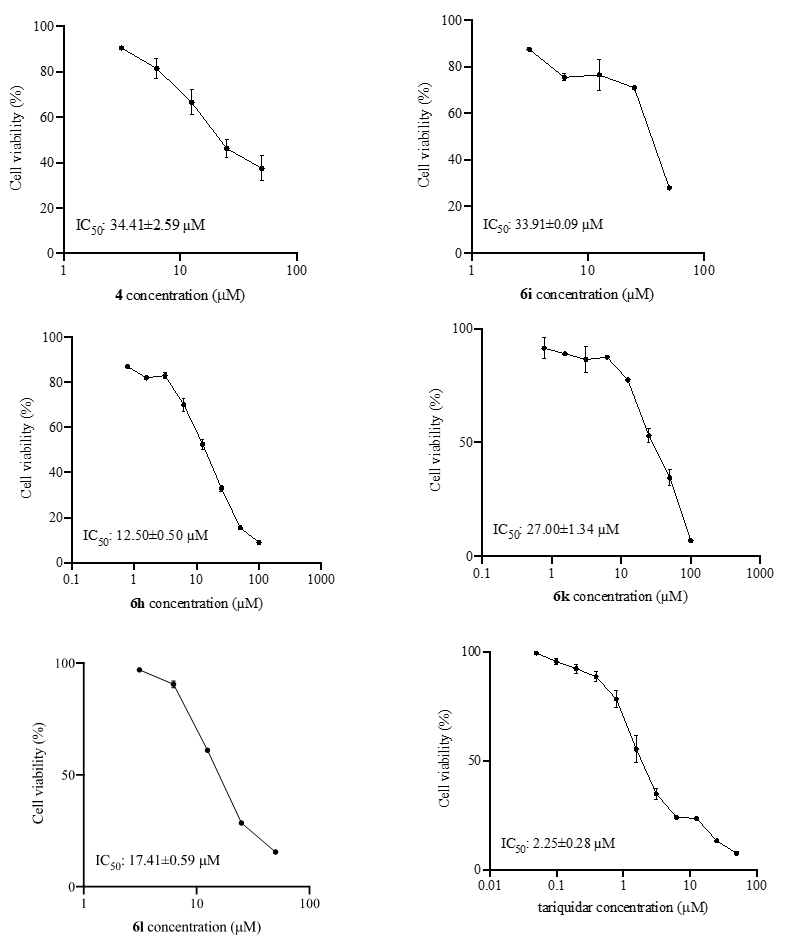


**Fig. S63.** Antiproliferative effect of the derivatives **4**, **6h**, **6i**, **6k**, and **6l** and of tariquidar against multidrug-resistant Lucena 1 cells.


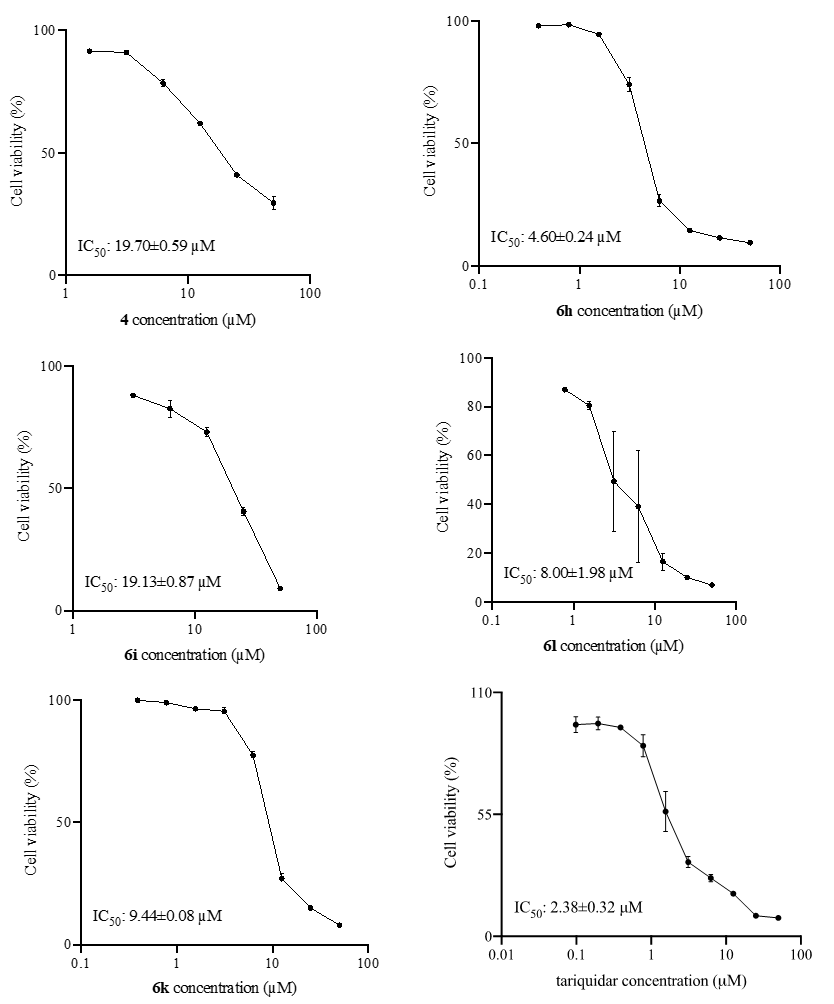


**Fig. S64.** Antiproliferative effect of the derivatives **4**, **6h**, **6i**, **6k**, and **6l** and of tariquidar against sensitive K562 cells.
